# Supplementary material for: Atypical contribution of caspase-3 to melanoma cancer cell motility by regulation of coronin 1B activity
Source: Cell Death Dis. 2025 Oct 6;16(1):690. doi: 10.1038/s41419-025-07952-y (PMC12500911; doi:10.1038/s41419-025-07952-y)
Supplement: Supplementary file 1 — Supplementary figure legends [file 41419_2025_7952_MOESM1_ESM.docx]

**Supplementary figure legends**

**Supplementary Figure 1 – Related to Fig.1**

**(A)** Upper panel: Western blot analysis of CASP3 in WM793, WM852 and A375 cells transfected with *CASP3*-targeting siRNA. HSC70 serves as a loading control. Lower panel: Densitometry analysis of CASP3 expression, relative to HSC70 expression. **(B)** Clustering of the most differentially-expressed genes (DEGs) between parental and CASP3-knockdown WM793 cells, identifying a DEG signature constituted of 310 genes (FDR<0.05 & logFC>|2|). **(C)** Volcano plot illustrating the two gene signatures constituted of DEGs that are the most significantly upregulated or downregulated following CASP3-depletion in WM793. **(D)** Analysis of the overall survival in patients with melanoma from the TCGA dataset displaying a high or low (relative to the median) GSVA score for the signature of genes increased when caspase-3 is knocked-down (50 genes the most upregulated upon CASP3 knock-down) (log rank test; p-value = 0.015). **(E)** The protocol used for identifying interaction protein partners for CASP3 using immunoprecipitation of CASP3-GFP with GFP Traps^®^, which are ready-to-use pull-down reagents consisting of an anti-GFP nanobody coupled to agarose beads. **(F-G)** Validation by immunoblotting of GFP and CASP3-GFP immunoprecipitation using GFP Traps^®^ in GFP- and CASP3-GFP-expressing WM793 (**F**) and WM852 cells (**G**).

**Supplementary Figure 2 – Related to Fig.3**

**(A)** Immunoblotting analysis of CASP3 expression in WM793 transfected for 48 h with various CASP3 siRNAs at the indicated concentration. GAPDH serves as a loading control. **(B)** Quantification of the migration potential of parental and CASP3-knockdown WM793 cells using various siRNAs, through wound area measurement (n=3, one representative experiment shown, two-way ANOVA statistical test; Statistical significance: ns - P > 0.05; * - P ≤ 0.05; ** - P ≤ 0.01; *** - P ≤ 0.001; **** - P ≤ 0.0001). **(C)** Analysis by immunoblotting of CASP3 expression in WM852 transfected for 48 h with various CASP3 siRNAs at the indicated concentration. GAPDH serves as a loading control. **(D)** Quantification of the migration potential of control and CASP3-knockdown WM852 cells using various siRNAs through wound area measurement (n=3, one representative experiment shown, two-way ANOVA statistical test; Statistical significance: ns - P > 0.05; * - P ≤ 0.05; ** - P ≤ 0.01; *** - P ≤ 0.001; **** - P ≤ 0.0001). **(E)** Analysis by immunoblotting of CASP3 expression in CRISPR/Cas9-edited WM793 cells, electroporated with either control or *CASP3*-targetting sgRNAs. HSC70 serves as a loading control. **(F)** Quantification of the invasion potential of CRISPR/Cas9 control and *CASP3*-targeted WM793 cells, through wound area measurement (n=3, one representative experiment shown, two-way ANOVA statistical test; Statistical significance: ns - P > 0.05; * - P ≤ 0.05; ** - P ≤ 0.01; *** - P ≤ 0.001; **** - P ≤ 0.0001). **(G-H)** Same as in E-F, for WM852 cells. **(I)** Immunoblotting analysis for the efficacy of CASP3 and CASP7 siRNA-mediated knock-down. **(J-K)** Quantification of the migration **(J)** and invasion **(K)** potential of control, CASP3- and CASP7-knockdown WM793 cells (n=3, one representative experiment shown, two-way ANOVA statistical test; Statistical significance: ns - P > 0.05; * - P ≤ 0.05; ** - P ≤ 0.01; *** - P ≤ 0.001; **** - P ≤ 0.0001). **(L)** Quantification of the migration potential of control and CASP3-knockdown WM793 cells treated with the pan-caspase inhibitor qVD-OPh (10µM) through wound area measurement (n=3, one representative experiment shown, two-way ANOVA statistical test; Statistical significance: ns - P > 0.05; * - P ≤ 0.05; ** - P ≤ 0.01; *** - P ≤ 0.001; **** - P ≤ 0.0001). **(M)** Measurement of caspase-3/7 activation in WM793 cells treated with actinomycin D (ActD, 1 µM), ABT-263 (5 µM) and qVD-OPh (10 µM) for 24 h. **(N)** Quantification of the migration potential of WM793 cells treated or not with the CASP3 inhibitor Ac-DEVD-CHO (n=3, one representative experiment shown, two-way ANOVA statistical test; Statistical significance: ns - P > 0.05; * - P ≤ 0.05; ** - P ≤ 0.01; *** - P ≤ 0.001; **** - P ≤ 0.0001). **(O)** Measurement of caspase-3/7 activation in WM793 cells treated with actinomycin D (ActD, 1 µM) and Ac-DEVD-CHO (10 µM) for 24 h. **(P)** WM793 cells expressing a degradation sensitive BCL-xL transgene (WM793-BCLxL-DD cells) were treated with Shield-1 (100 nM) to stabilize and thus upregulate BCL-xL protein expression. **(Q)** Quantification of the migration potential of WM793 cells (control and overexpressing BCL-xL protein) (n=3, one representative experiment shown, two-way ANOVA statistical test; Statistical significance: ns - P > 0.05; * - P ≤ 0.05; ** - P ≤ 0.01; *** - P ≤ 0.001; **** - P ≤ 0.0001). **(R)** Immunoblotting analysis for the increased expression of XIAP protein (via a tetON expression vector and doxycycline -dox- treatment) in WM793 and WM852 cells. **(S, T)** Quantification of the migration potential of WM793 **(S)** and WM852 cells **(T)** overexpressing XIAP protein (n=3, one representative experiment shown, two-way ANOVA statistical test; Statistical significance: ns - P > 0.05; * - P ≤ 0.05; ** - P ≤ 0.01; *** - P ≤ 0.001; **** - P ≤ 0.0001).

**Supplementary Figure 3 – Related to Fig.5**

**(A)** Summary of key steps of the proximity labelling protocol for identifying protein interacting partners of CASP3 fused with BioID2 through the immunoprecipitation of biotinylated proteins with streptavidin beads, following biotin treatment. **(B, C)** Validation of myc tagged-BioID2, -BioID2-CASP3 and -CASP3-BioID2 conditional overexpression following doxycycline treatment (1 µg/mL for 24 h) using anti-Myc antibody in WM793 cells (**B**). Analysis of biotinylated proteins following biotin treatment (50 µM, 12 h) of BioID2, BioID2-CASP3 and CASP3-BioID2-expressing WM793 cells (**C**). (**D, E**) Same as in B, C, for WM852 cells. **(F)** Summary table with the most frequent CASP3-interacting proteins identified in WM852 cells by CASP3-GFP pulldown and proximity labelling through biotinylation of neighboring proteins in cells expressing BioID2-fused CASP3. White circle: absence of hit; green circle: presence of hit.  **(G)** Analysis of CASP3-GFP and CORO1B/P-CORO1B interaction after immunoprecipitation (IP) of GFP protein complexes in GFP- or CASP3-GFP in WM852 melanoma cells. **(H)** Analysis of the proximity between endogenous CASP3 and CORO1B proteins in CRISPR/Cas9 control and *CASP3*-targetted WM793 cells, using a Proximity Ligation Assay (PLA). **(I)** Quantification of PLA dots in CRISPR/Cas9 control (n = 35 cells, in a representative experiment) and CASP3-targeted (n = 33 cells) WM793 cells. **(J)** Validation of the efficacy of two different siRNAs for CORO1B in WM793 cells. **(K)** Analysis by immunoblotting of P-CORO1B, CORO1B, P-PKCα, PKCα, ARP2/3, CASP3 in WM852 transfected with two different siRNAs for CASP3 and CORO1B. HSC70 serves as a loading control.  **(L)** Left panel: Analysis of P-CORO1B and F-actin localization by immunofluorescence in parental and CASP3-knockdown WM793 cells. Right panel: Signal intensity measurement of P-CORO1B and F-actin in the indicated corresponding line in control and CASP3-knockdown WM793 in the left panel. **(M)** Quantification of P-CORO1B immunostaining intensity in proximity to plasma membrane and in cytosol, between control and CASP3-depleted cells (n=3, paired t test; Statistical significance: ns - P > 0.05; * - P ≤ 0.05; ** - P ≤ 0.01; *** - P ≤ 0.001; **** - P ≤ 0.0001). **(N)** Analysis by immunoblotting of P-CORO1B, CORO1B, CASP3 in parental and CASP3-knockdown WM793 and WM852 cells treated with PMA (100 nM) for the indicated time. HSC70 and GAPDH serve as a loading control. **(O)** Analysis by immunoblotting of CORO1B, CASP3 and MCL1 in control and CASP3-knockdown WM793 and WM852 cells treated with cycloheximide (50 µg/mL) for the indicated time. GAPDH serves as a loading control.

**Supplementary Figure 4– Related to Fig.6**

**(A)** *CASP3* mRNA expression in control and SP1-knockdown WM852 cells relative to *GAPDH* (Arbitrary Unit). **(B)** Western blot analysis of CASP3 and SP1 in control and SP1-knockdown WM852 cells. HSC70 serves as a loading control. **(C-D)** *CASP3* mRNA expression in WM793 (**C**) and WM852 cells (**D**) treated with increasing doses of mithramycin A (100 nM, 200 nM and 300 nM for 24 h) relative to *GAPDH*.

**Supplementary Table 1 -** Primer List
