# Supplementary figures and images for: Atypical contribution of caspase-3 to melanoma cancer cell motility by regulation of coronin 1B activity
Source: Cell Death Dis. 2025 Oct 6;16(1):690. doi: 10.1038/s41419-025-07952-y (PMC12500911; doi:10.1038/s41419-025-07952-y)

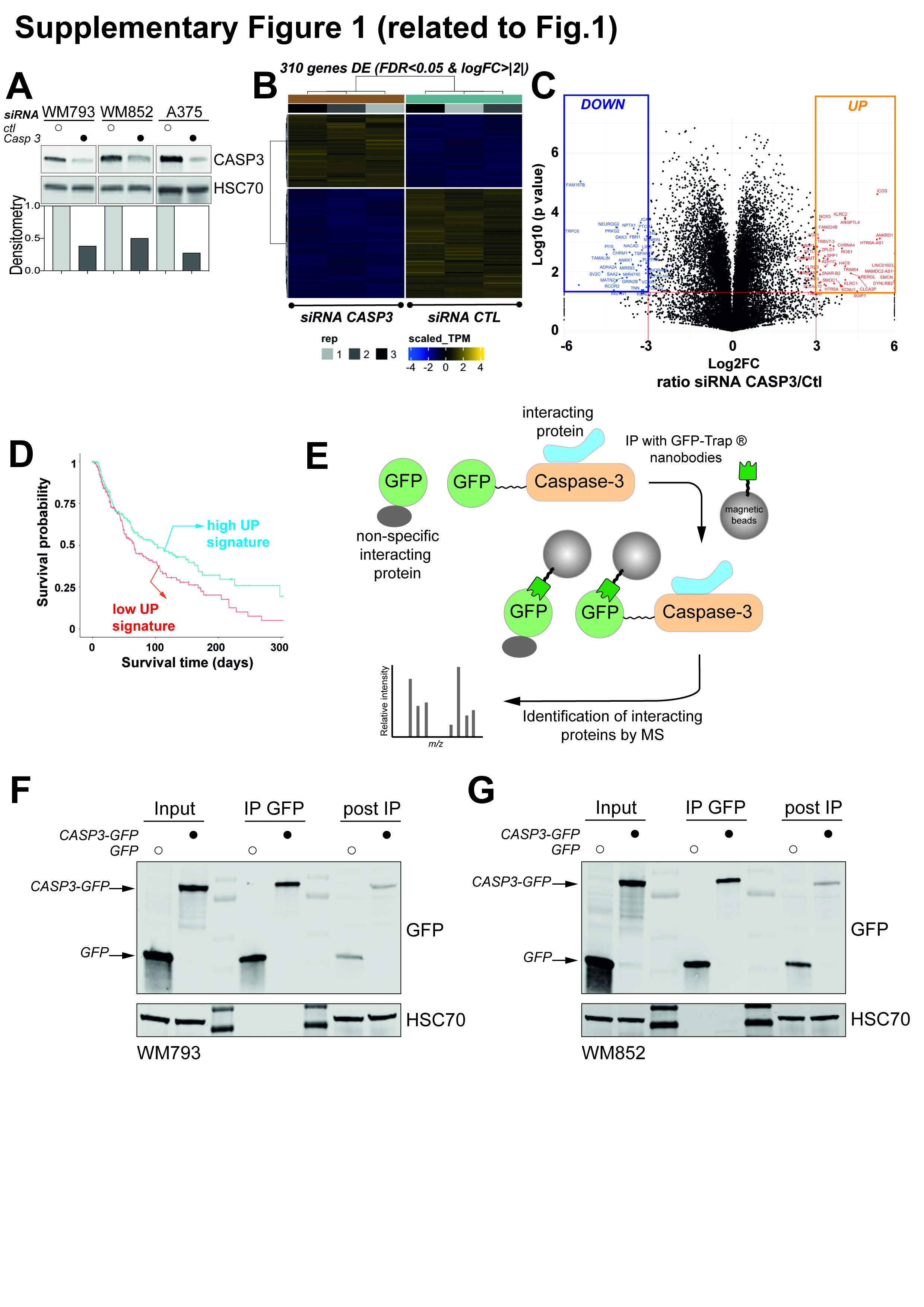

Supplement: Supplementary file 2 — Supplementary Figure 1 [file 41419_2025_7952_MOESM2_ESM.tif]

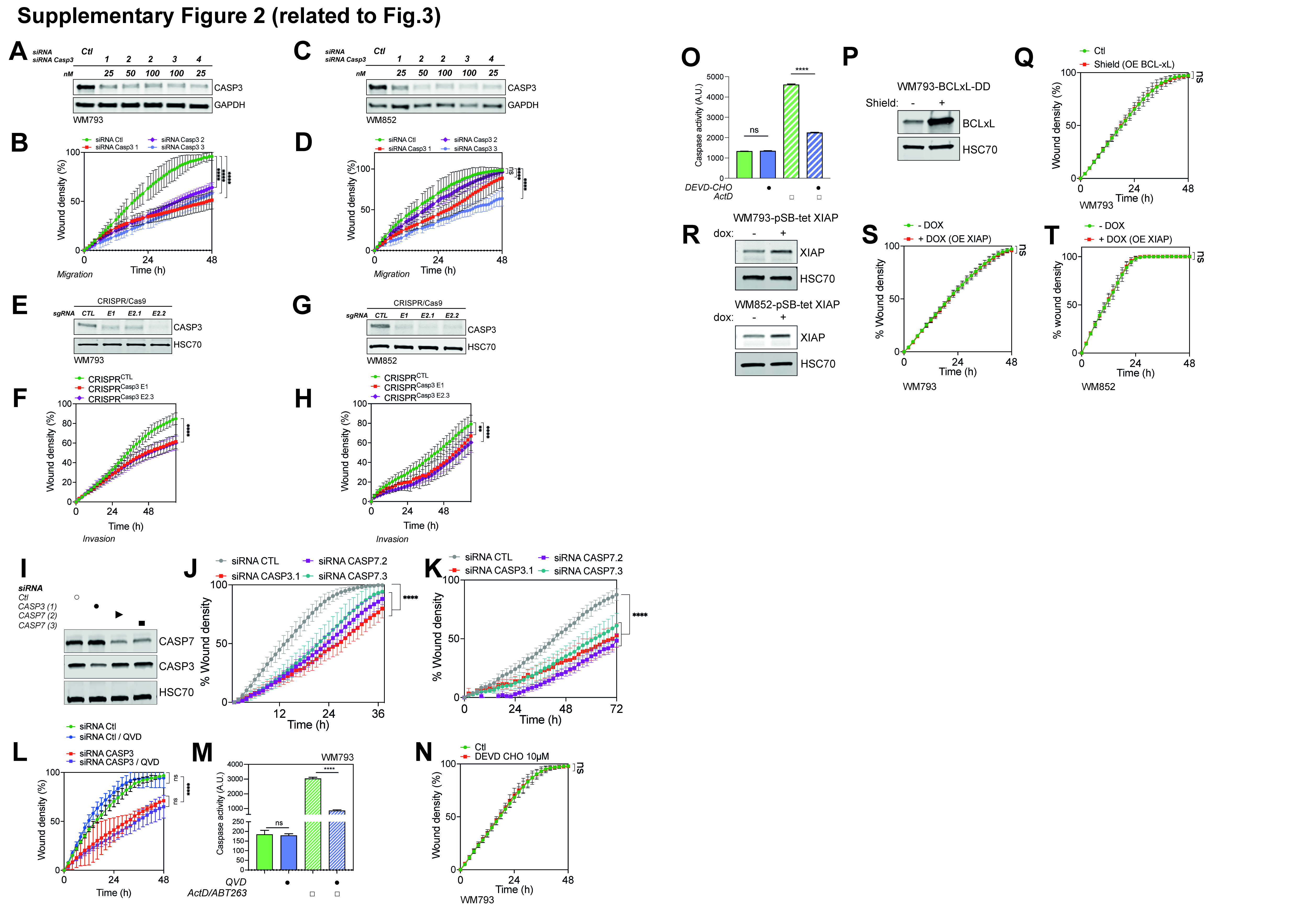

Supplement: Supplementary file 3 — Supplementary Figure 2 [file 41419_2025_7952_MOESM3_ESM.tif]

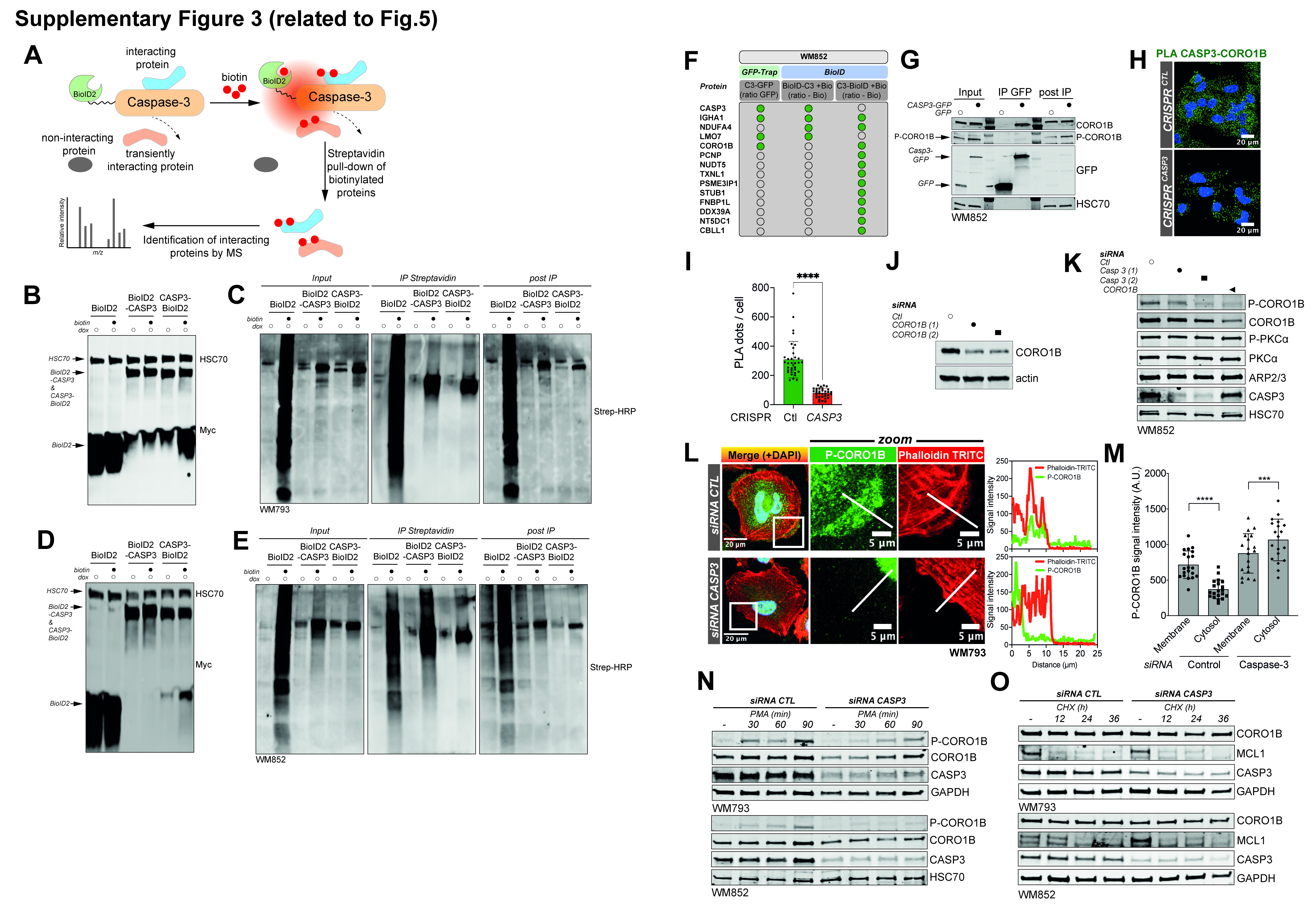

Supplement: Supplementary file 4 — Supplementary Figure 3 [file 41419_2025_7952_MOESM4_ESM.tif]

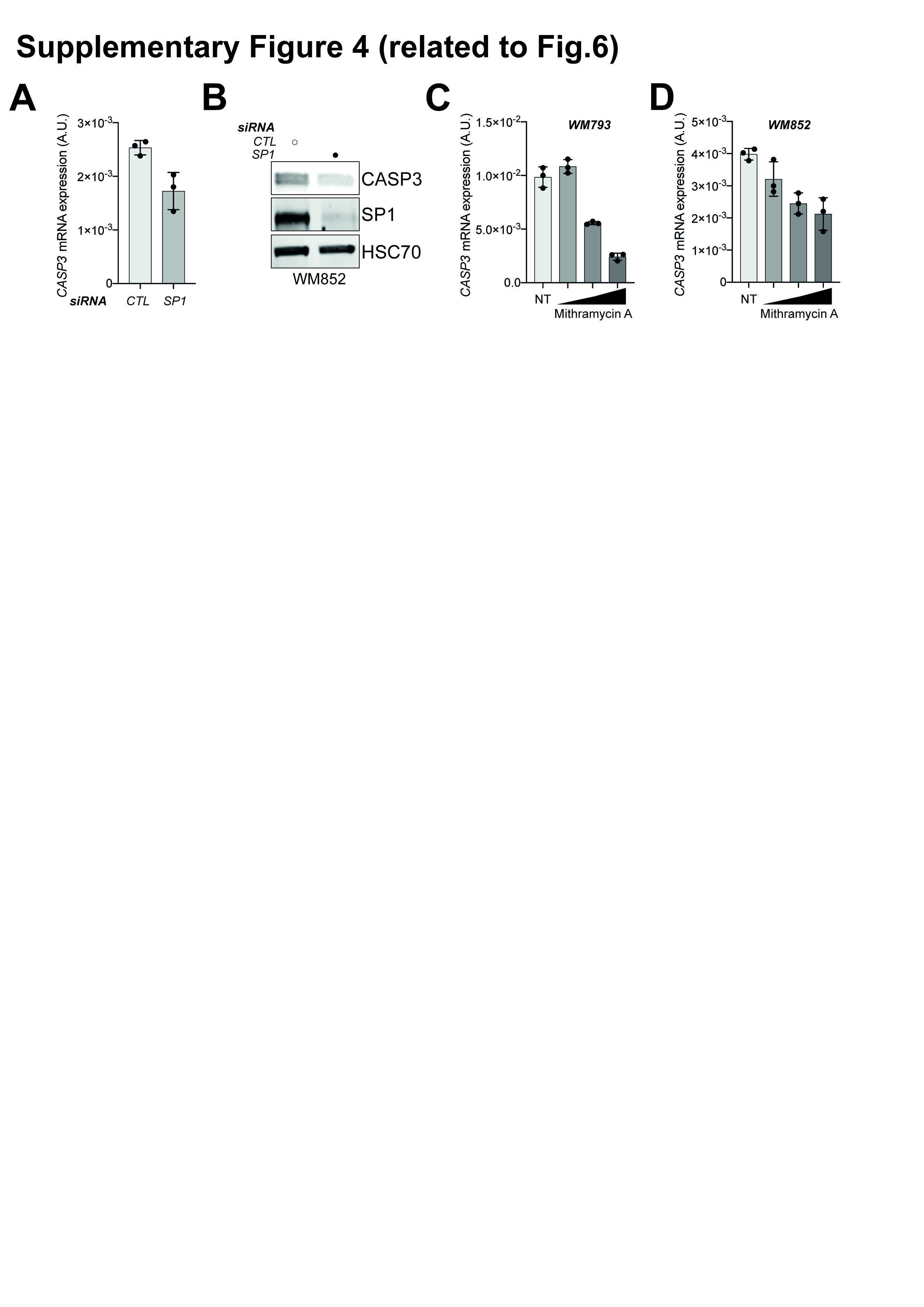

Supplement: Supplementary file 5 — Supplementary Figure 4 [file 41419_2025_7952_MOESM5_ESM.tif]
