# Supplementary material for: Atypical contribution of caspase-3 to melanoma cancer cell motility by regulation of coronin 1B activity
Source: Cell Death Dis. 2025 Oct 6;16(1):690. doi: 10.1038/s41419-025-07952-y (PMC12500911; doi:10.1038/s41419-025-07952-y)

Figure 2C ***Article version***

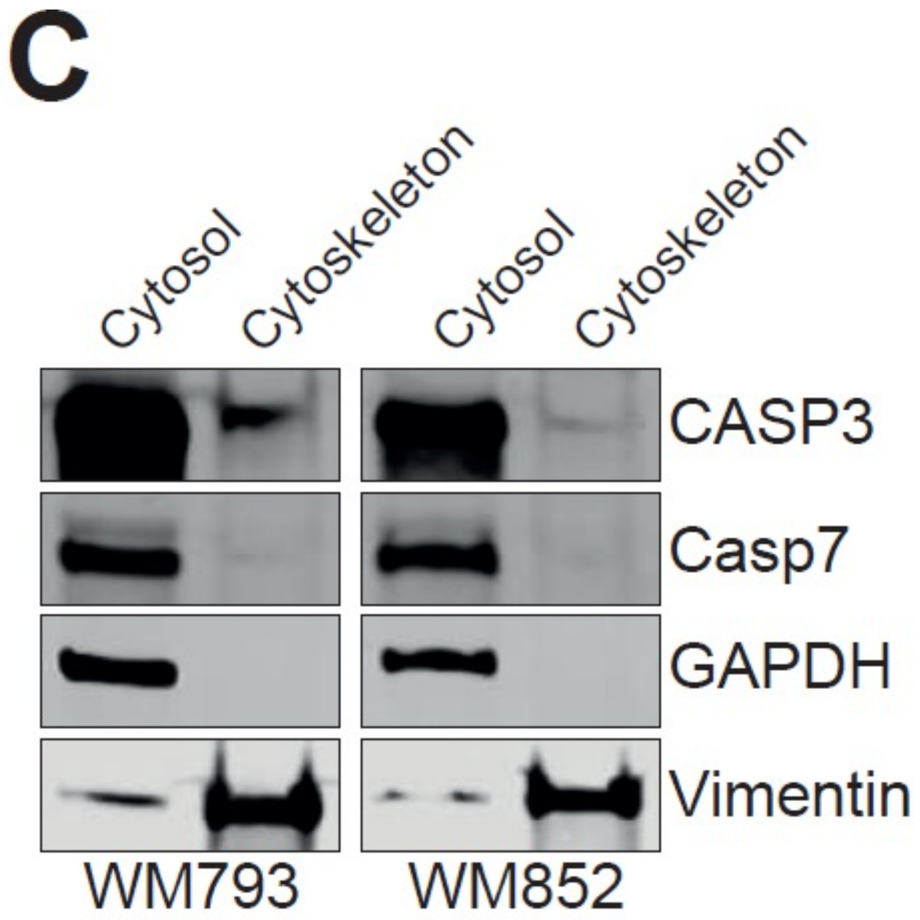

Figure 2C *Raw data*

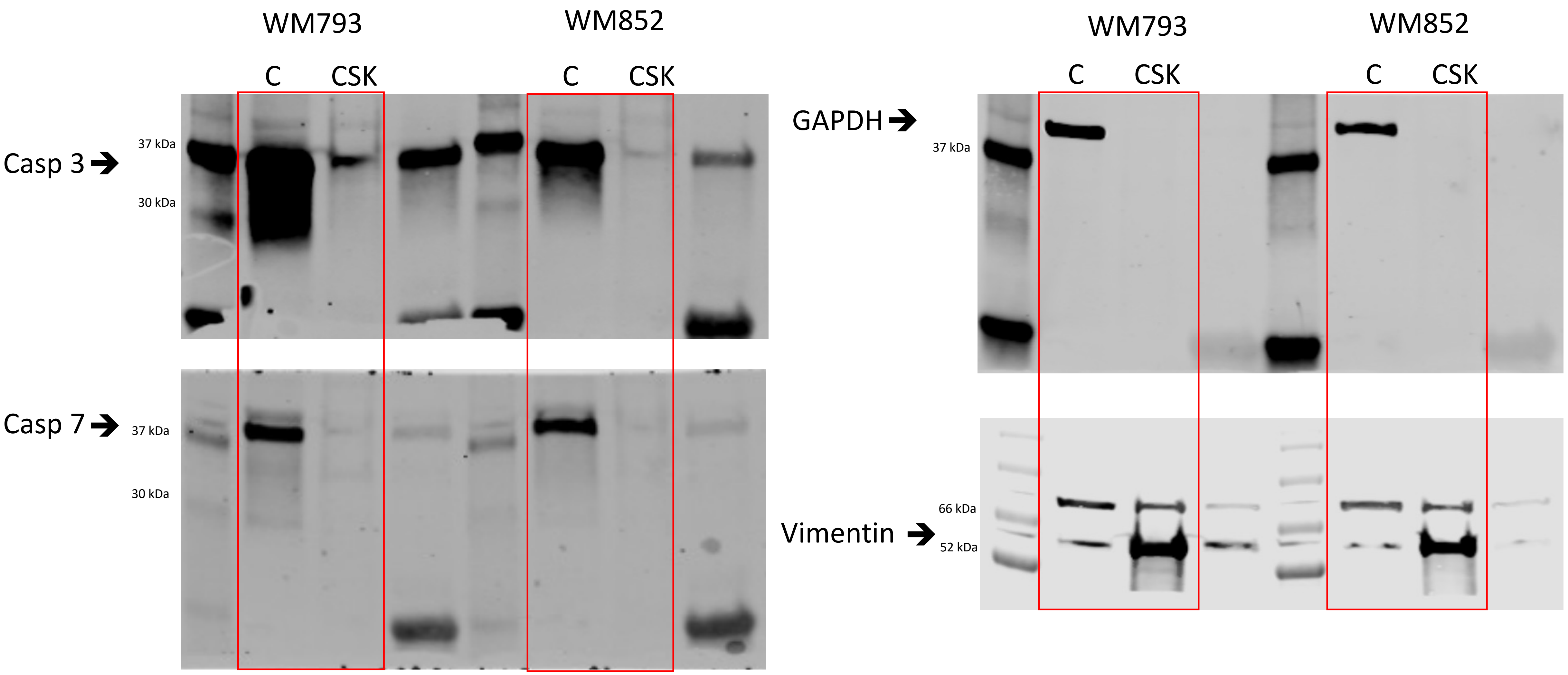

Figure 4B ***Article version***

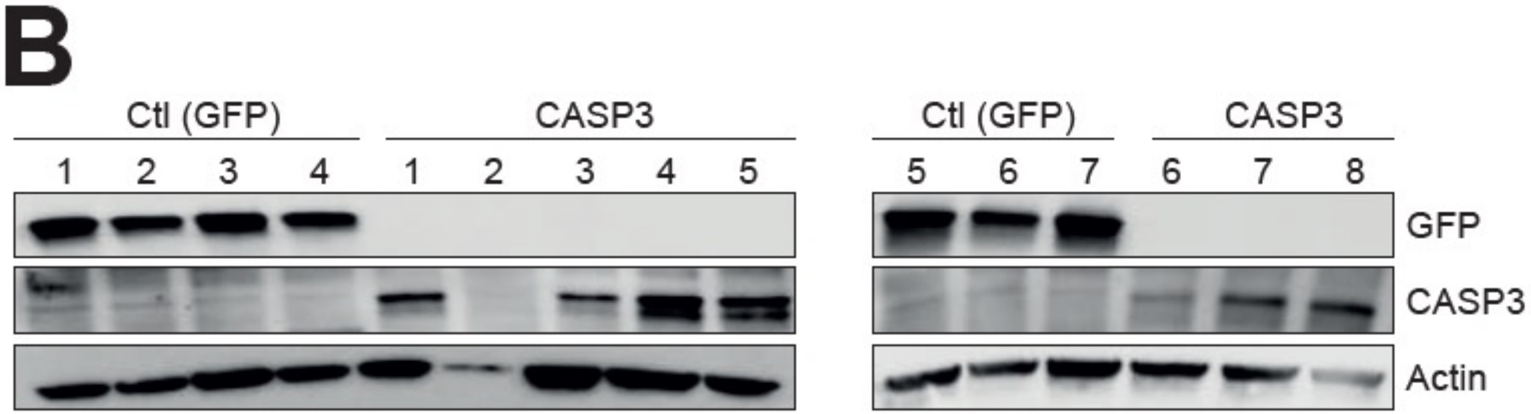

Figure 4B *Raw data*

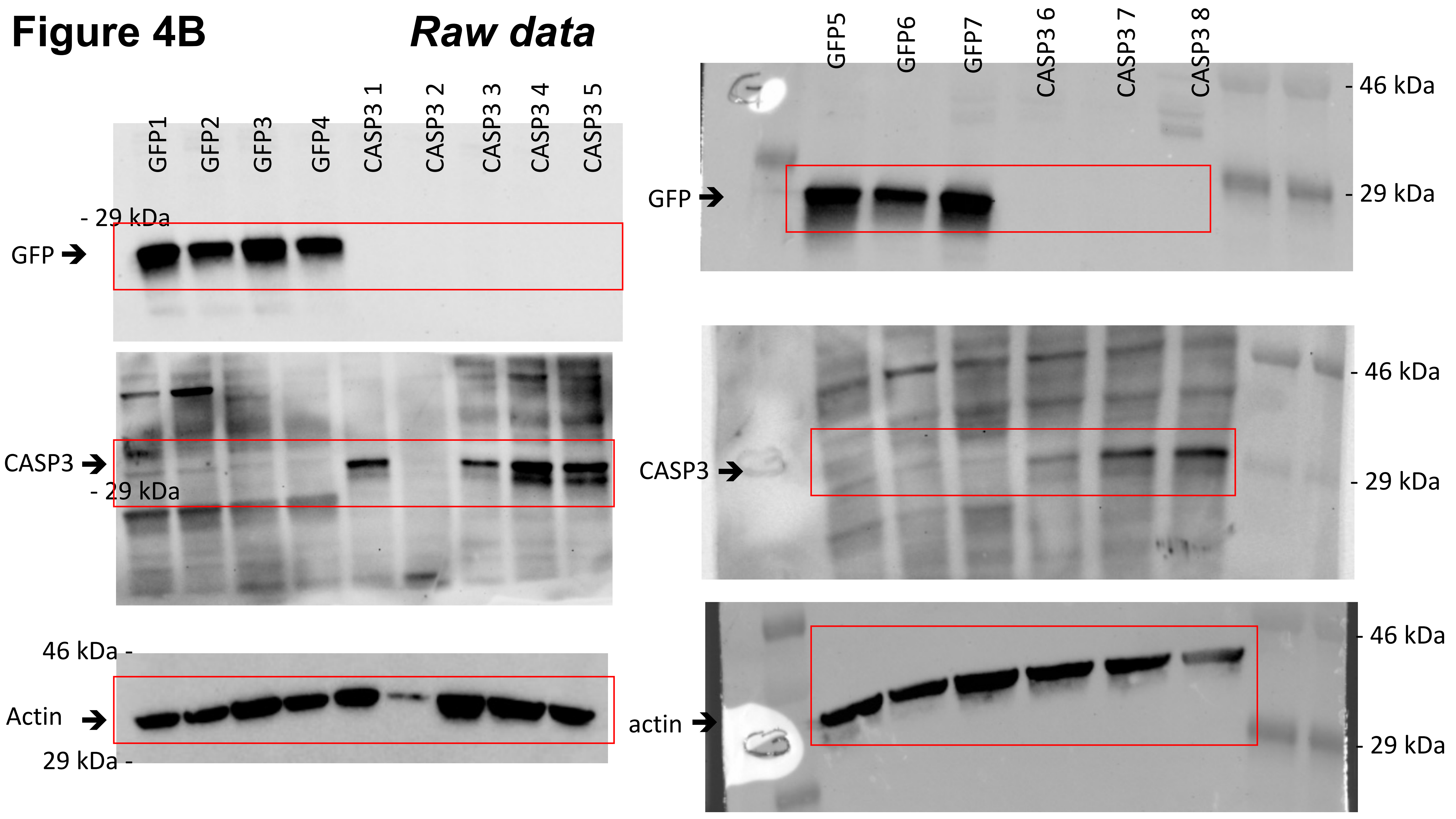

Figure 5B *Article version*

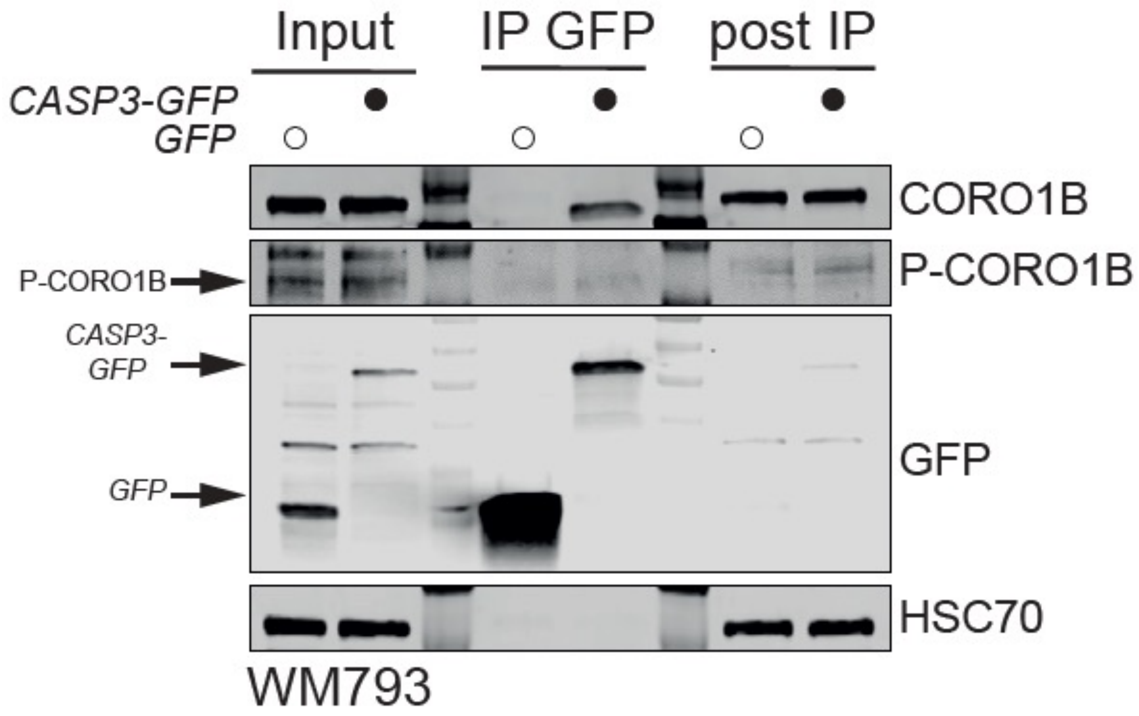

Figure 5B *Raw data*

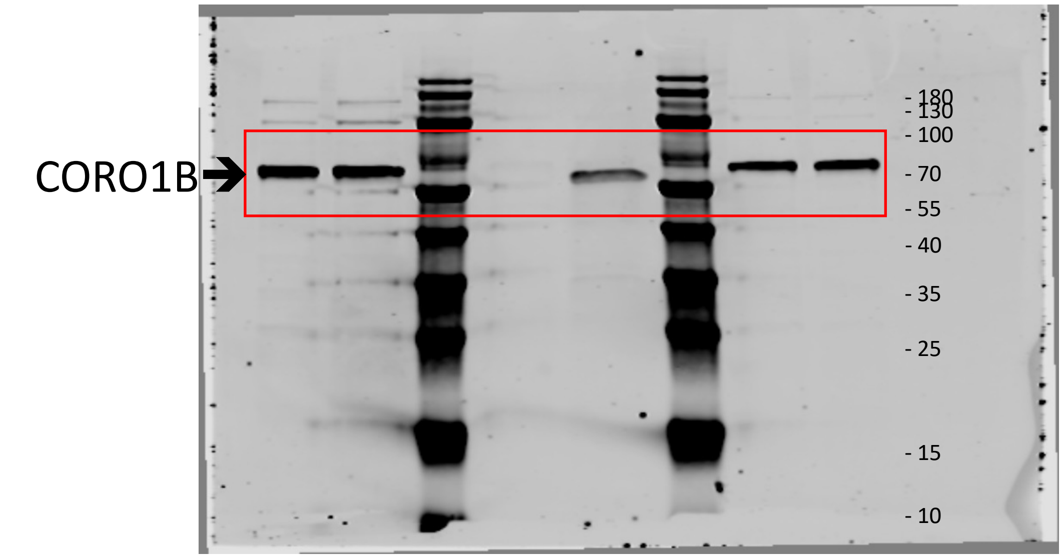

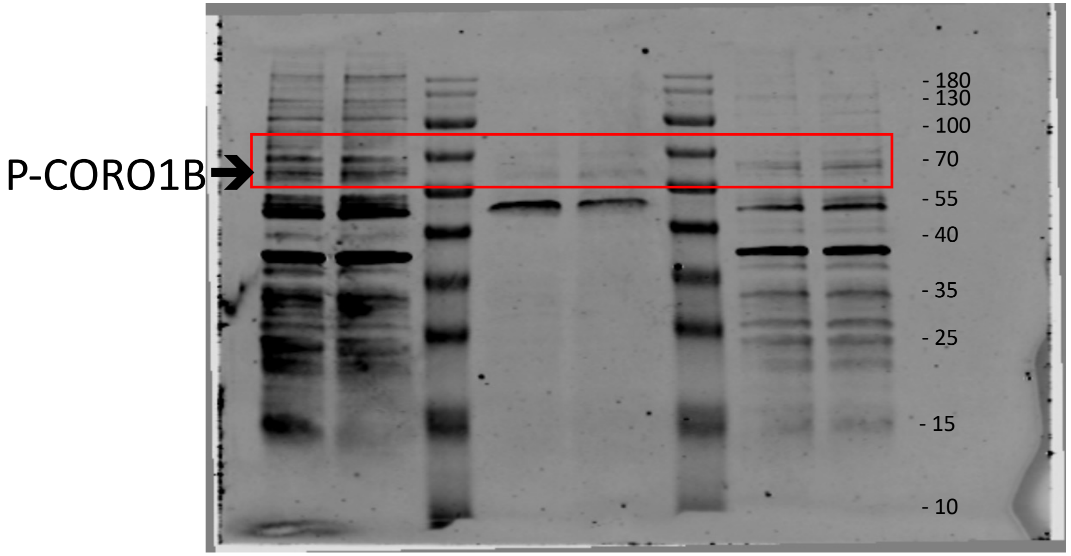

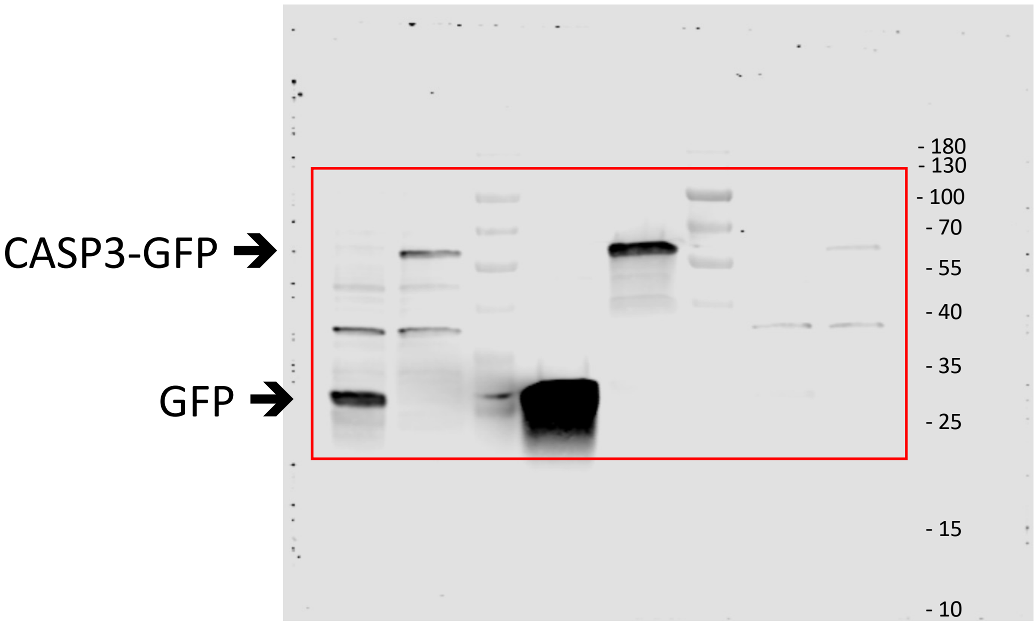

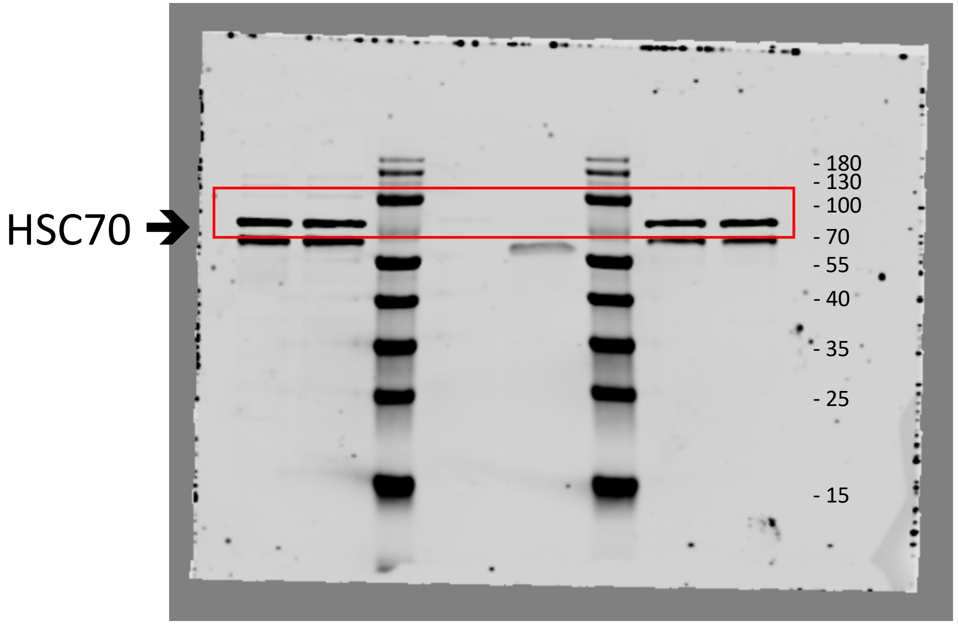

Figure 5H *Article version*

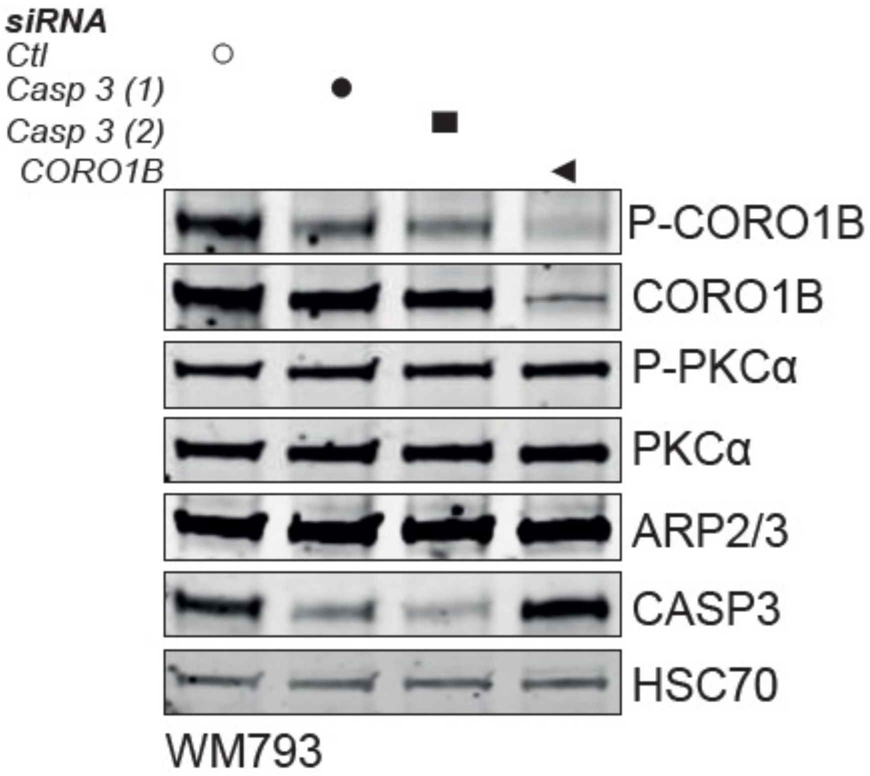

Figure 5H

Raw data

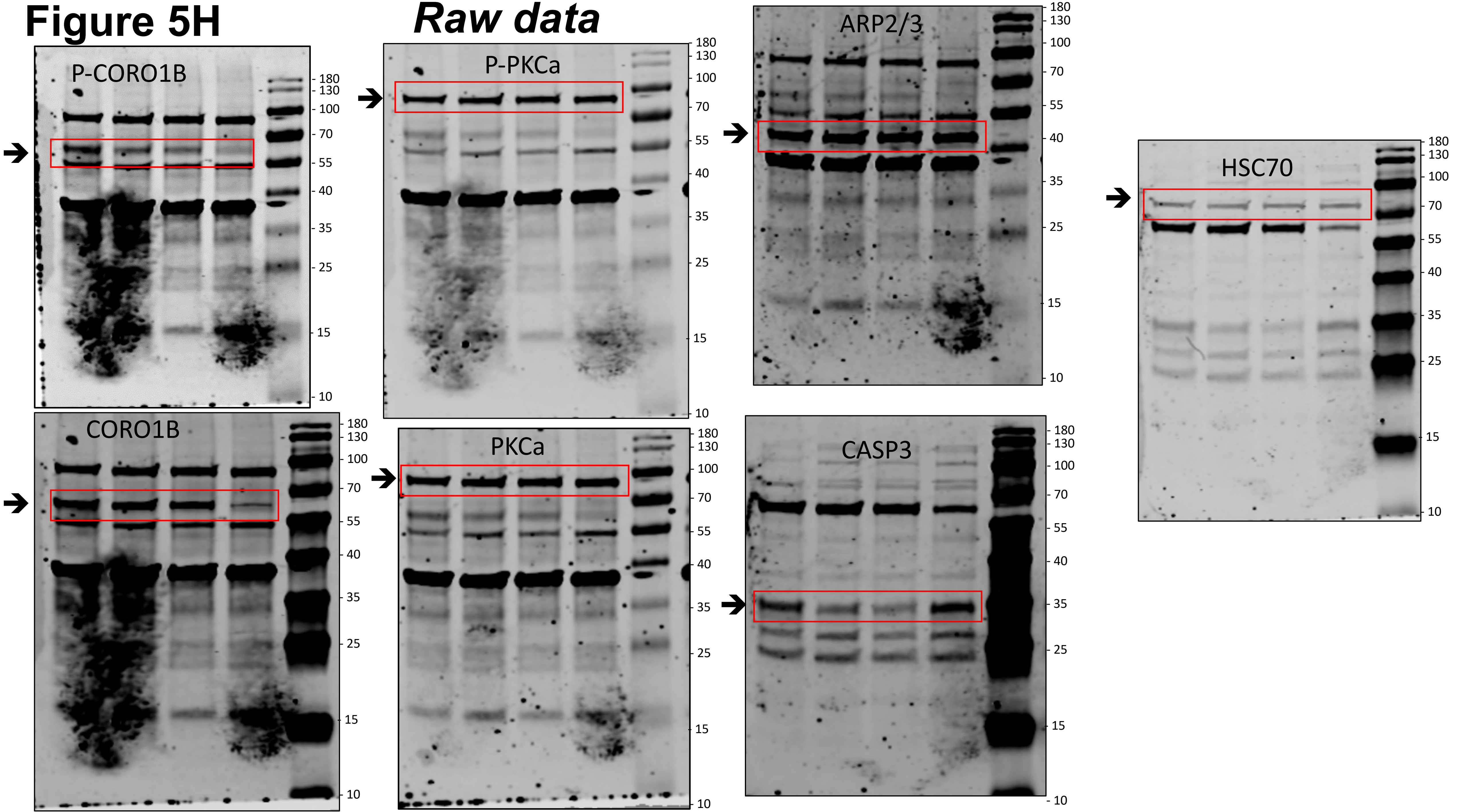

Figure 5I ***Article version***

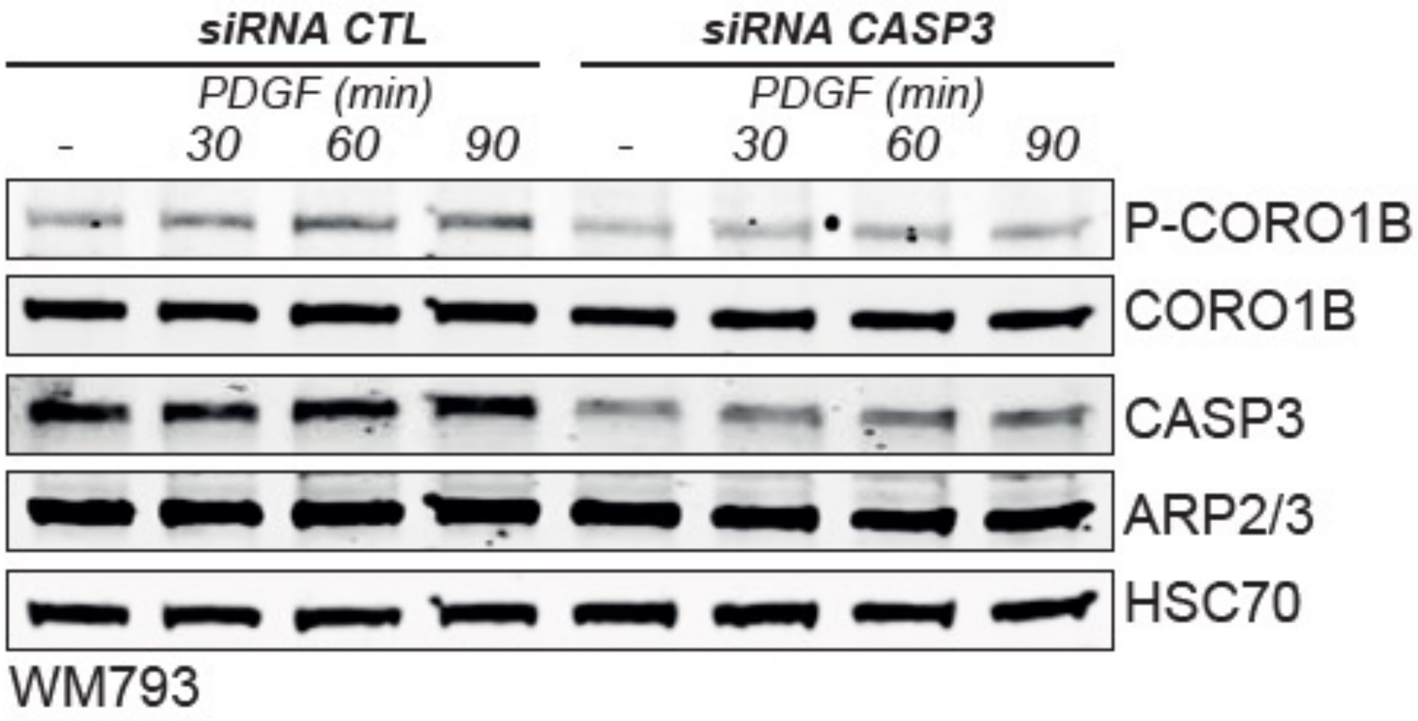

Figure 5I *Raw data*

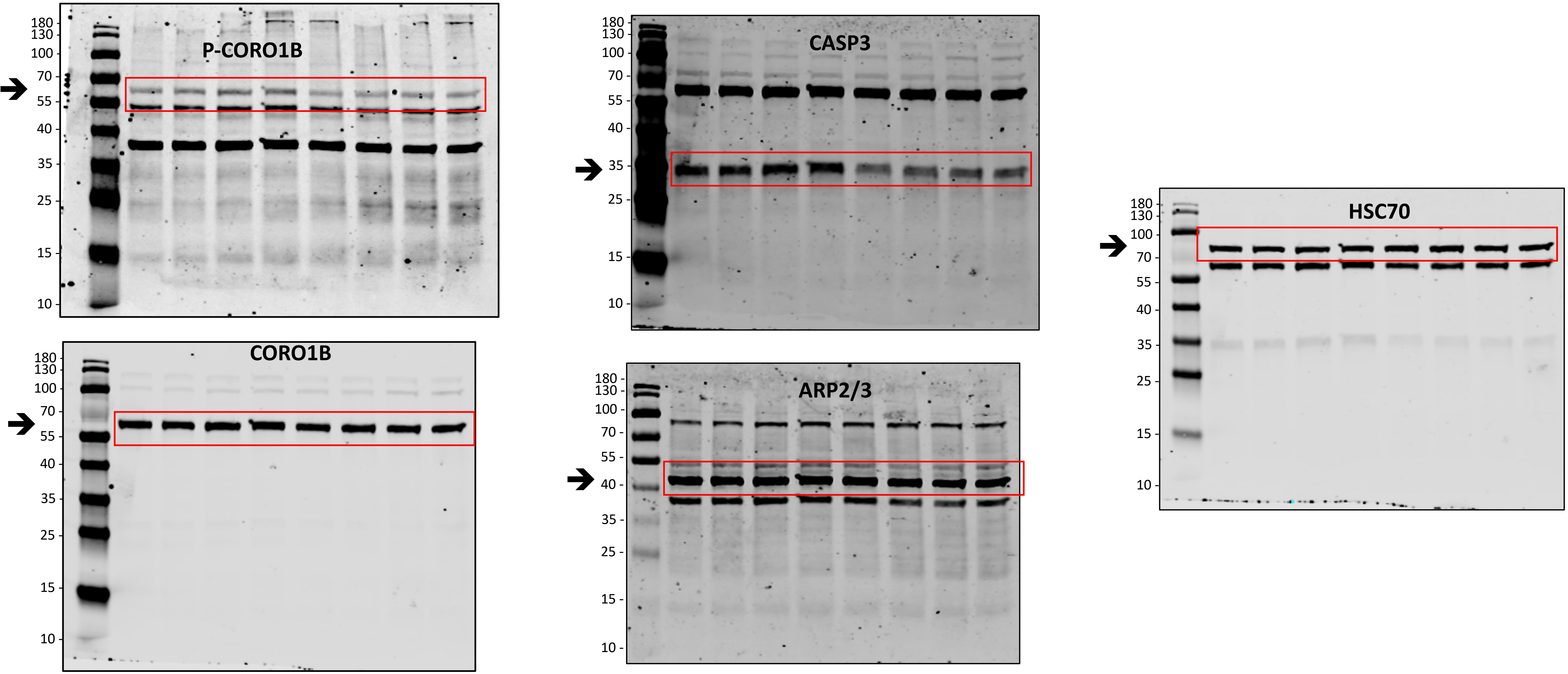

Figure 6D ***Article version***

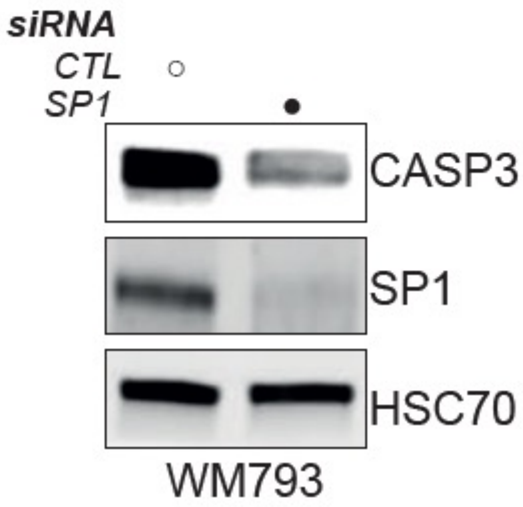

Figure 6D *Raw data*

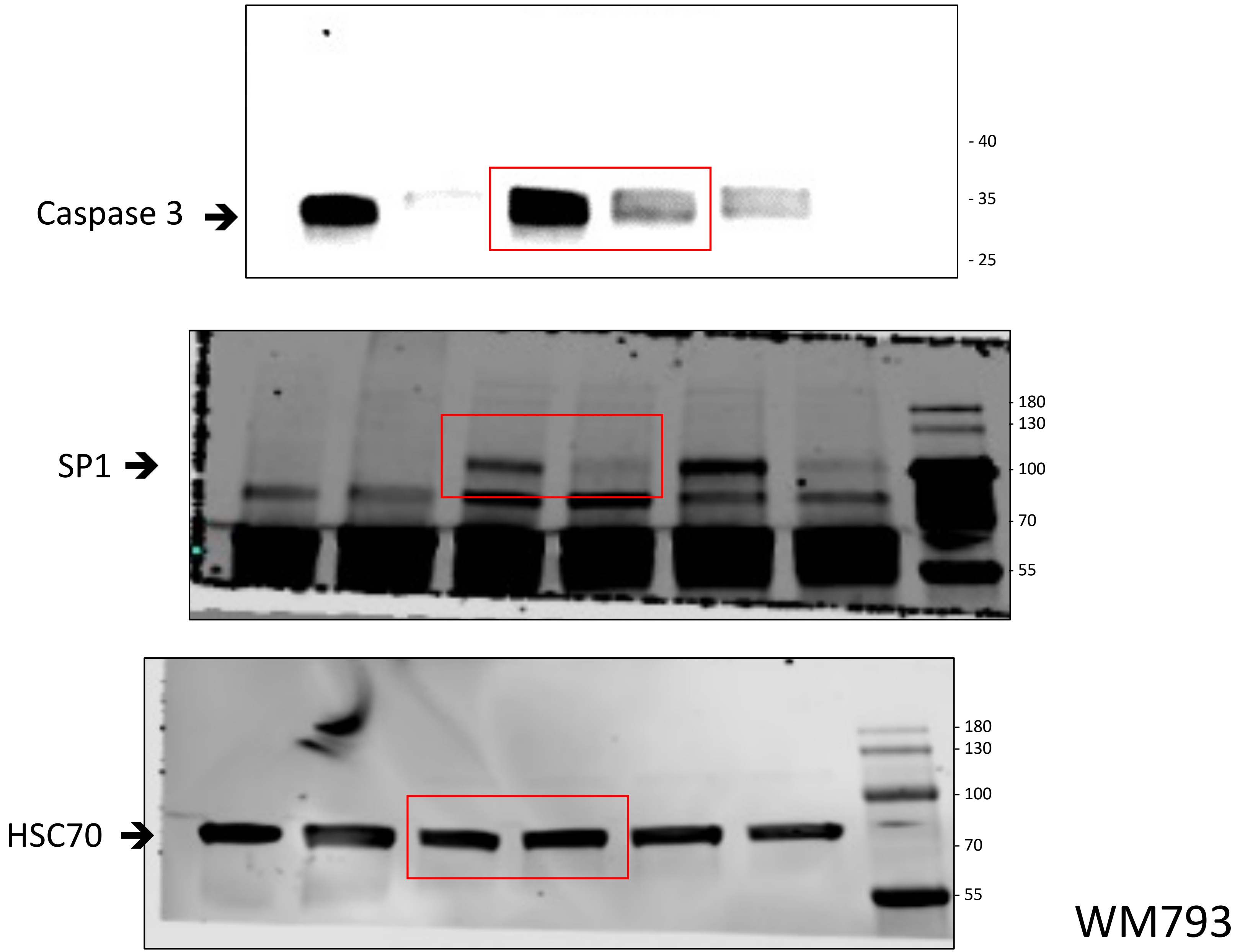

Figure 6E *Article version*

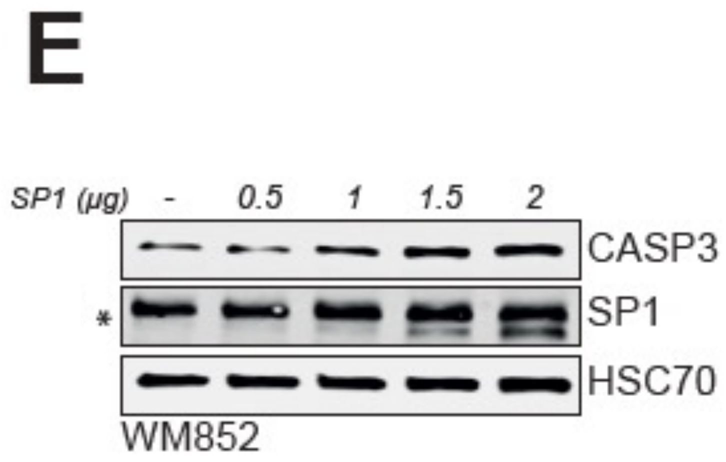

Figure 6E *Raw data*

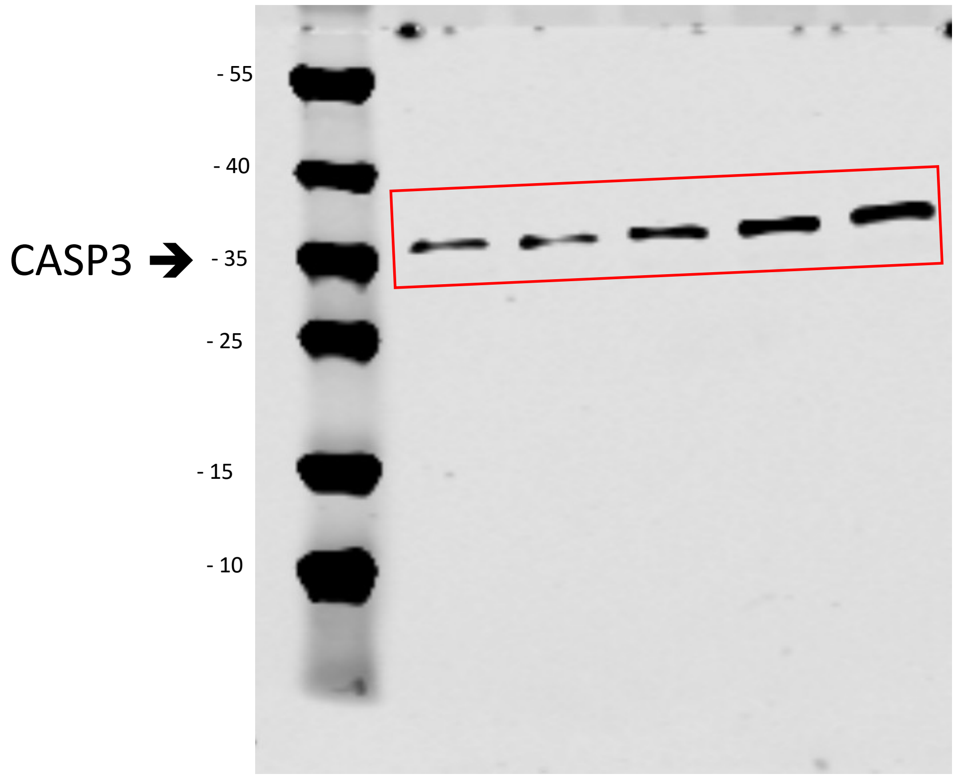

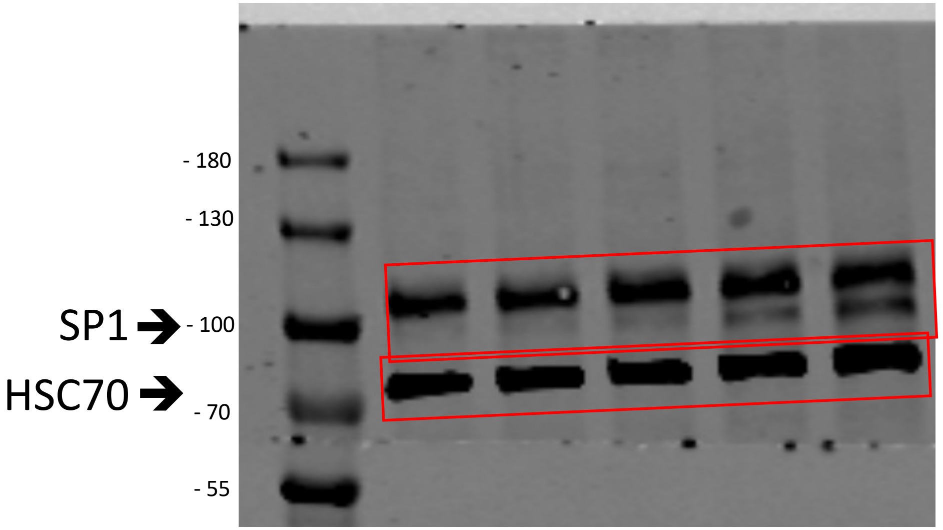

Figure 6I ***Article version***

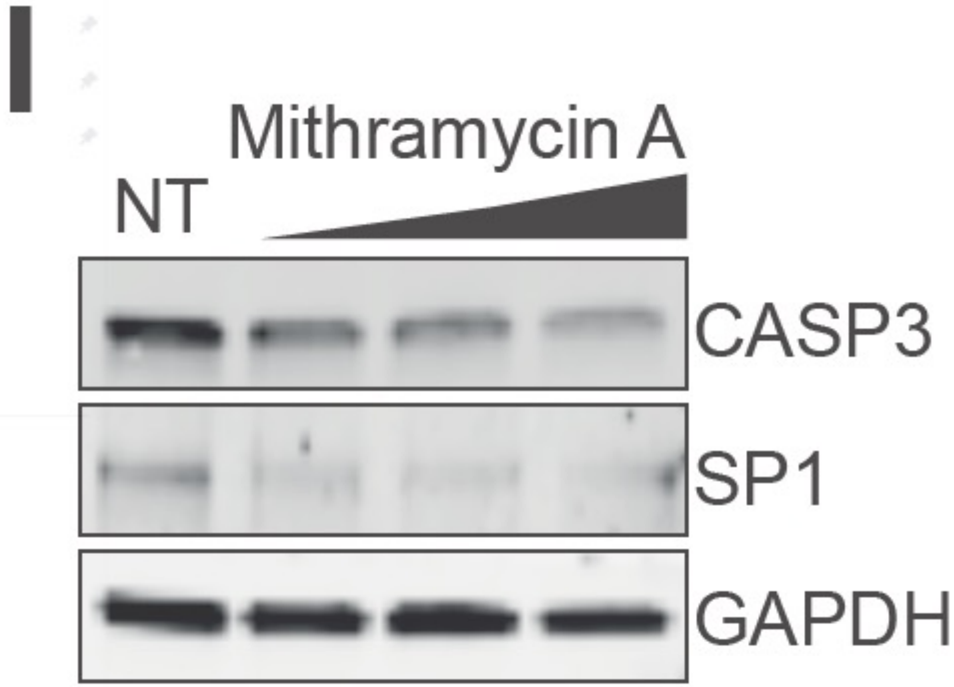

Figure 6I *Raw data*

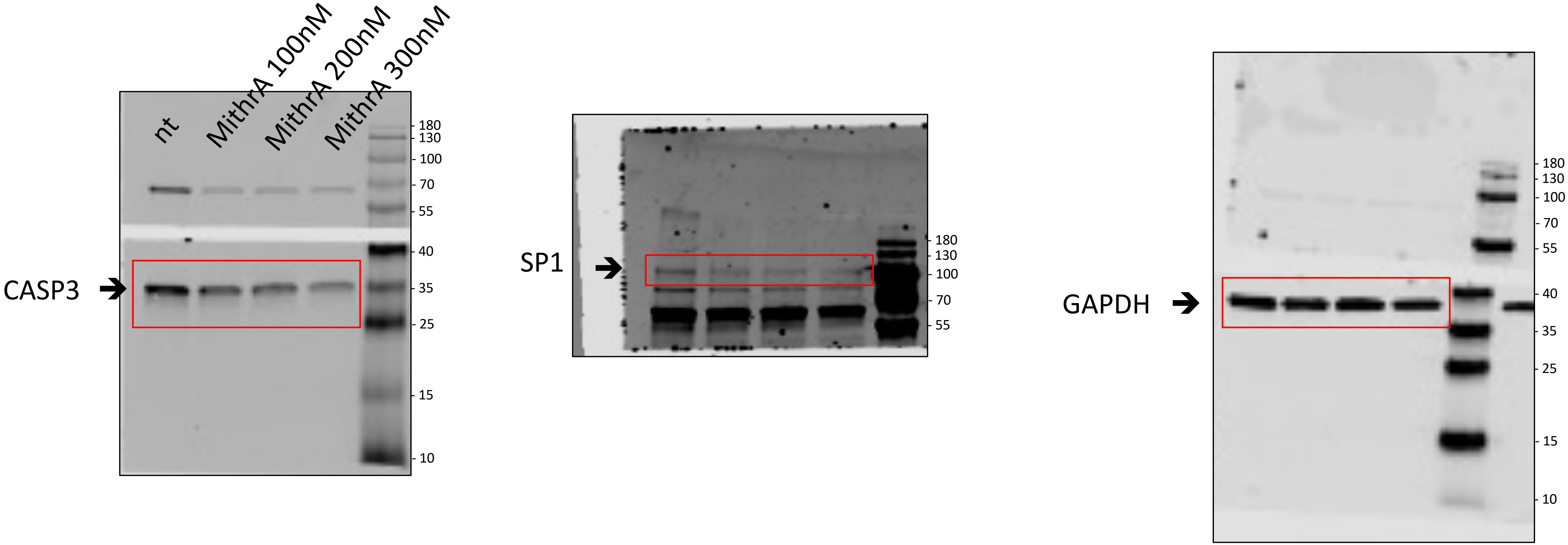

WM793

Figure S1A ***Article version***

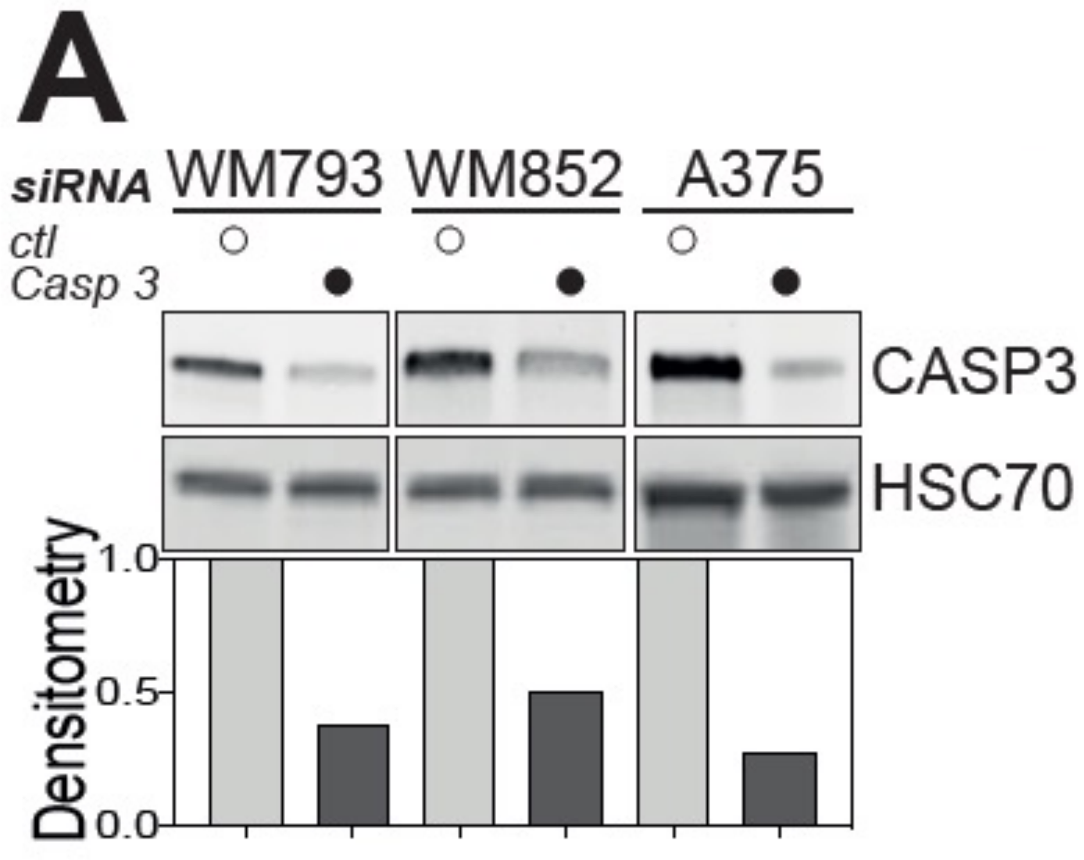

Figure S1A *Raw data*

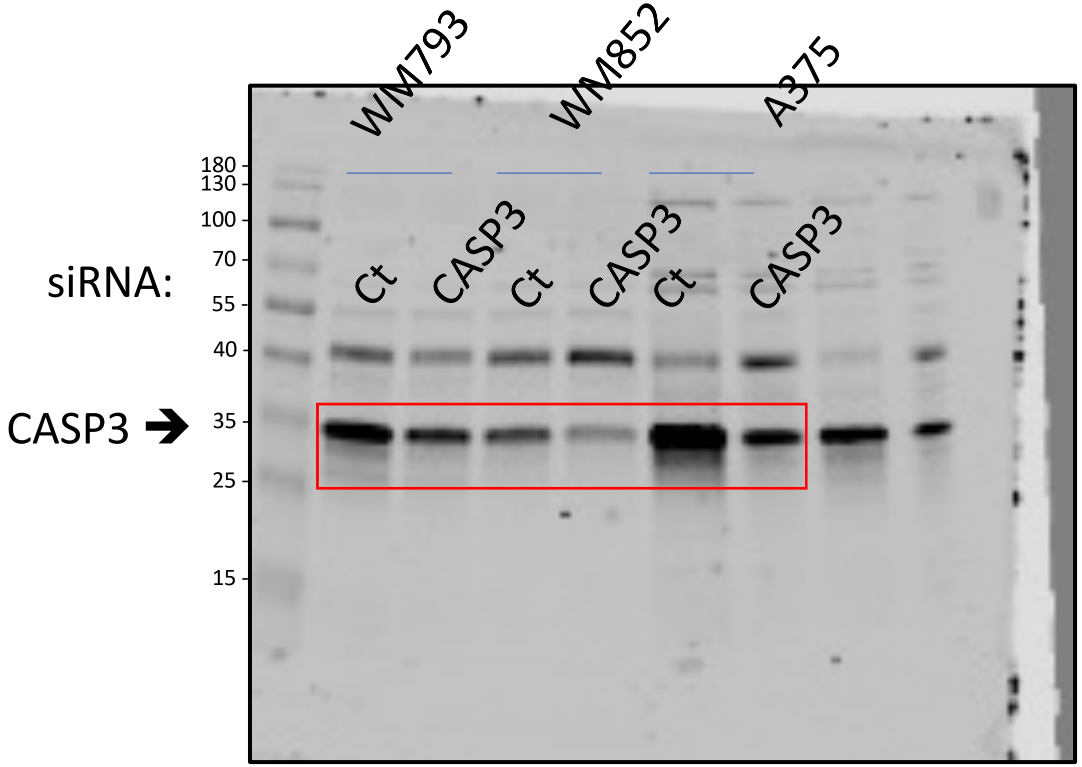

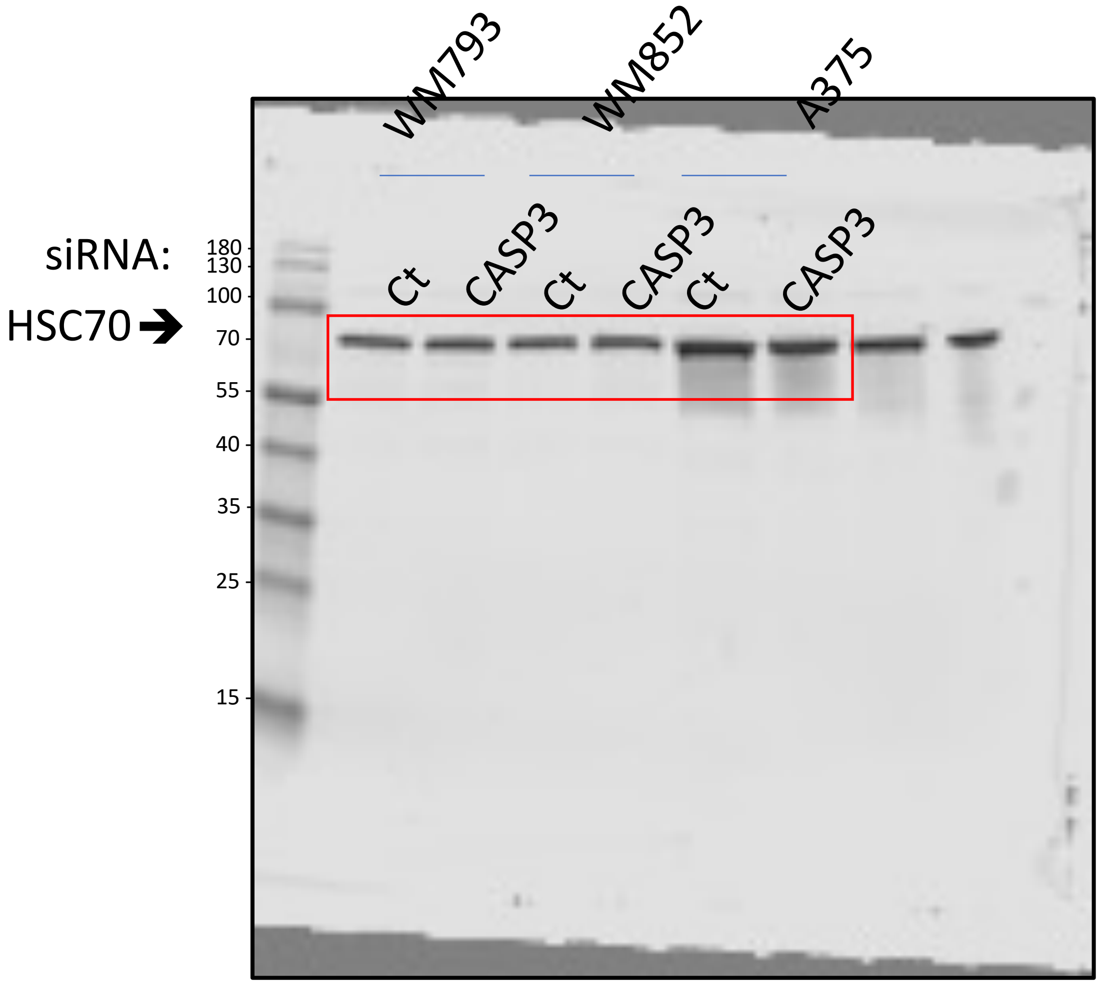

Figure S1F ***Article version***

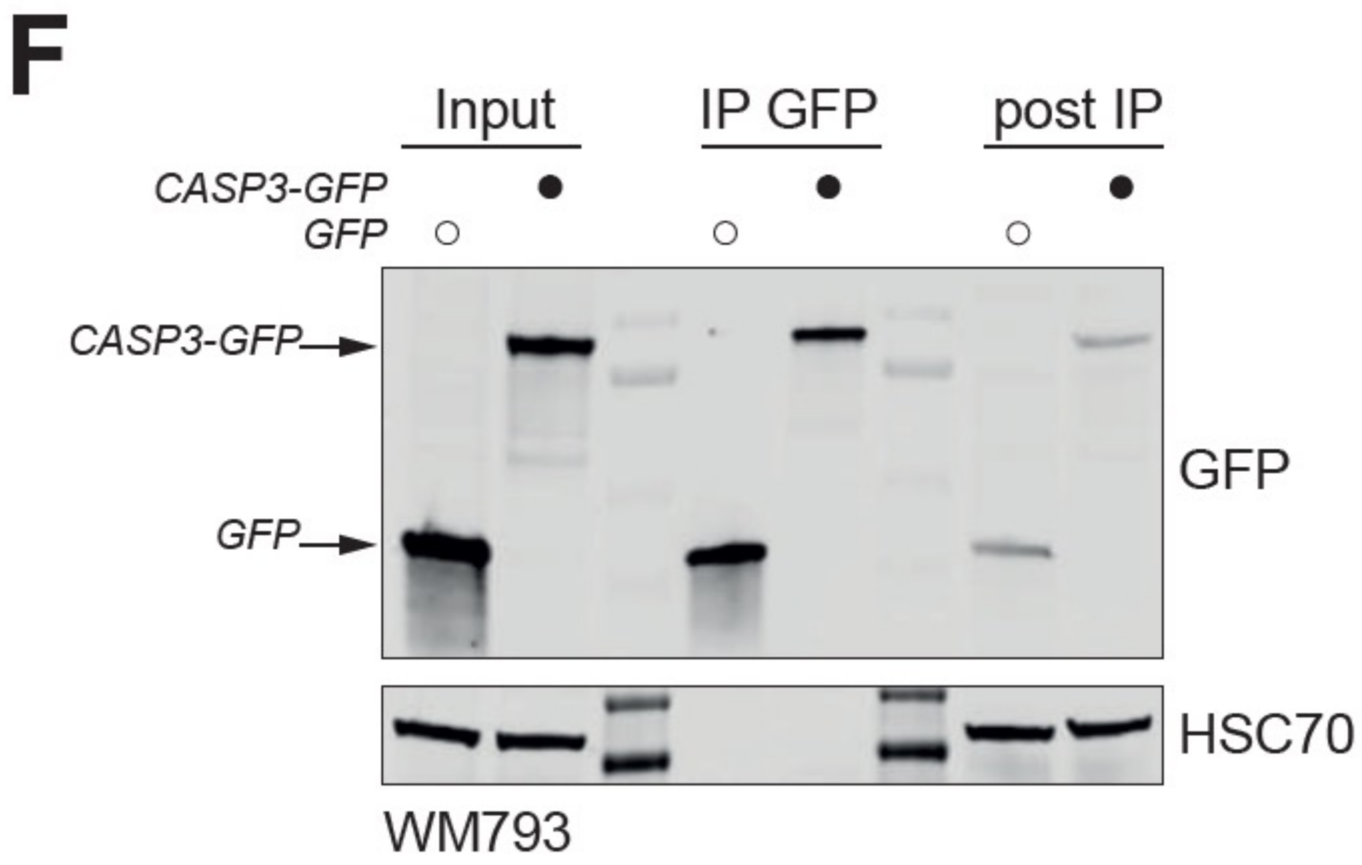

Figure S1F *Raw data*

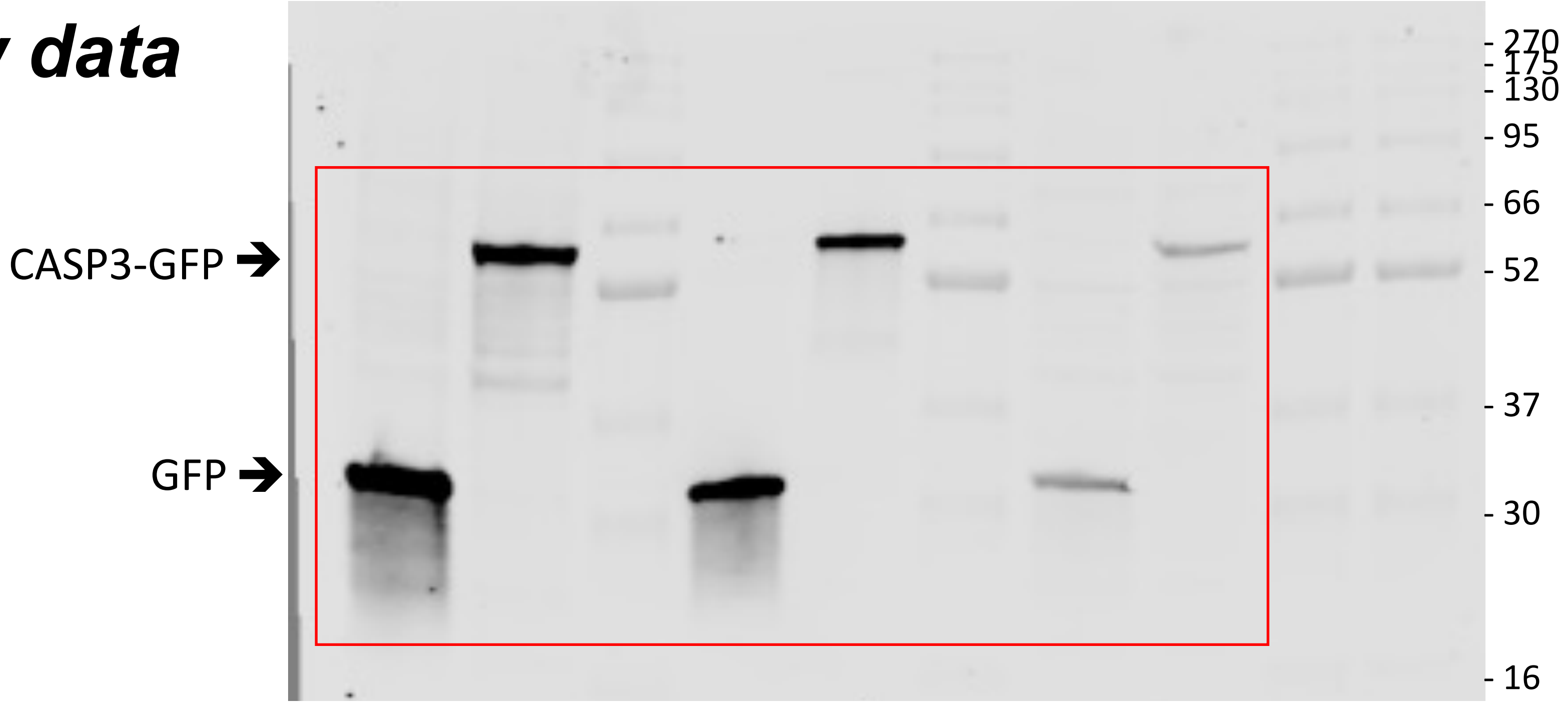

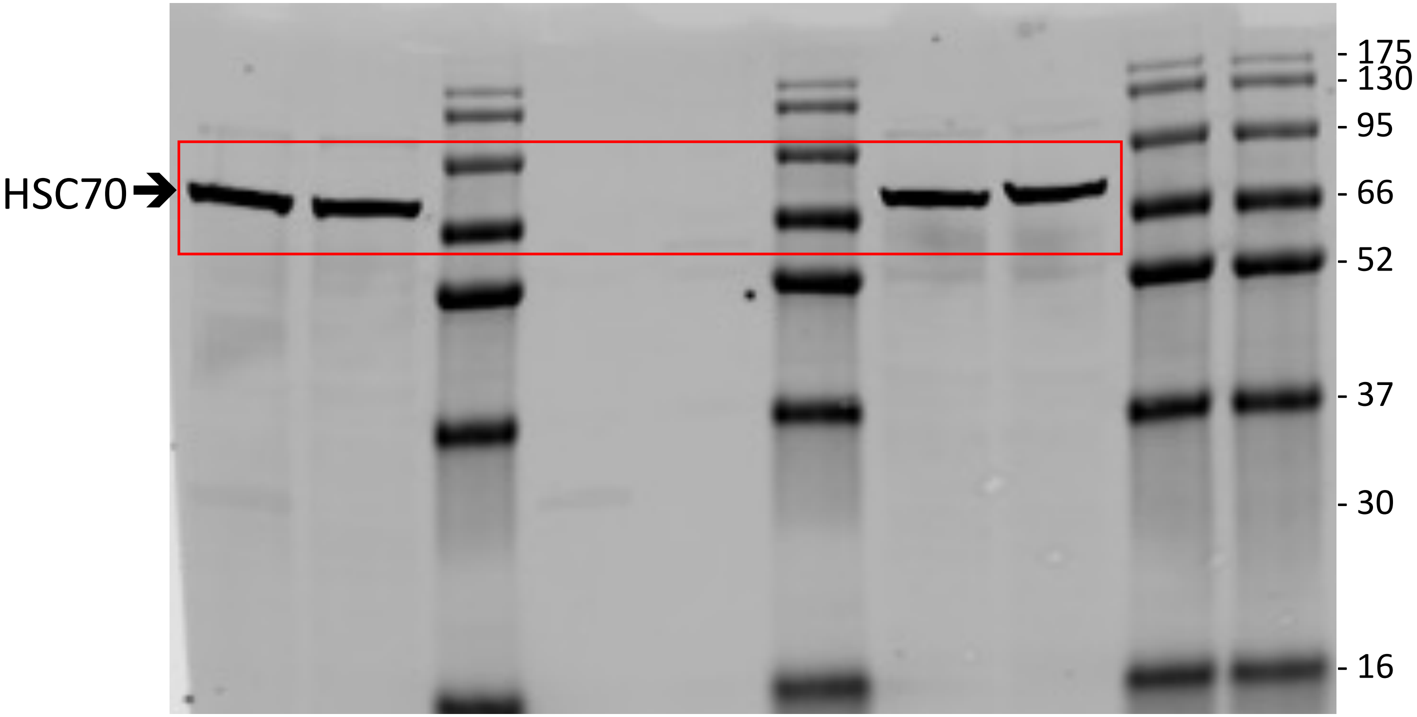

Figure S1G *Article version*

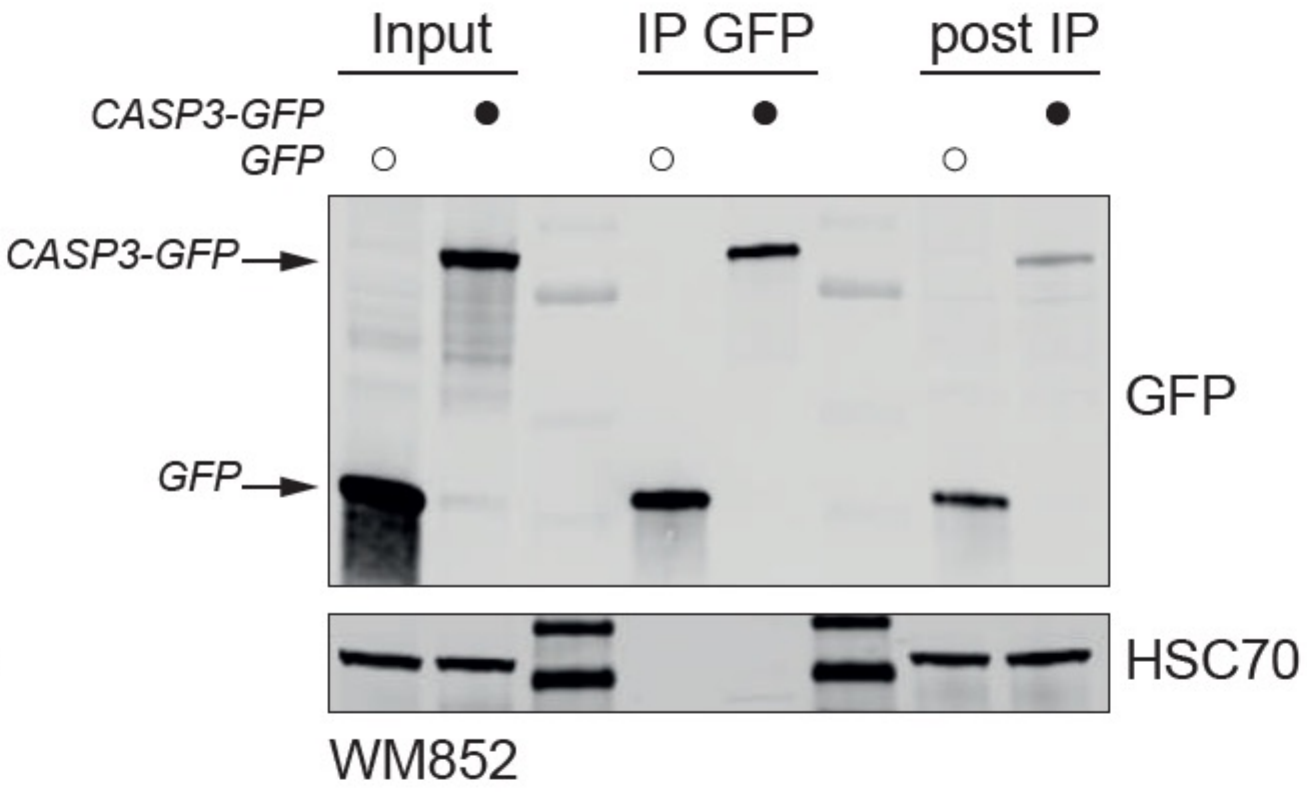

Figure S1G *Raw data*

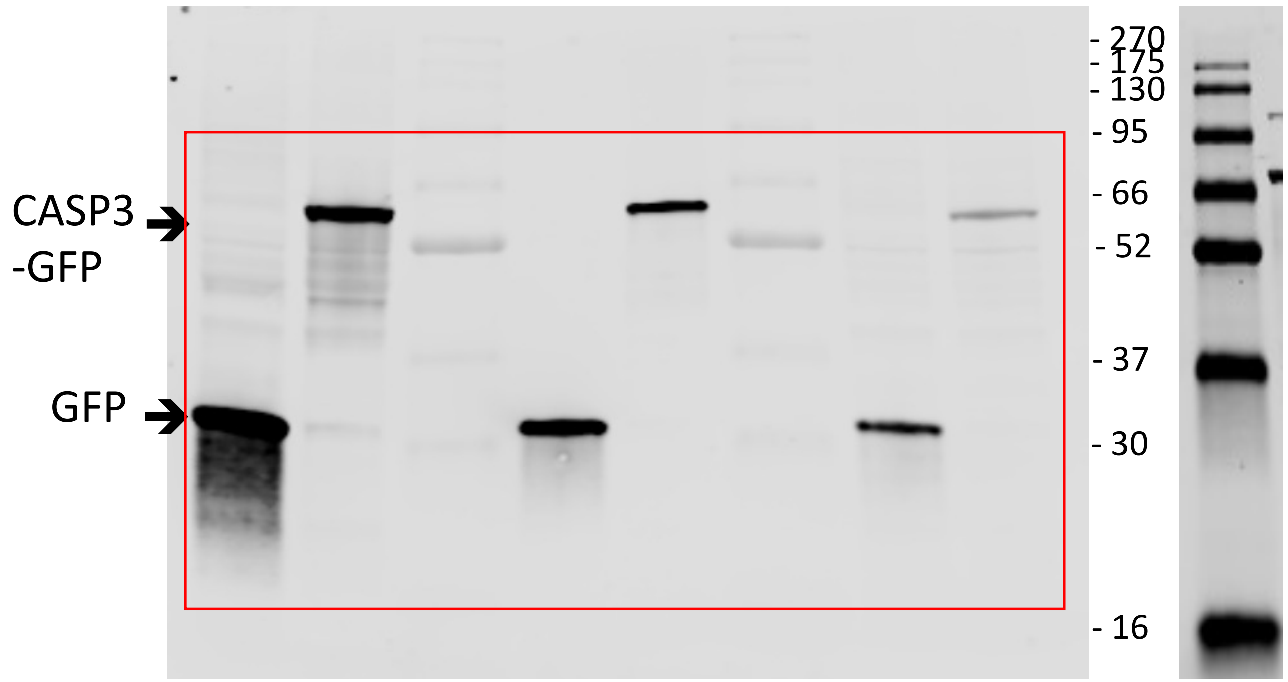

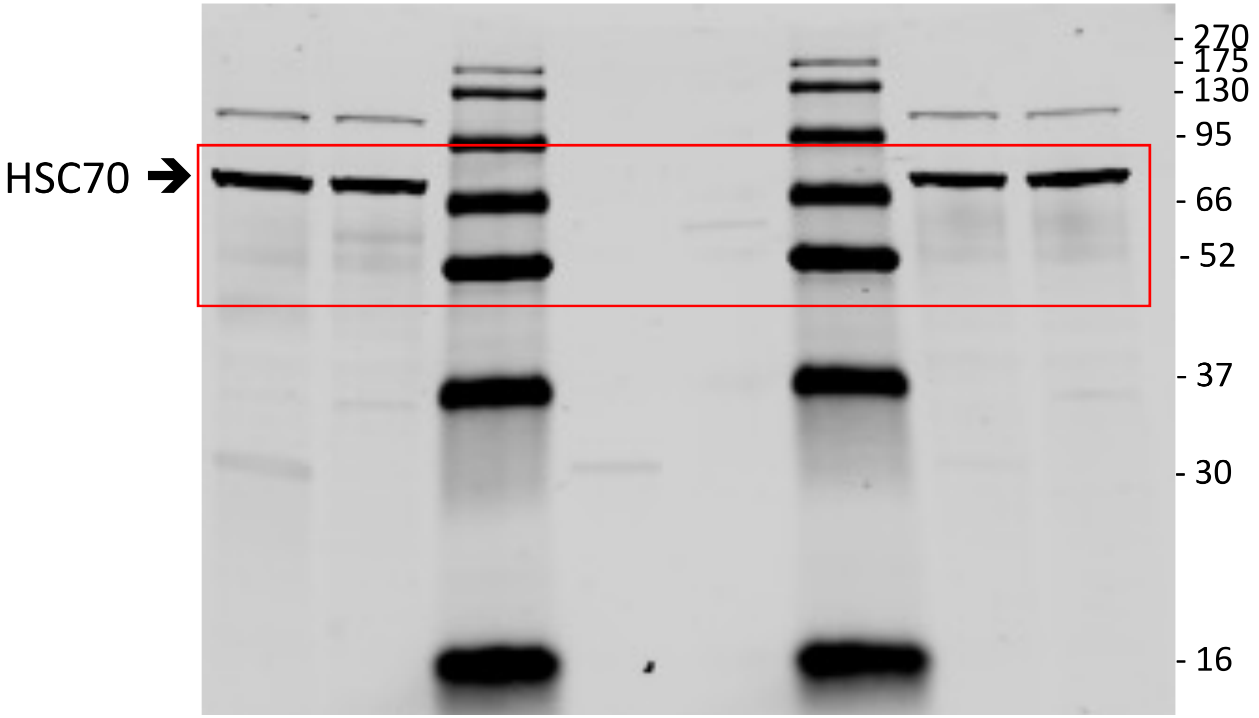

Figure S2A *Article version*

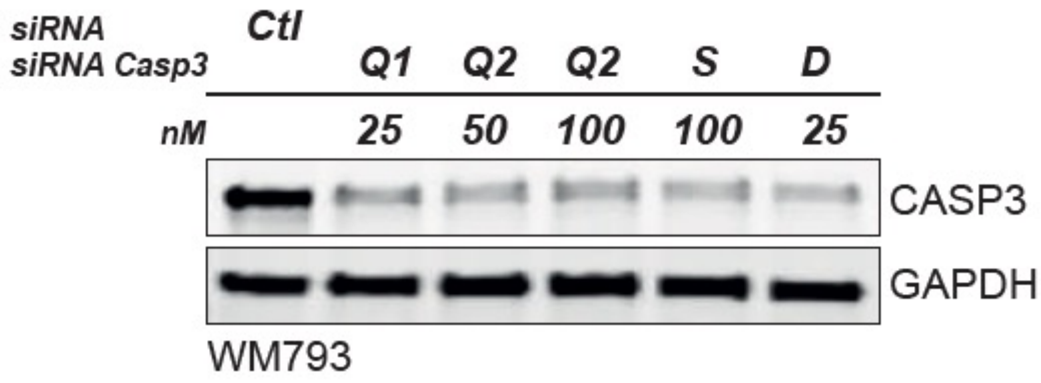

Figure S2A *Raw data*

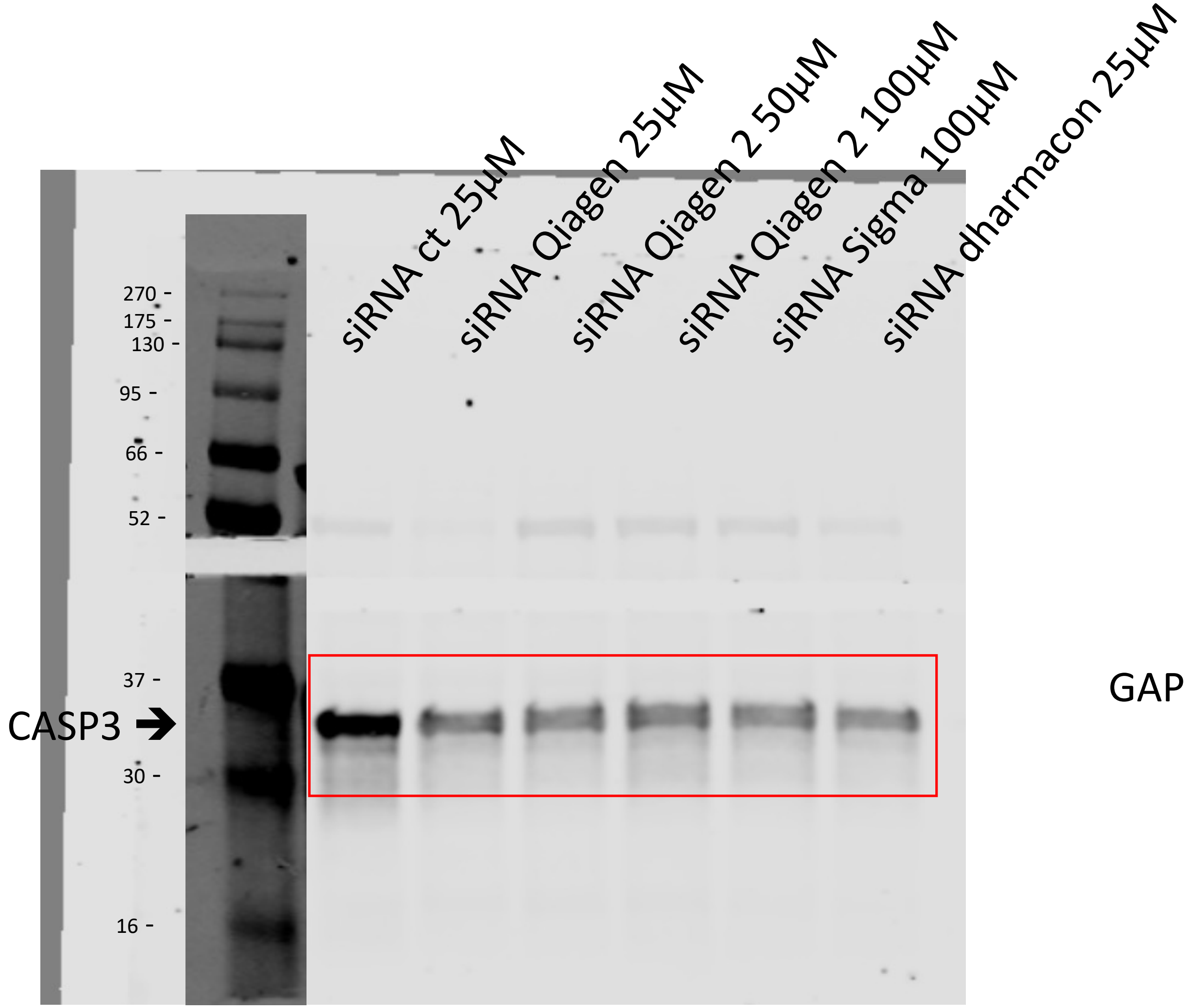

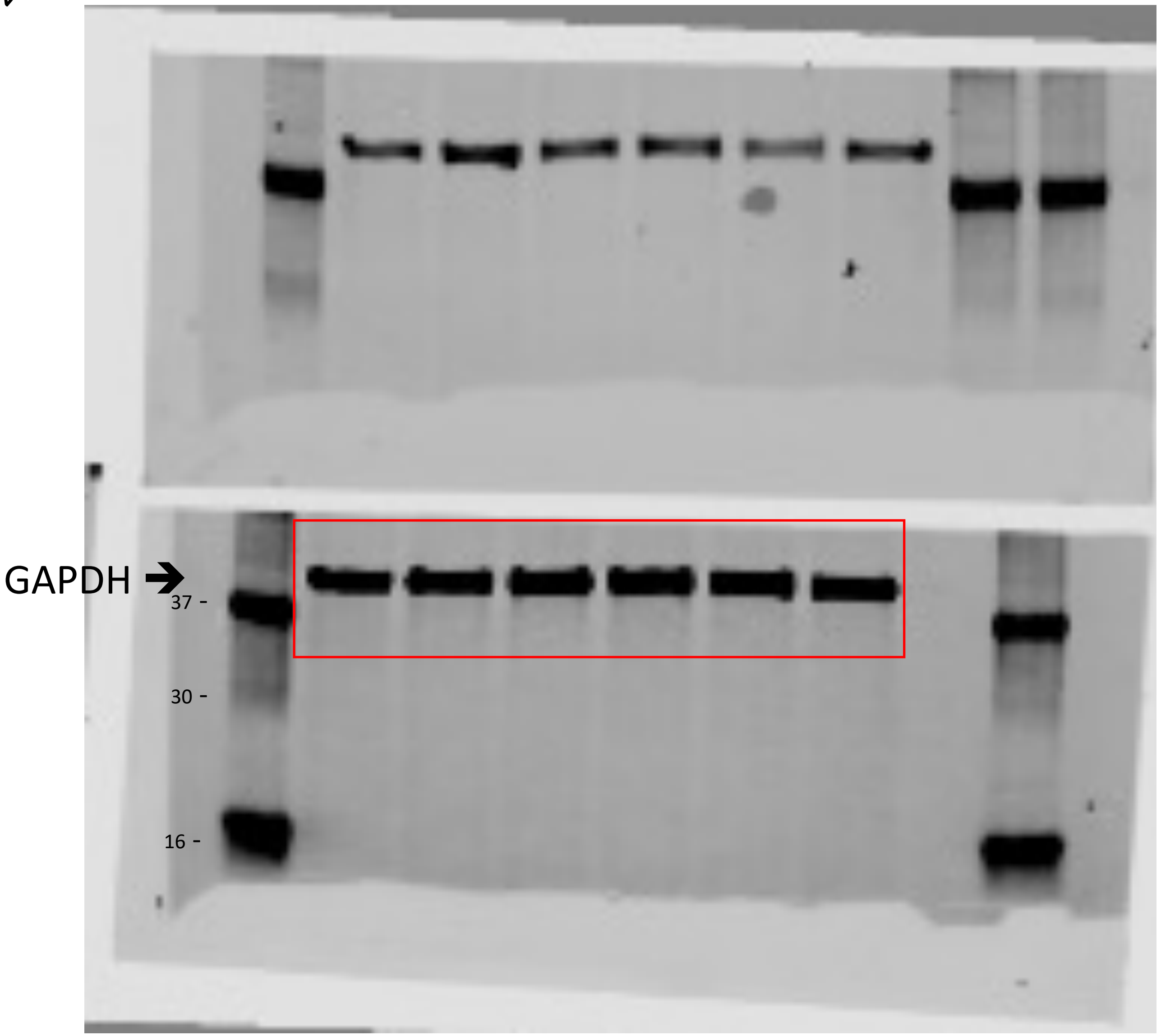

WM793

Figure S2C ***Article version***

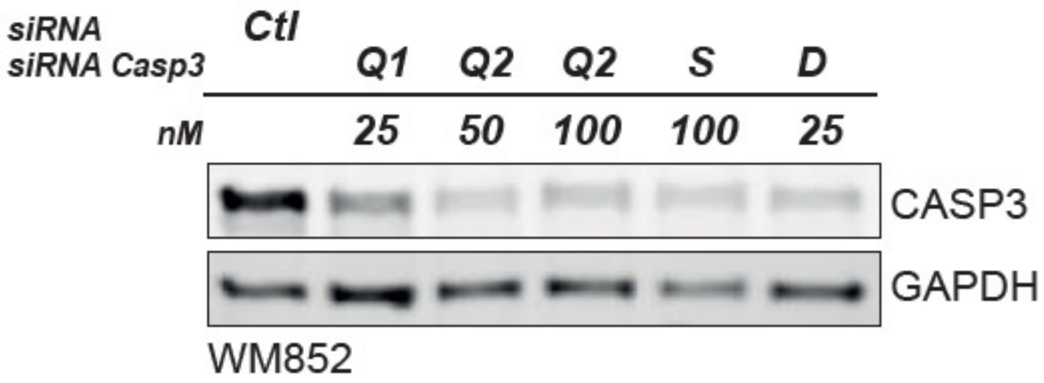

Figure S2C *Raw data*

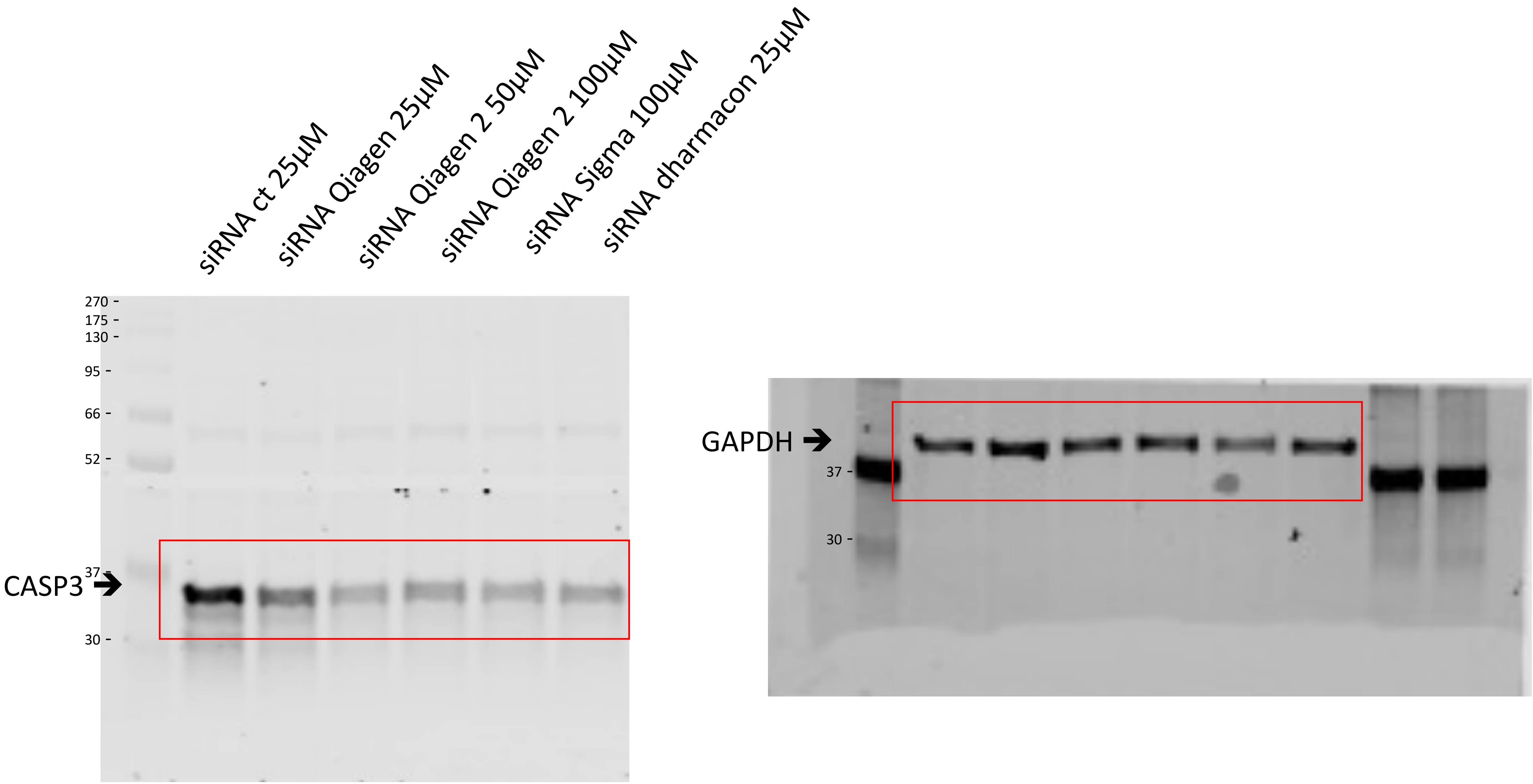

WM852

Figure S2E *Article version*

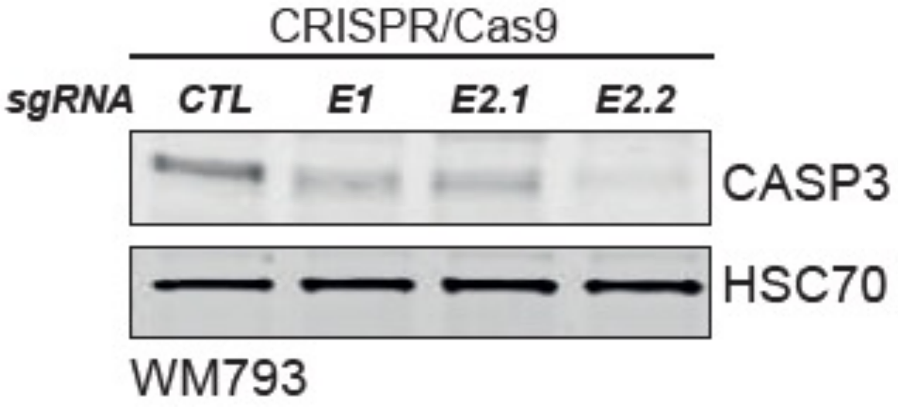

Figure S2E *Raw data*

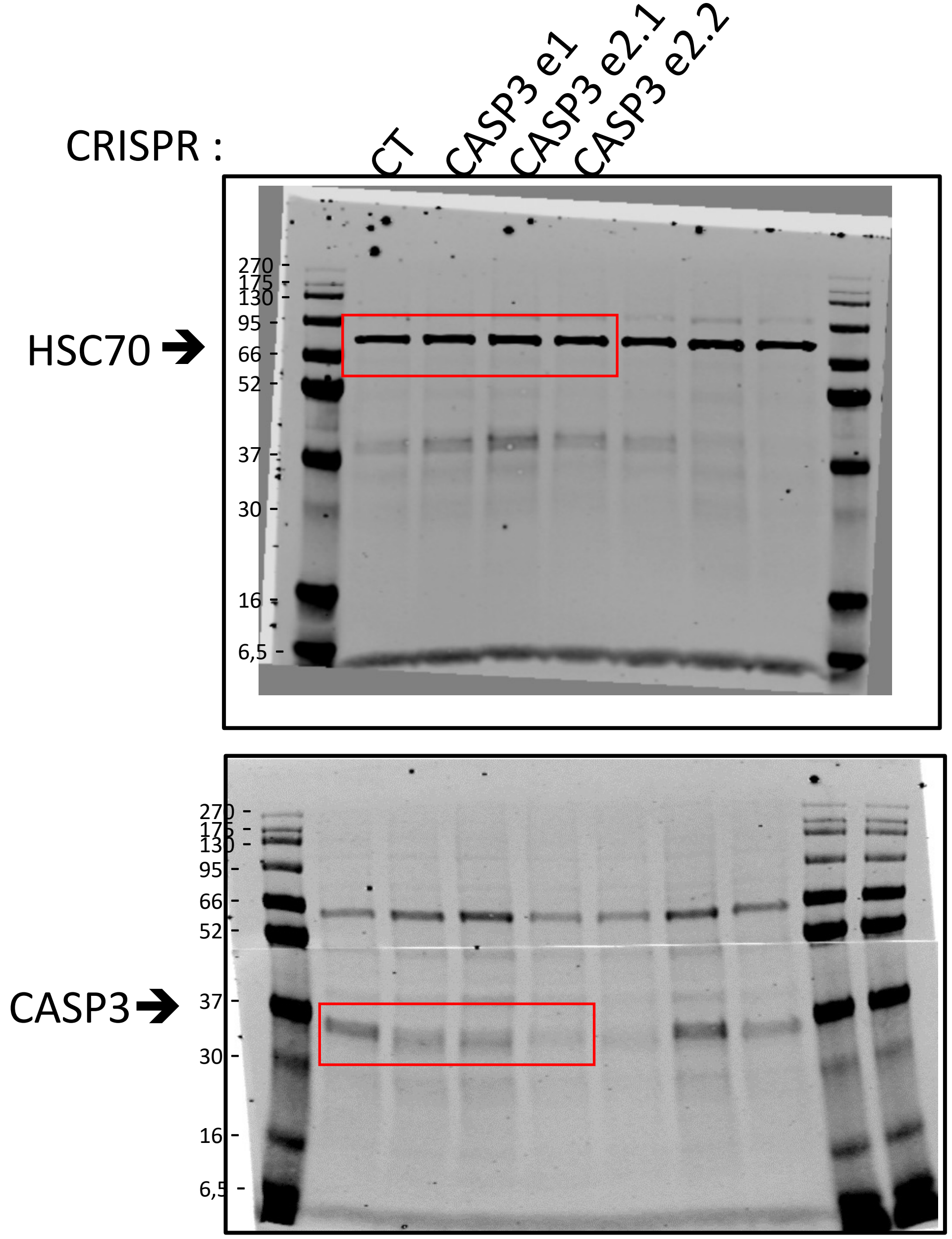

WM793

Figure S2G *Article version*

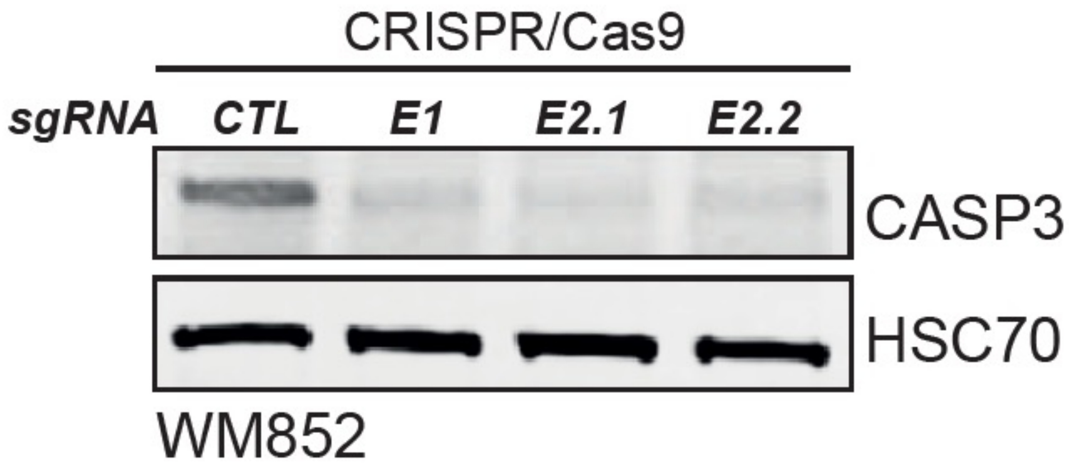

Figure S2G *Raw data*

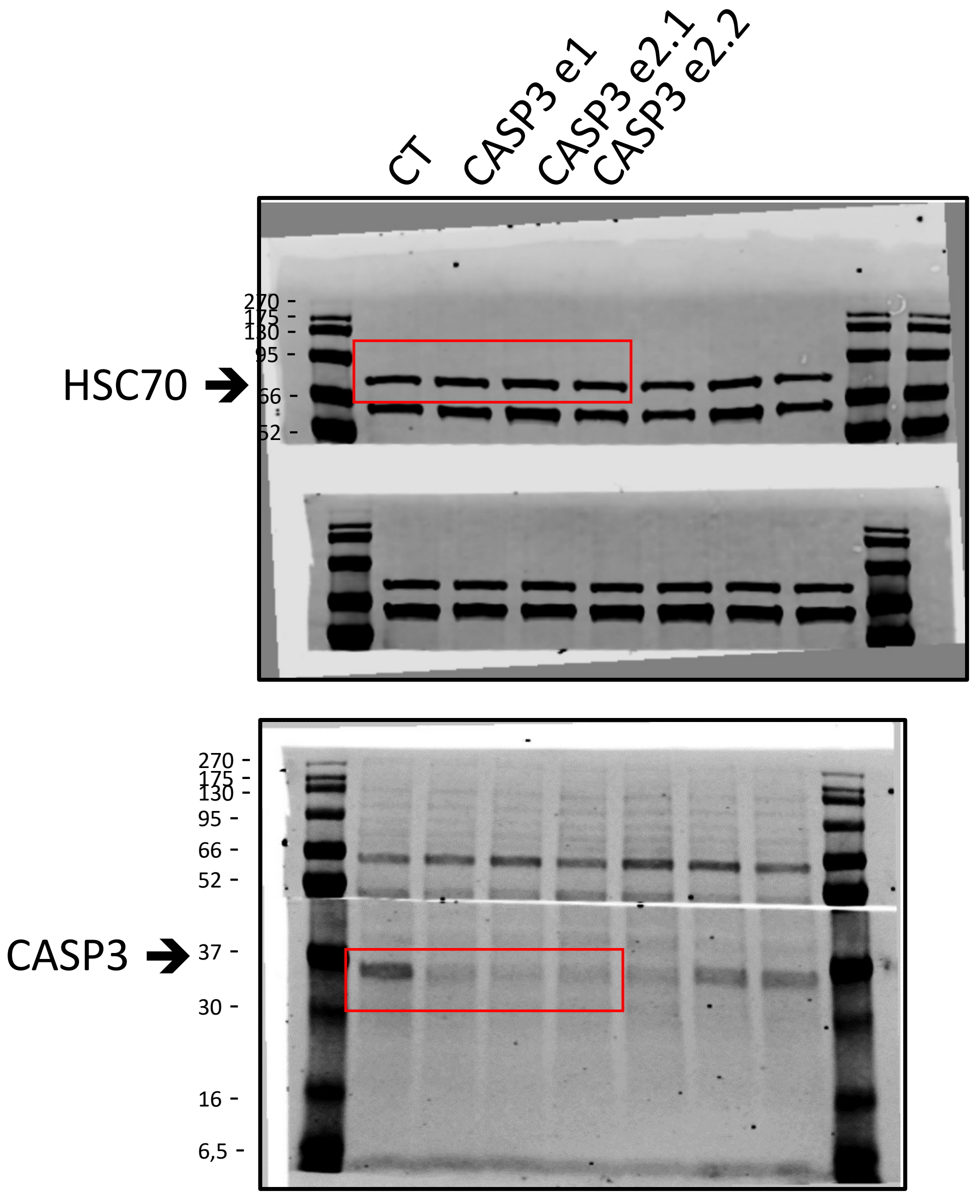

WM852

Figure S2I *Article version*

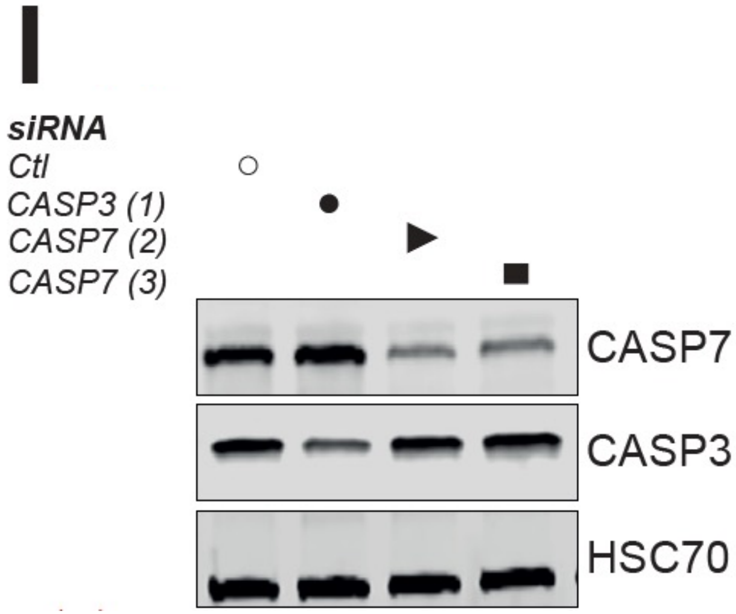

Figure S2I *Raw data*

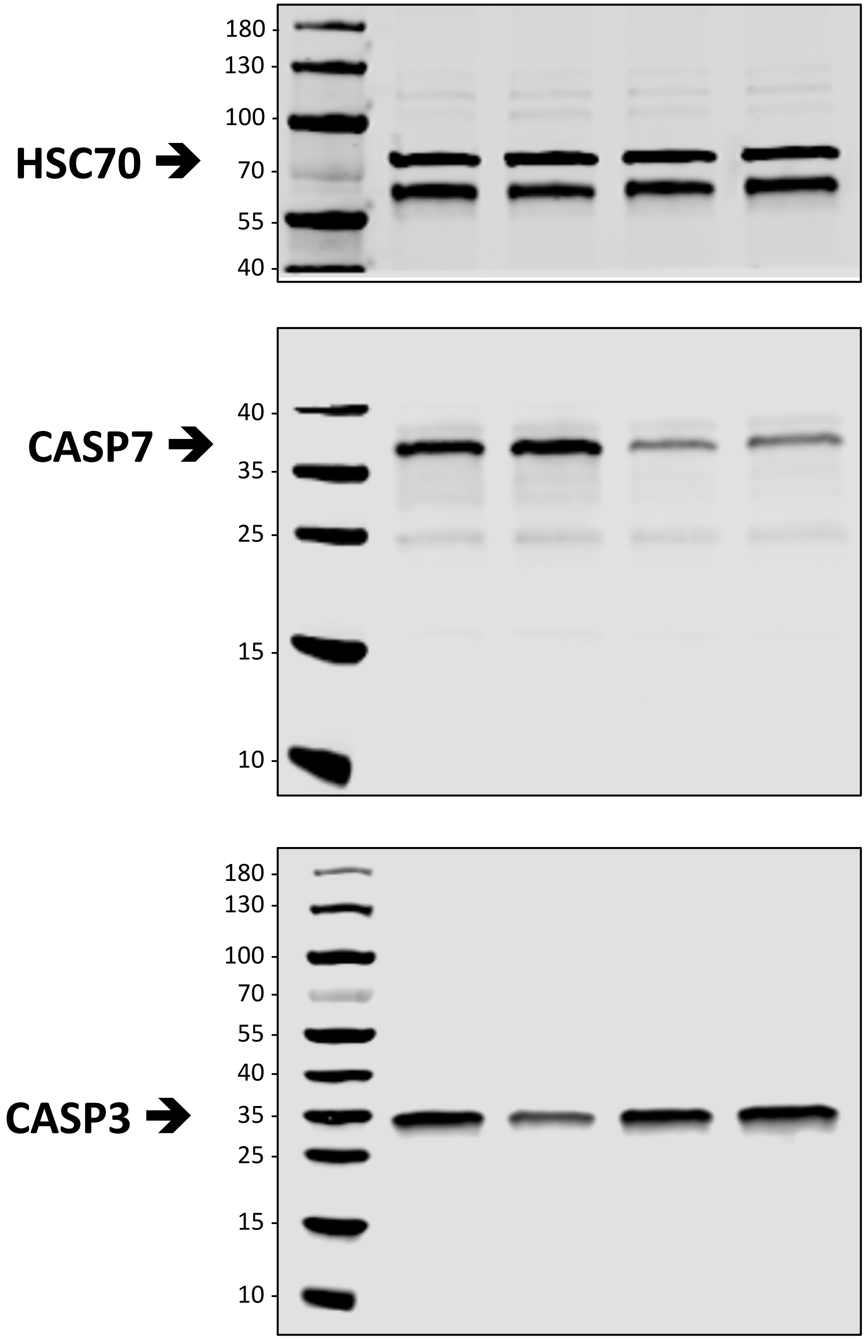

Figure S2P *Article version*

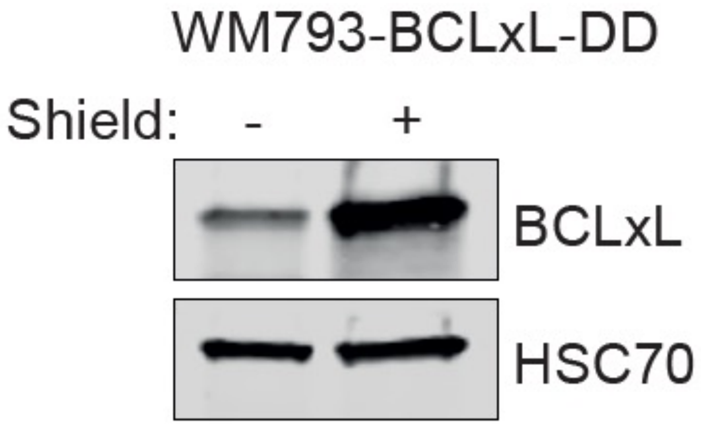

Figure S2P *Raw data*

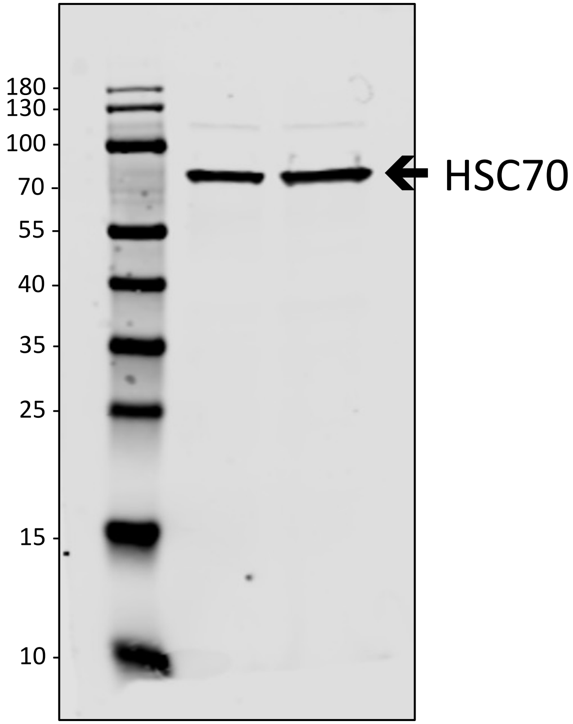

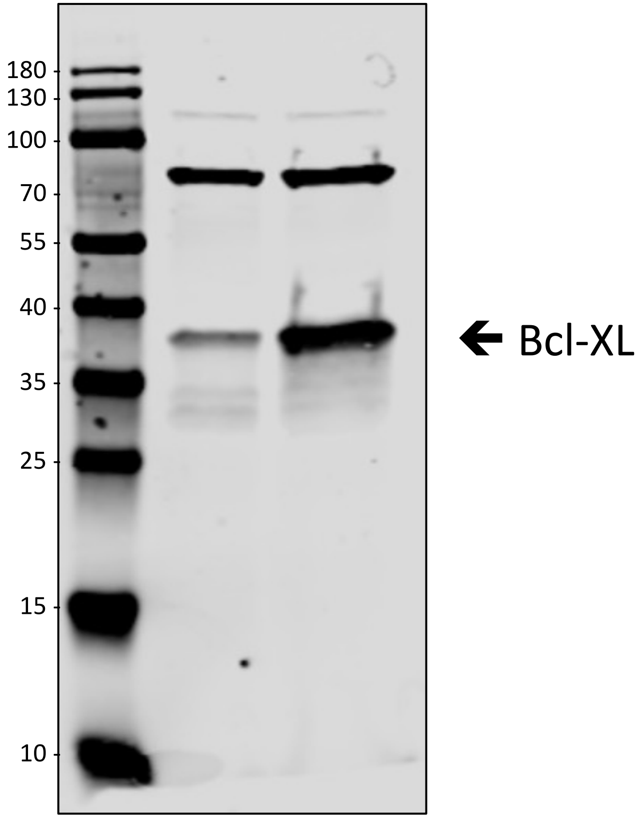

Figure S2R ***Article version***

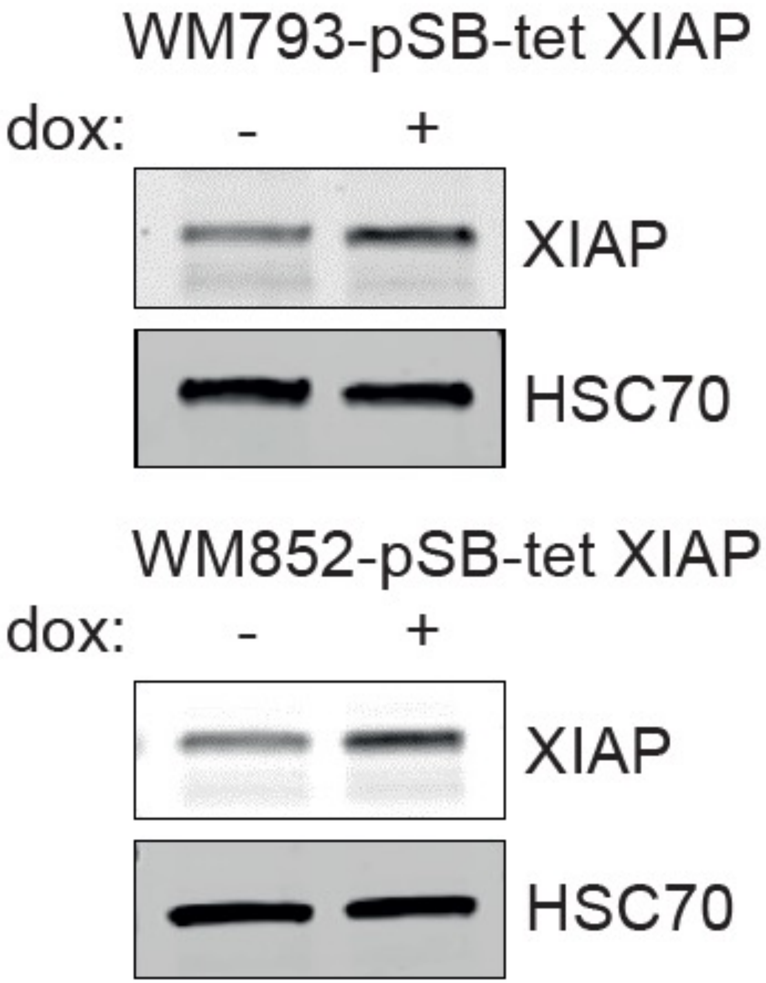

Figure S2R *Article version*

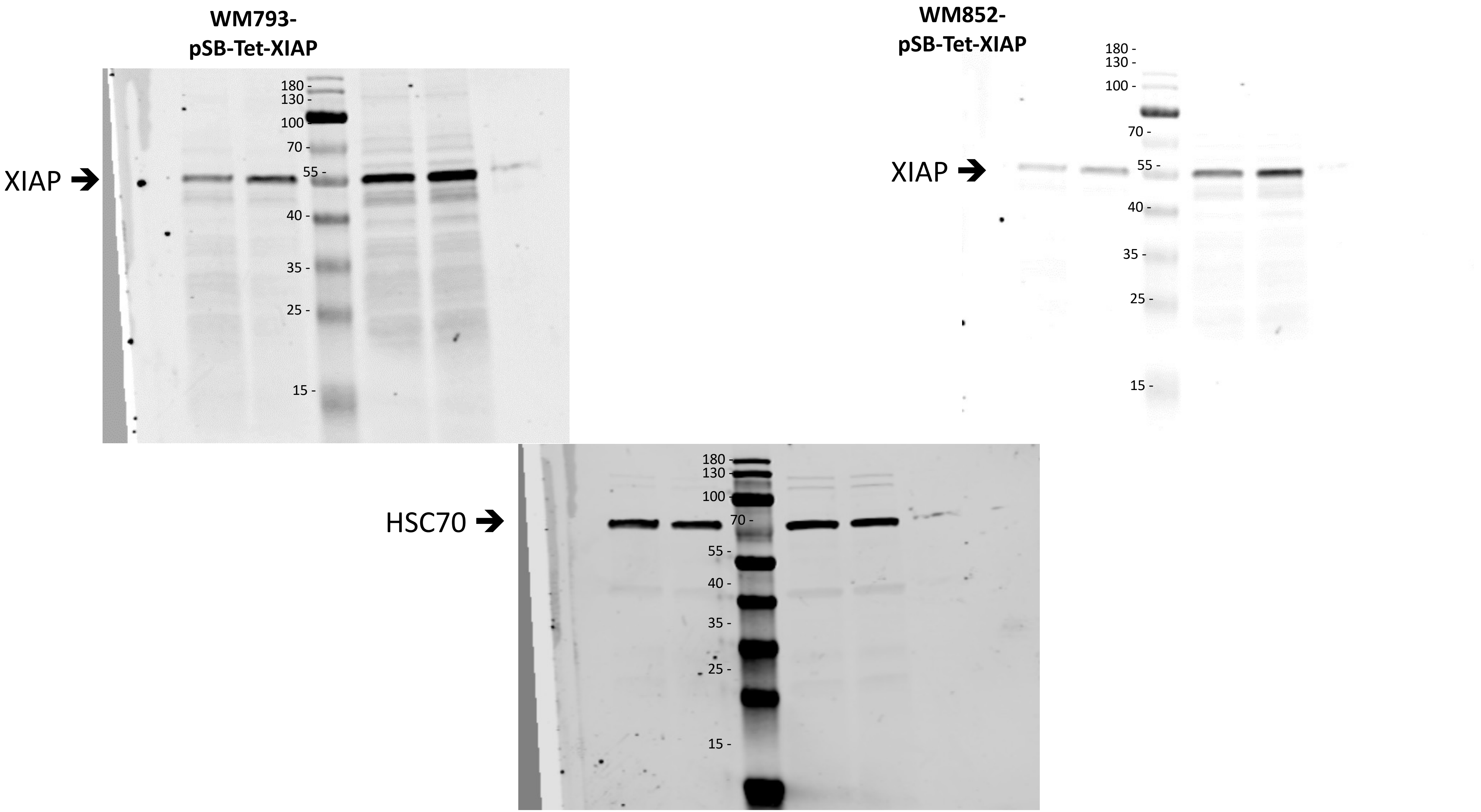

Figure S3B *Article version*

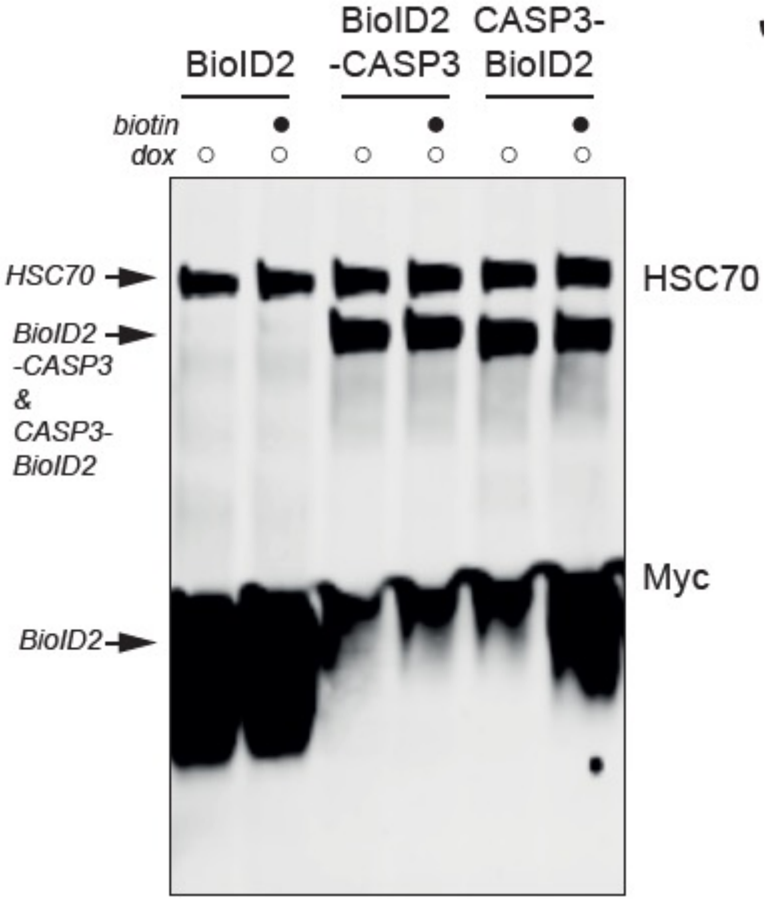

Figure S3B ***Raw data***

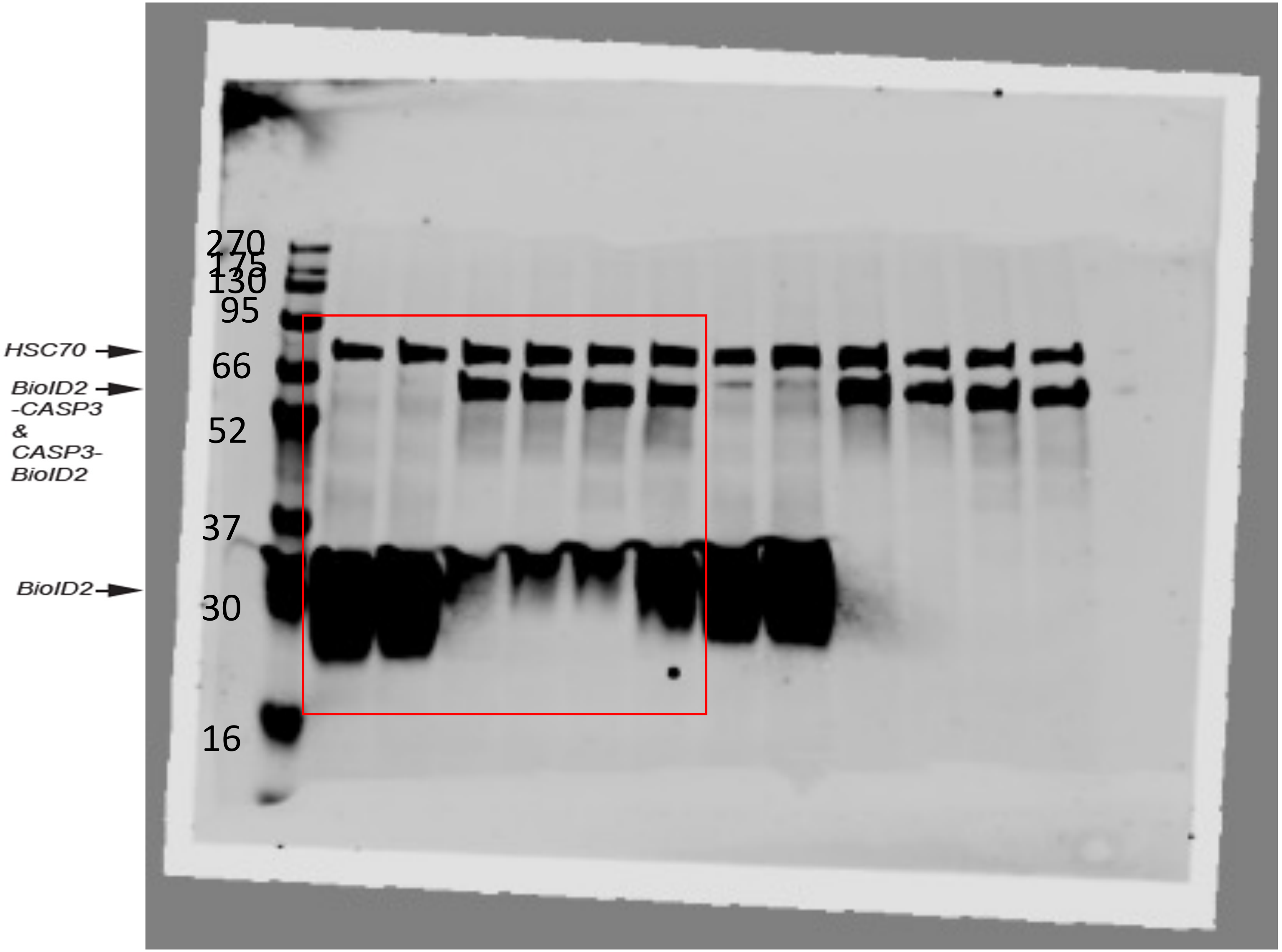

Figure S3C *Article version*

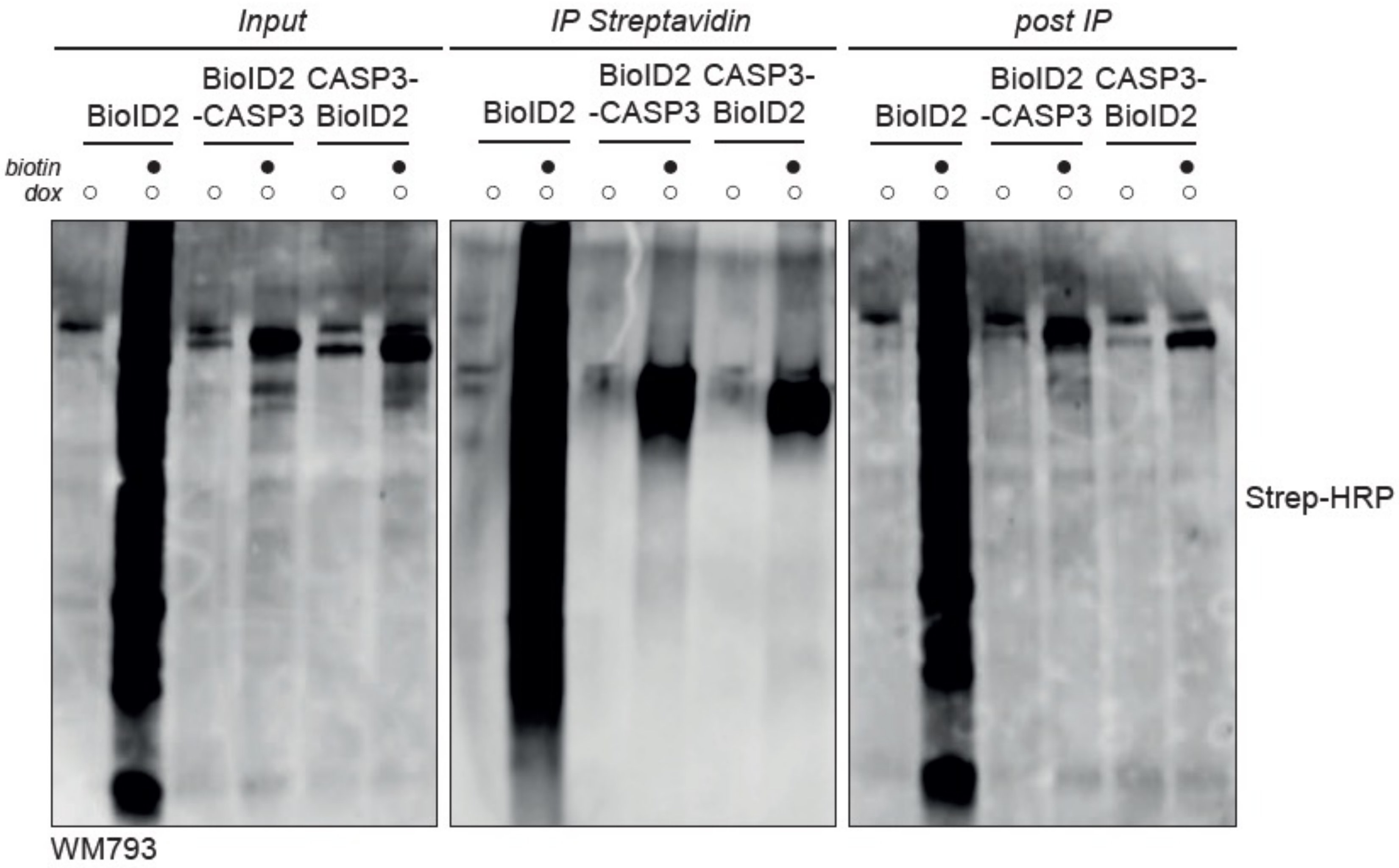

Figure S3C *Raw data*

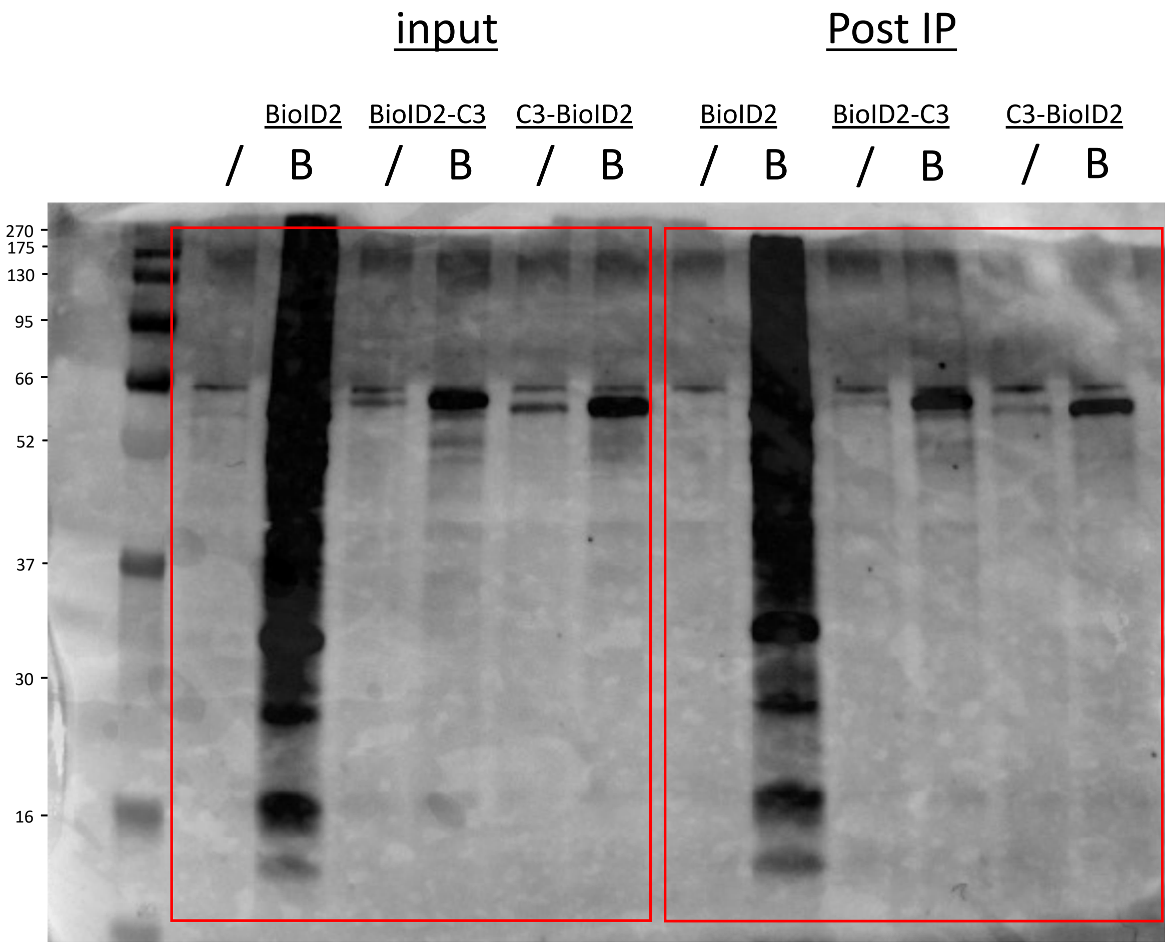

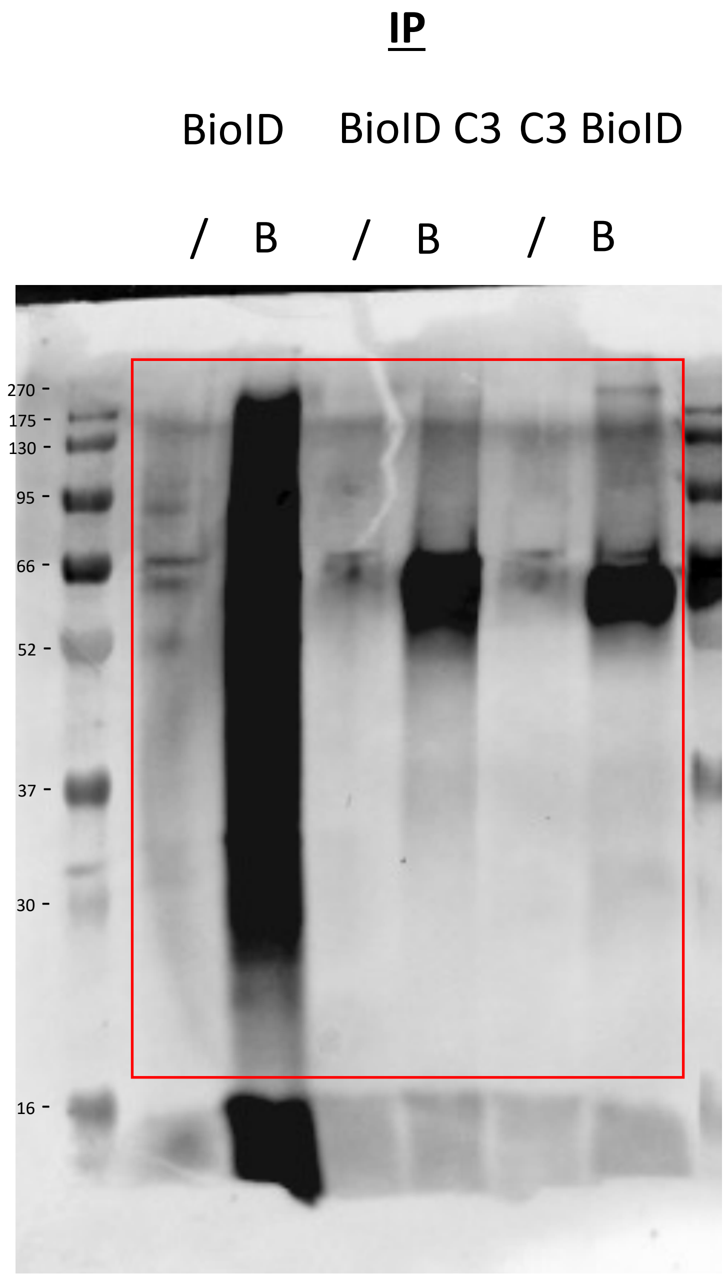

WM793

Figure S3D *Article version*

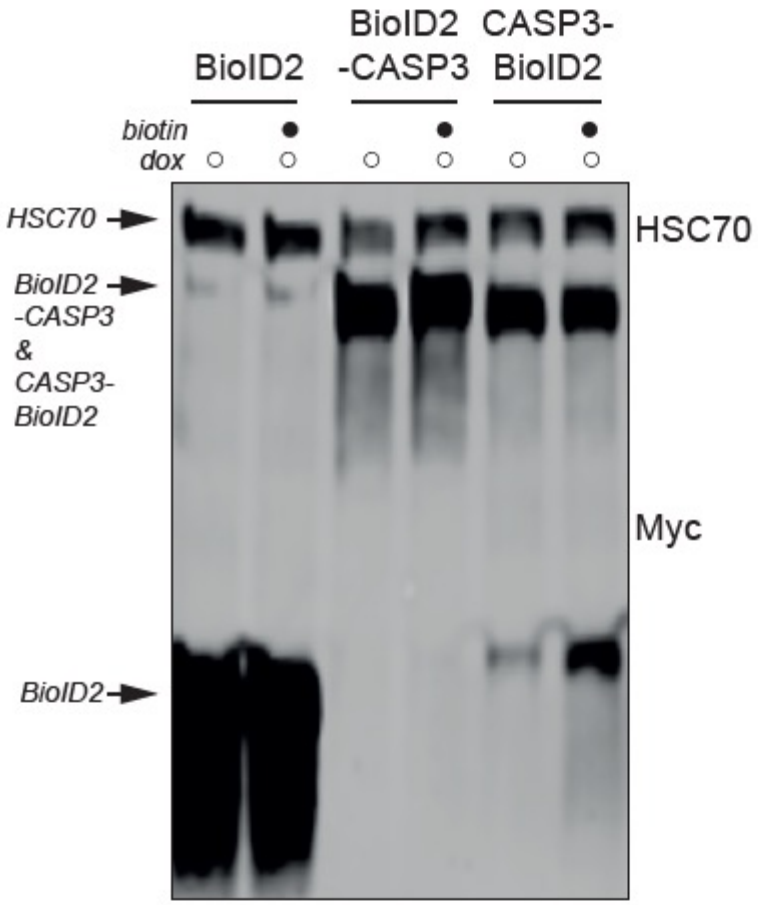

Figure S3D *Raw data*

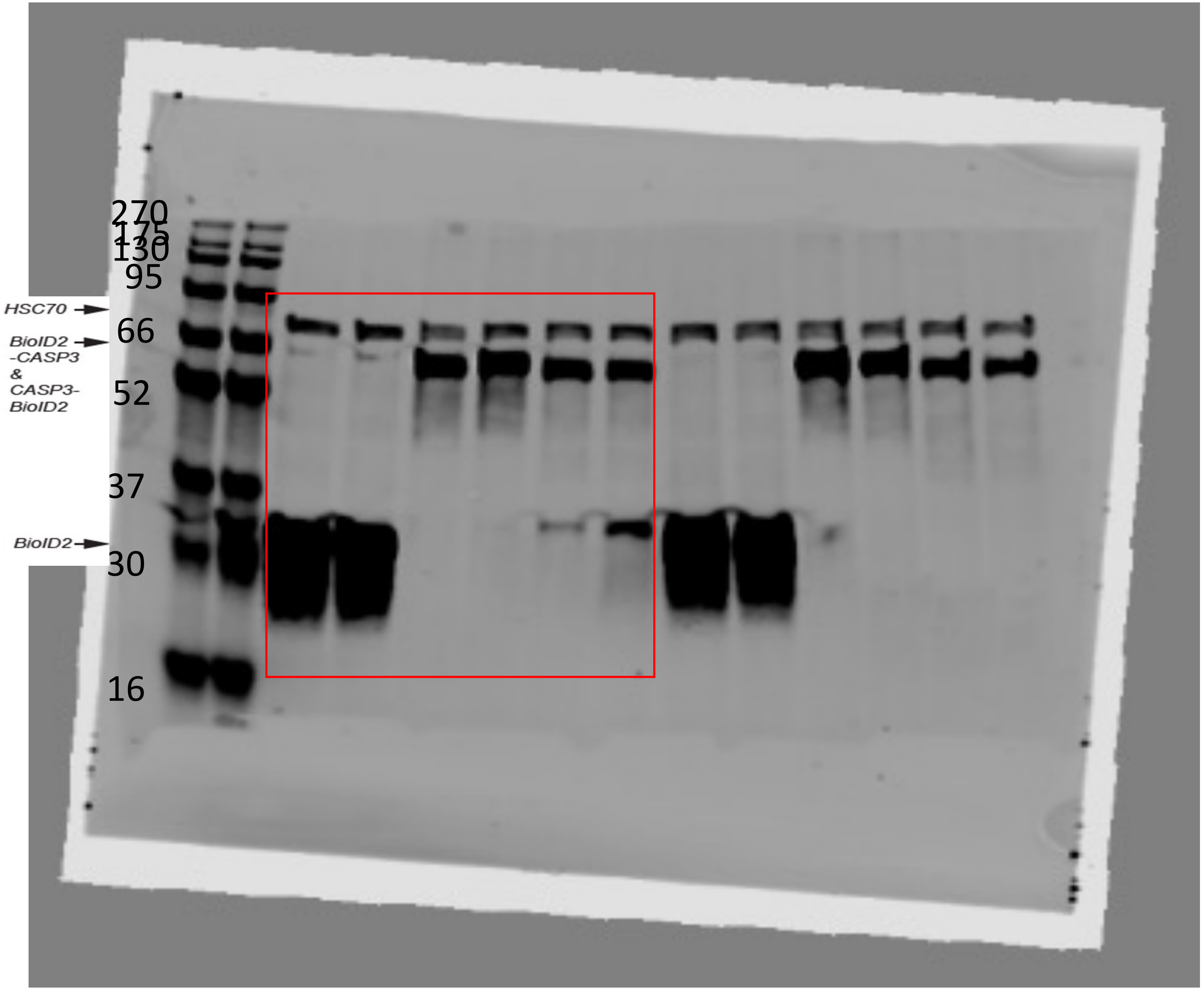

Figure S3E *Article version*

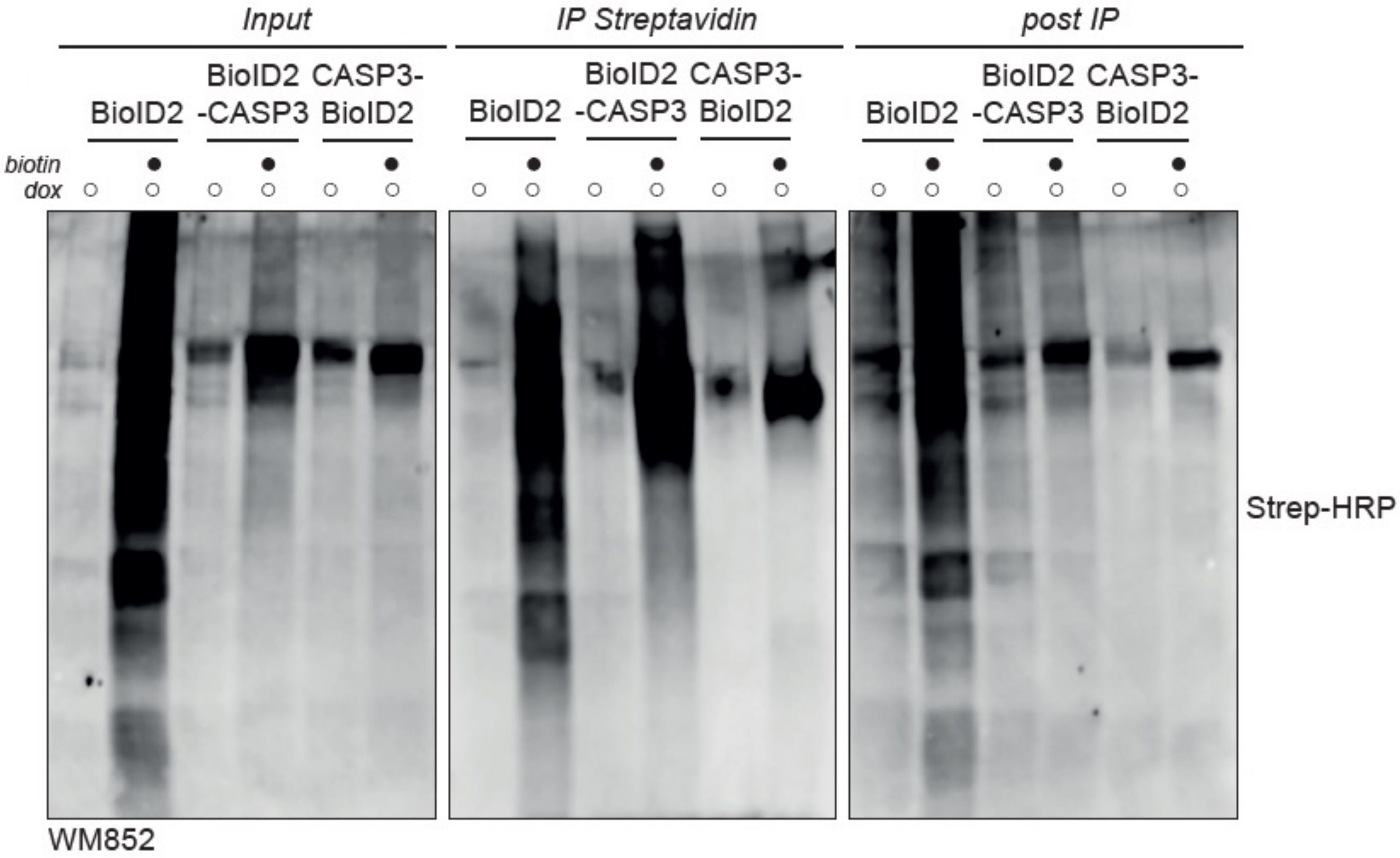

Figure S3E ***Raw data***

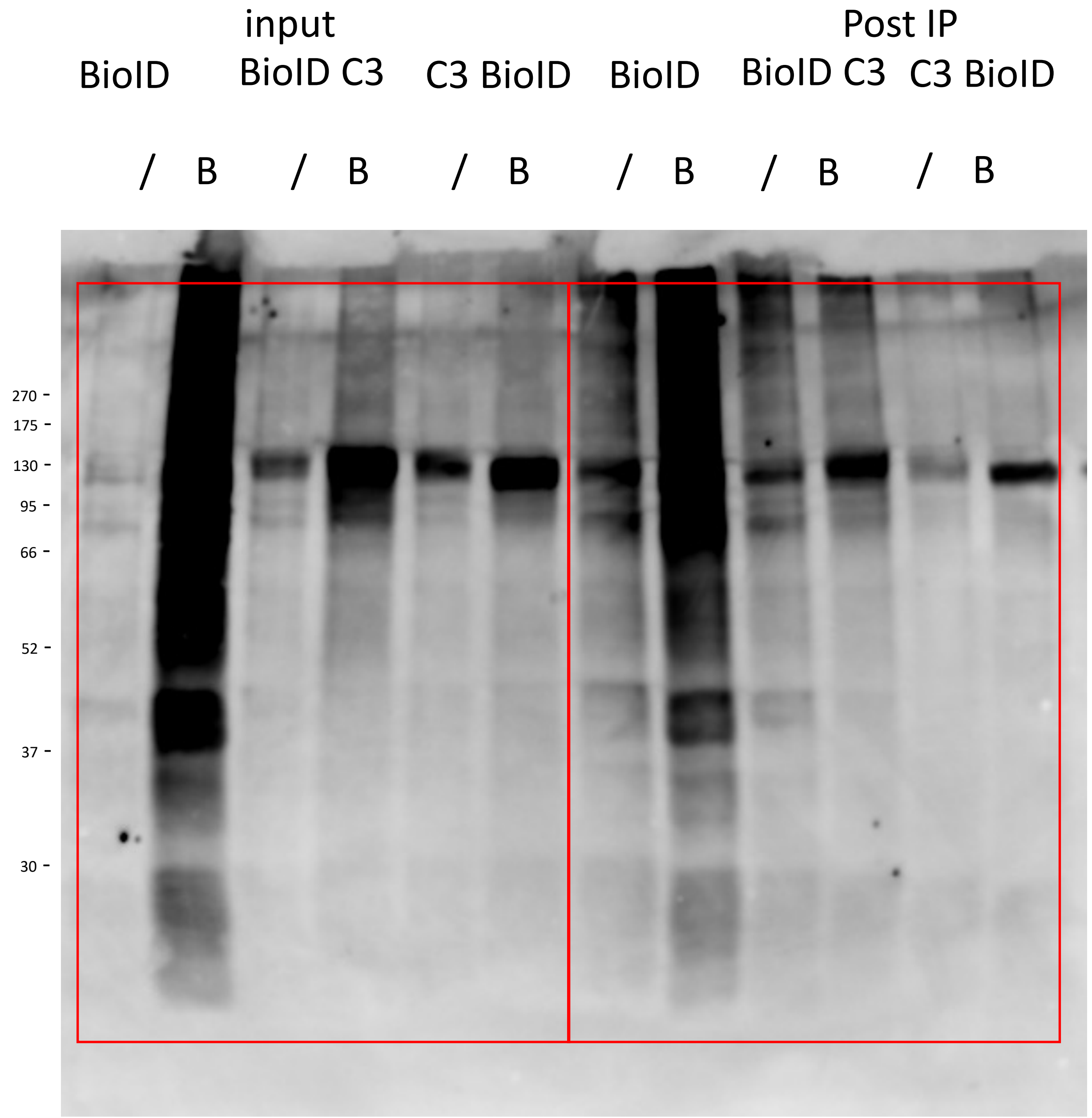

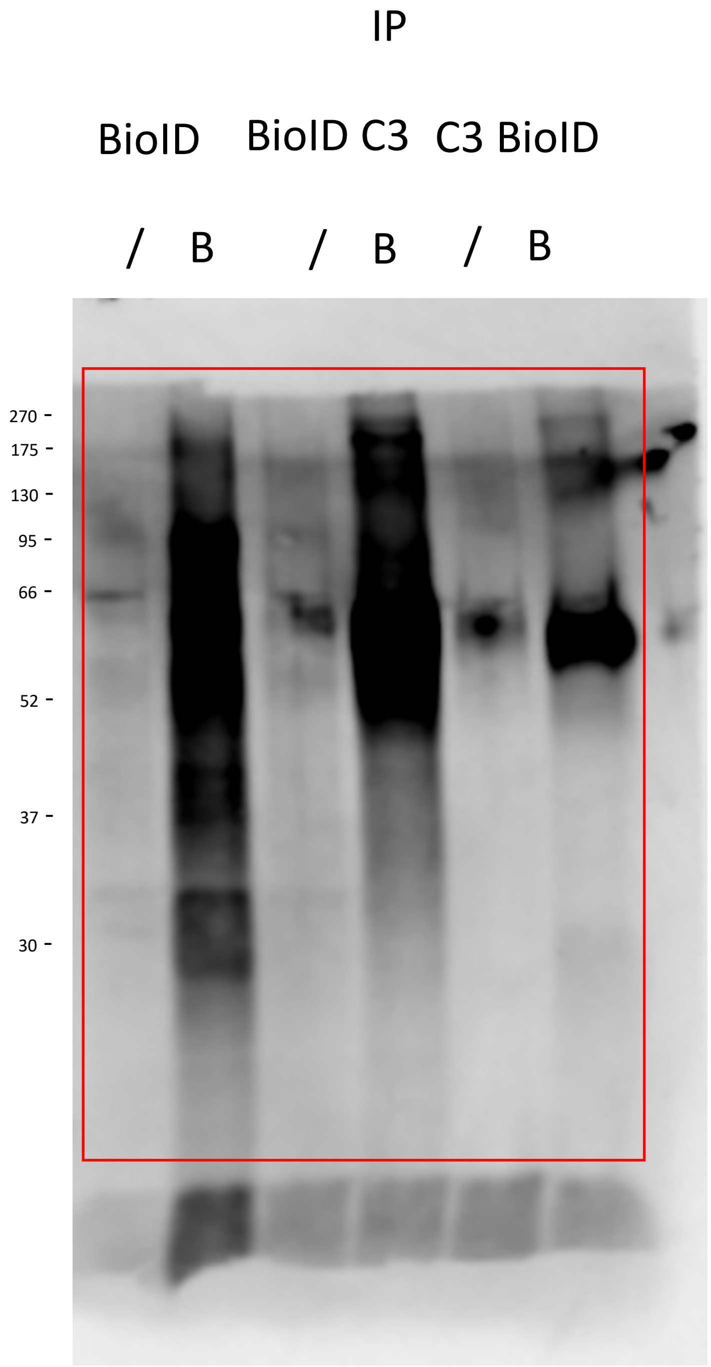

Figure S3G *Article version*

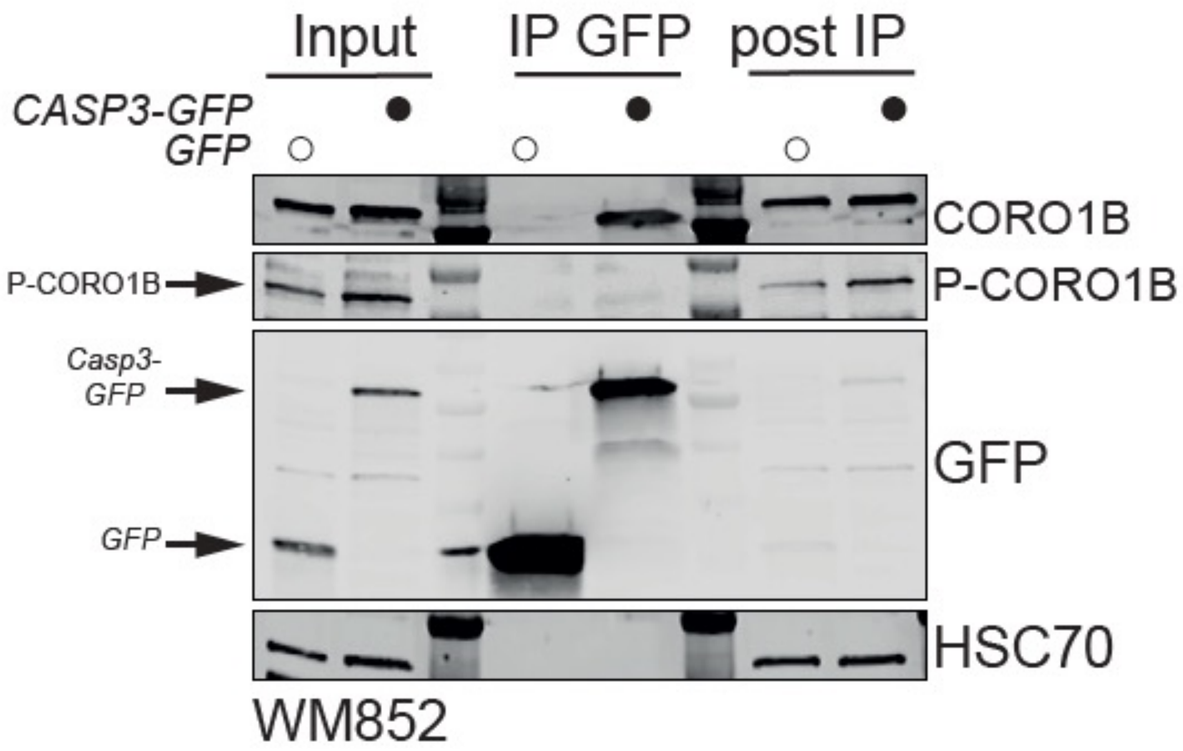

Figure S3G *Raw data*

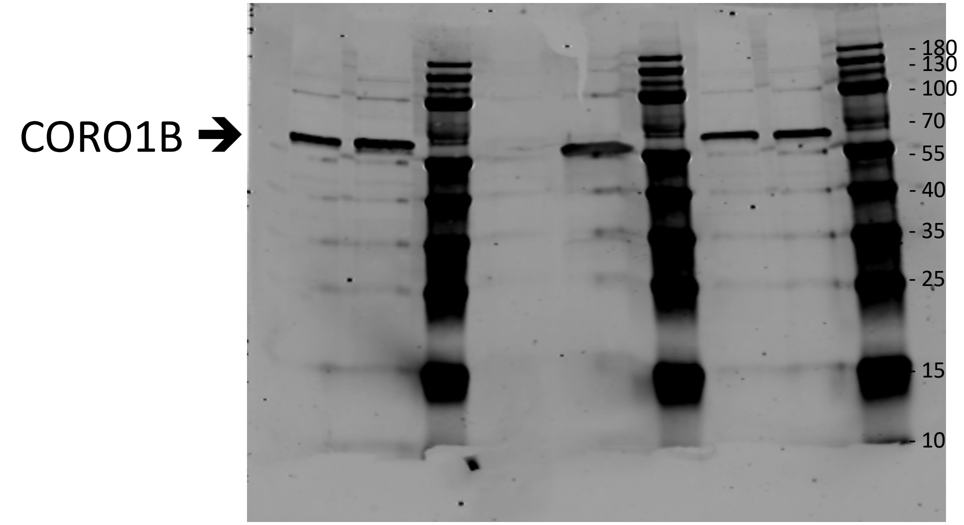

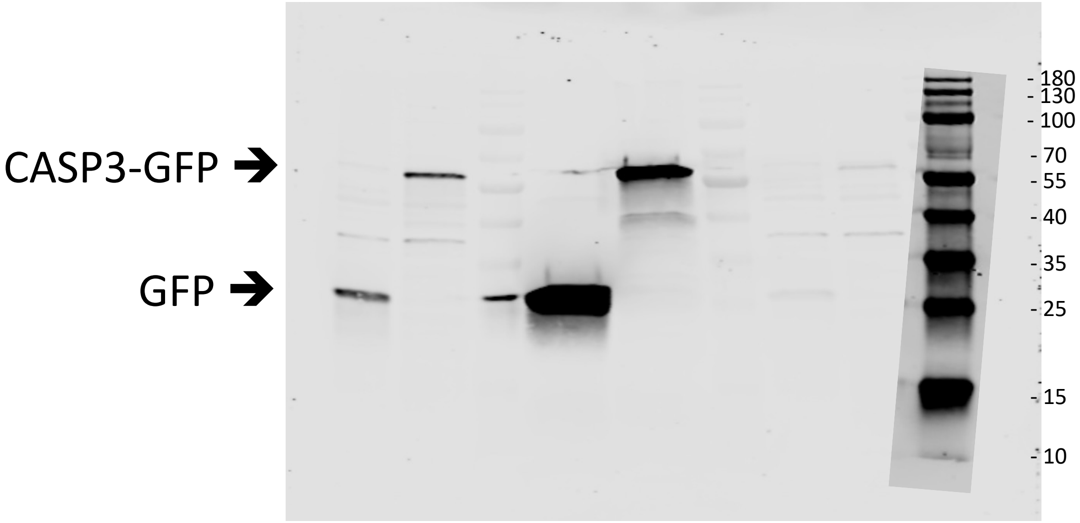

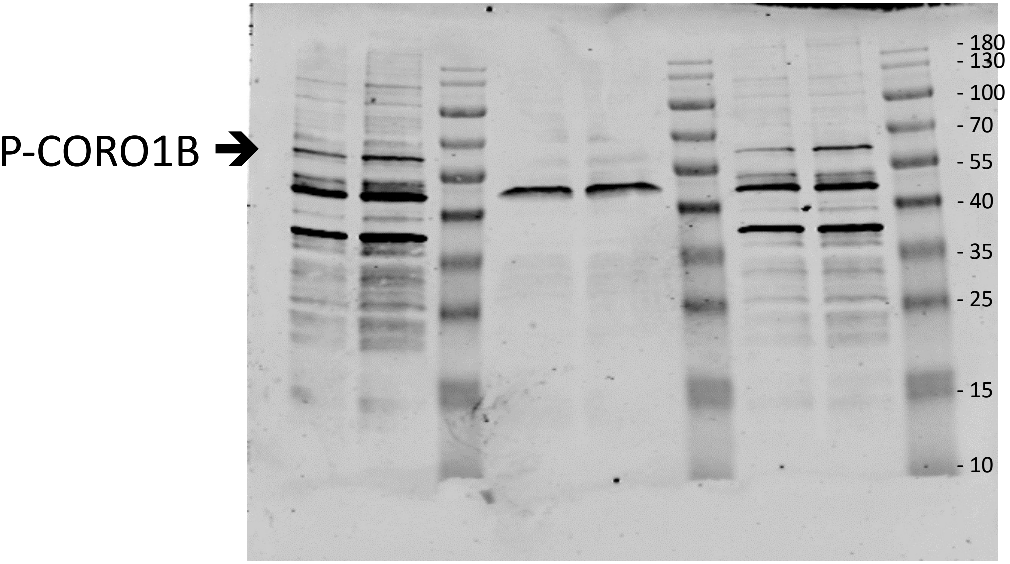

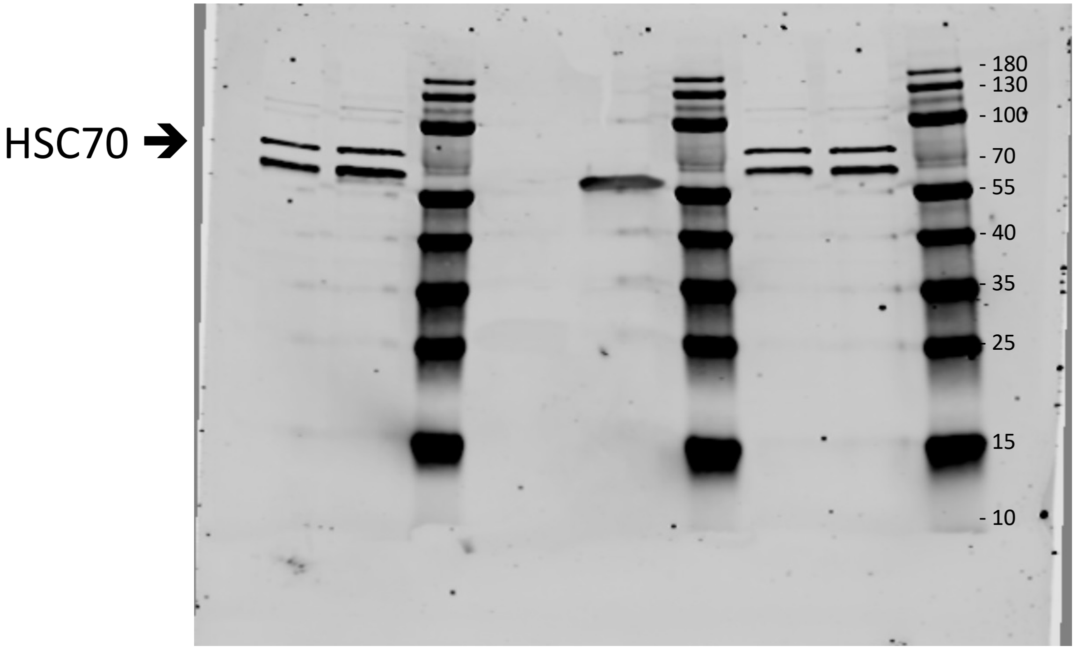

Figure S3J *Article version*

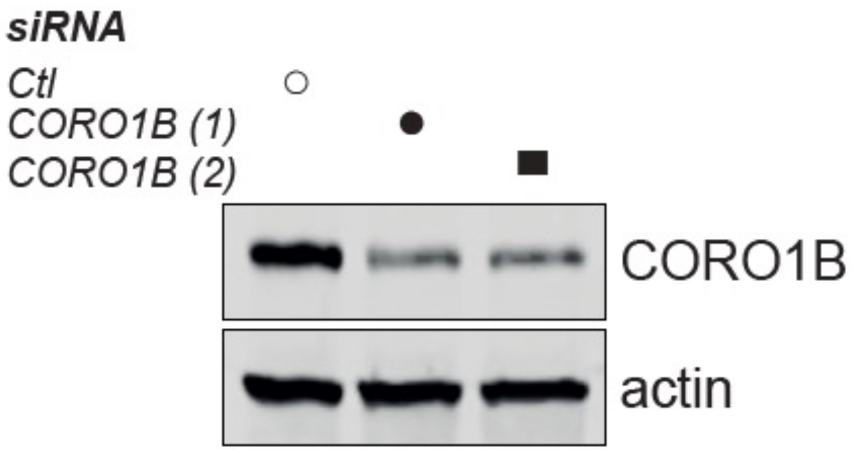

Figure S3J *Raw data*

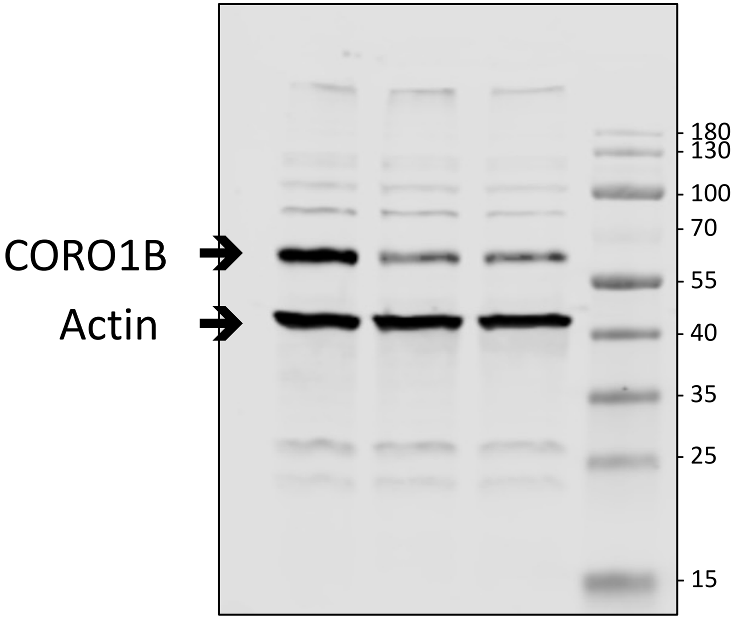

Figure S3K *Article version*

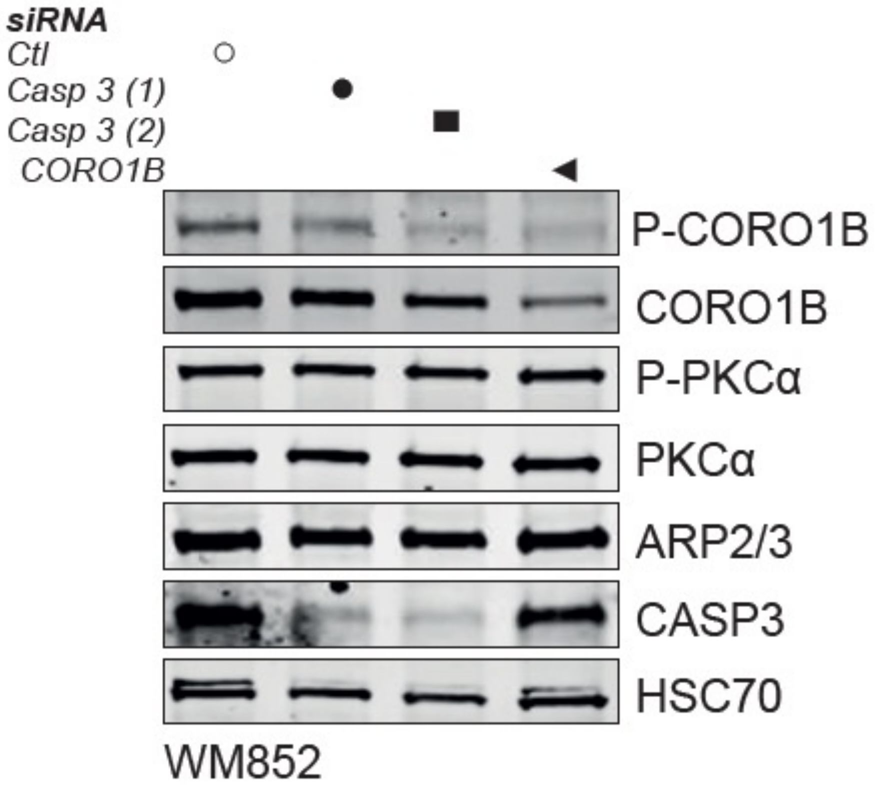

Figure S3K *Raw data*

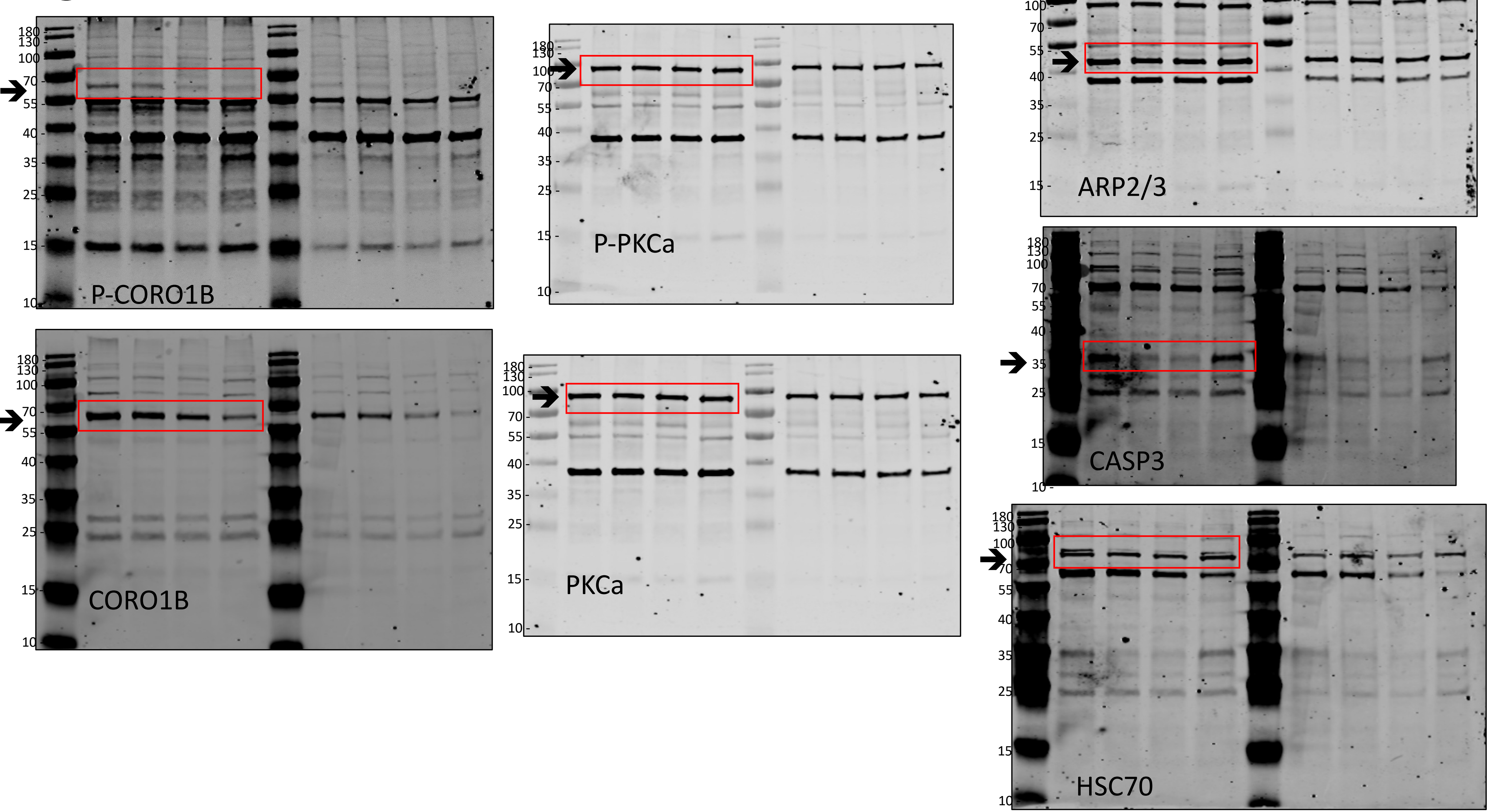

Figure S3N *Article version*

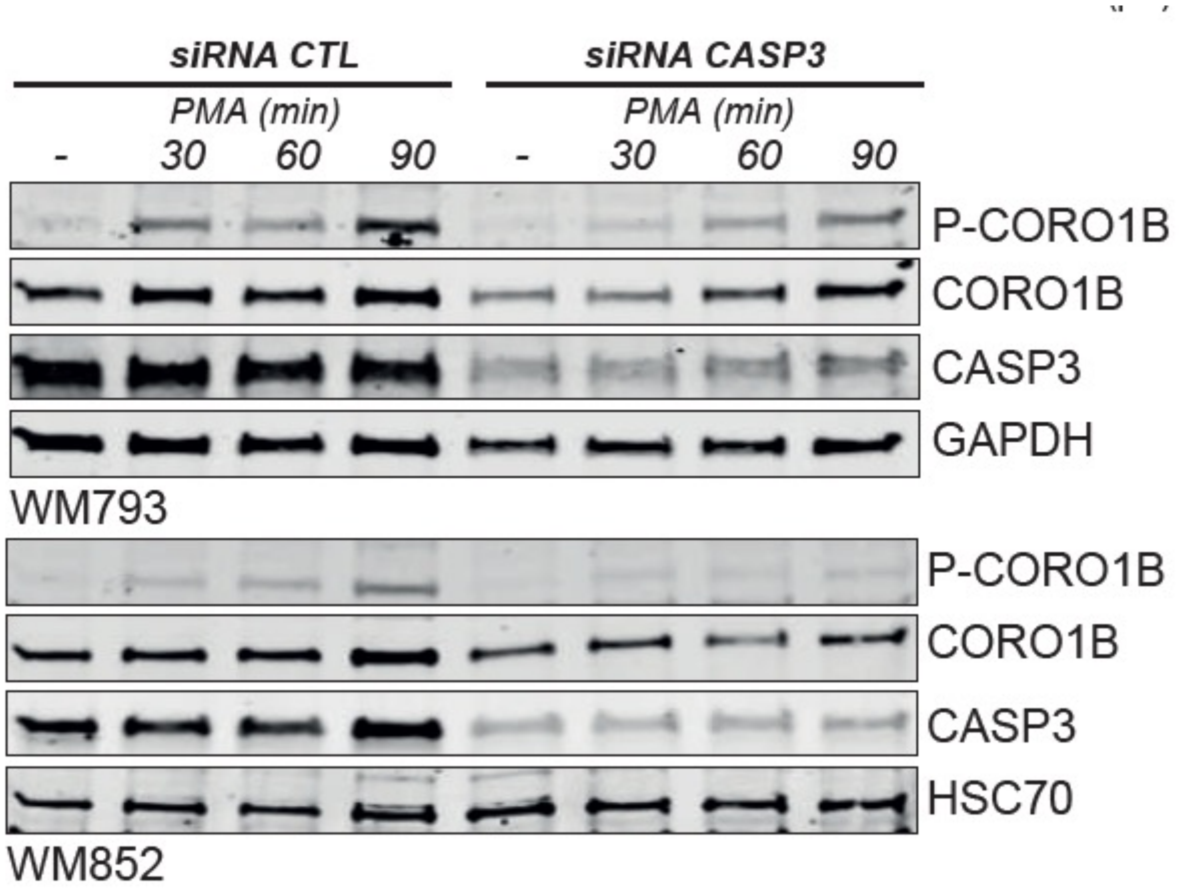

Figure S3N *Raw data*

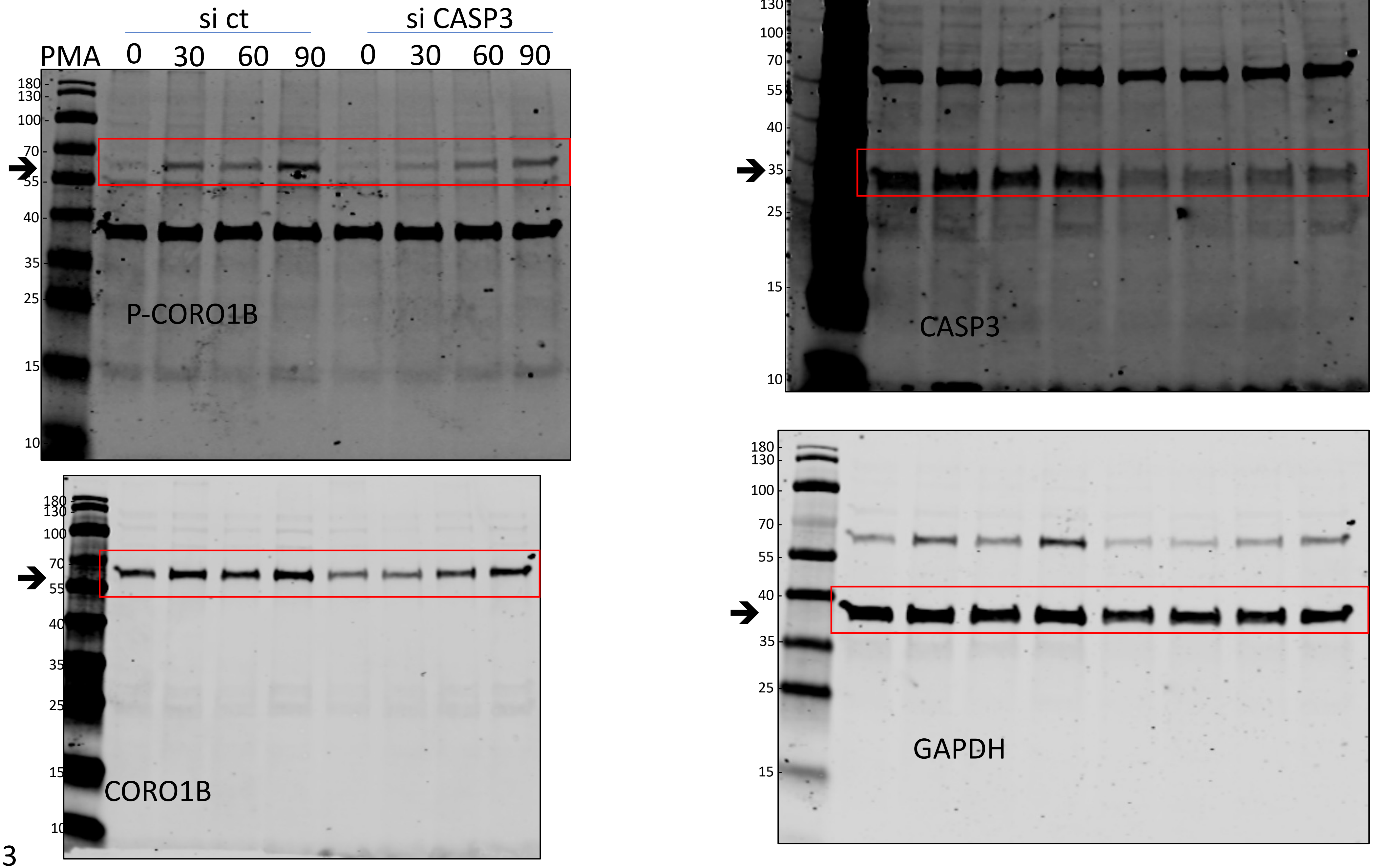

WM793

Figure S3N *Raw data*

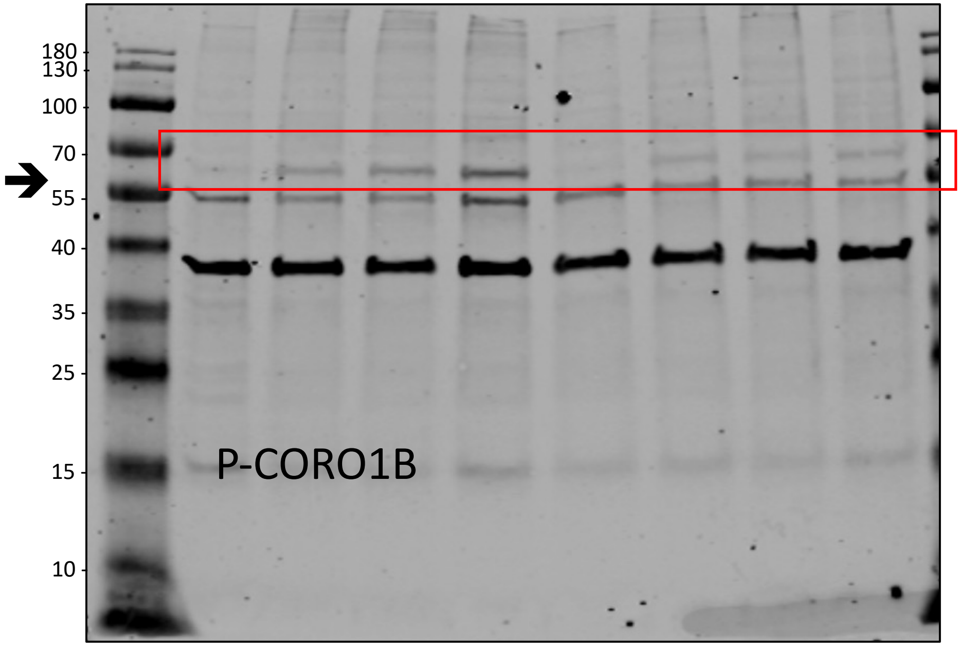

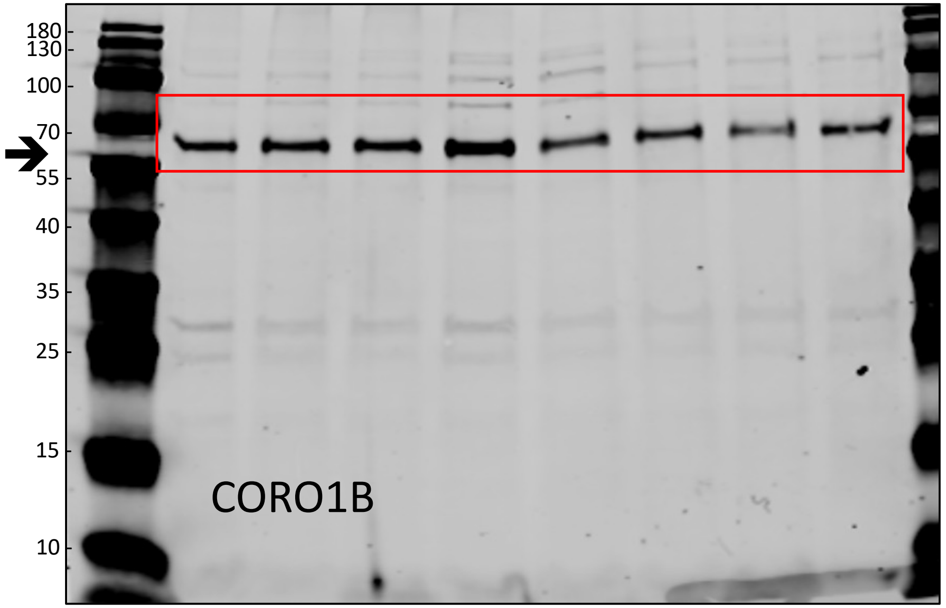

WM852

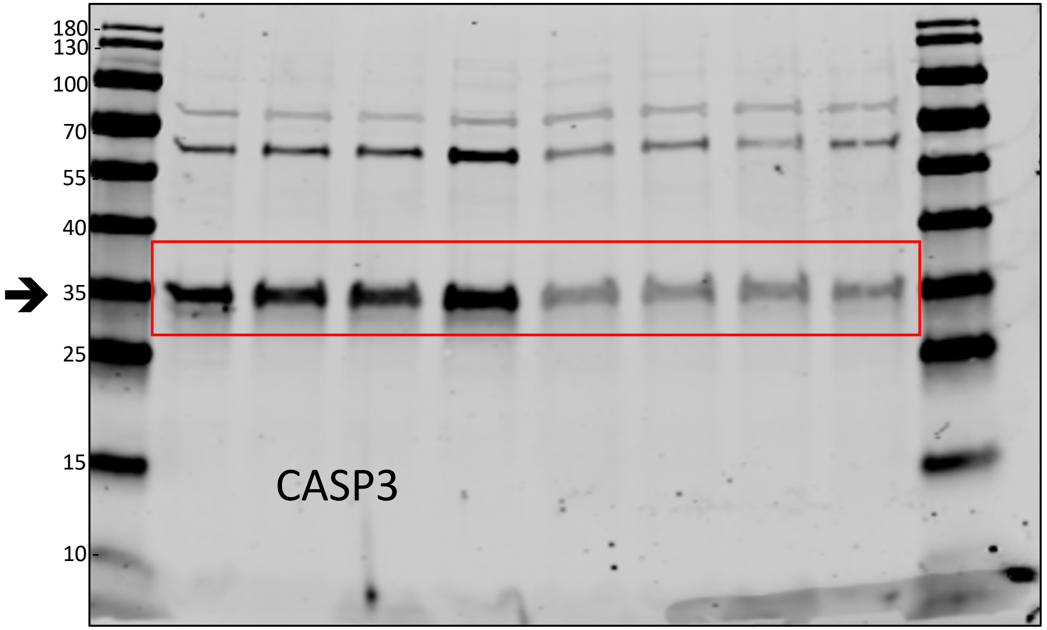

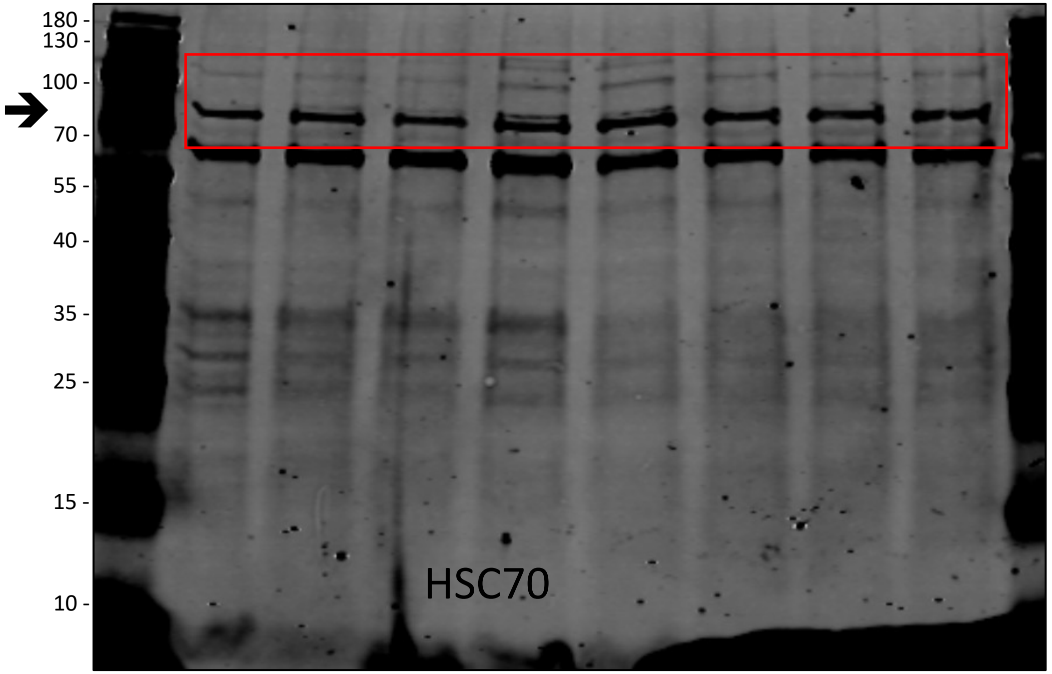

Figure S3O *Article version*

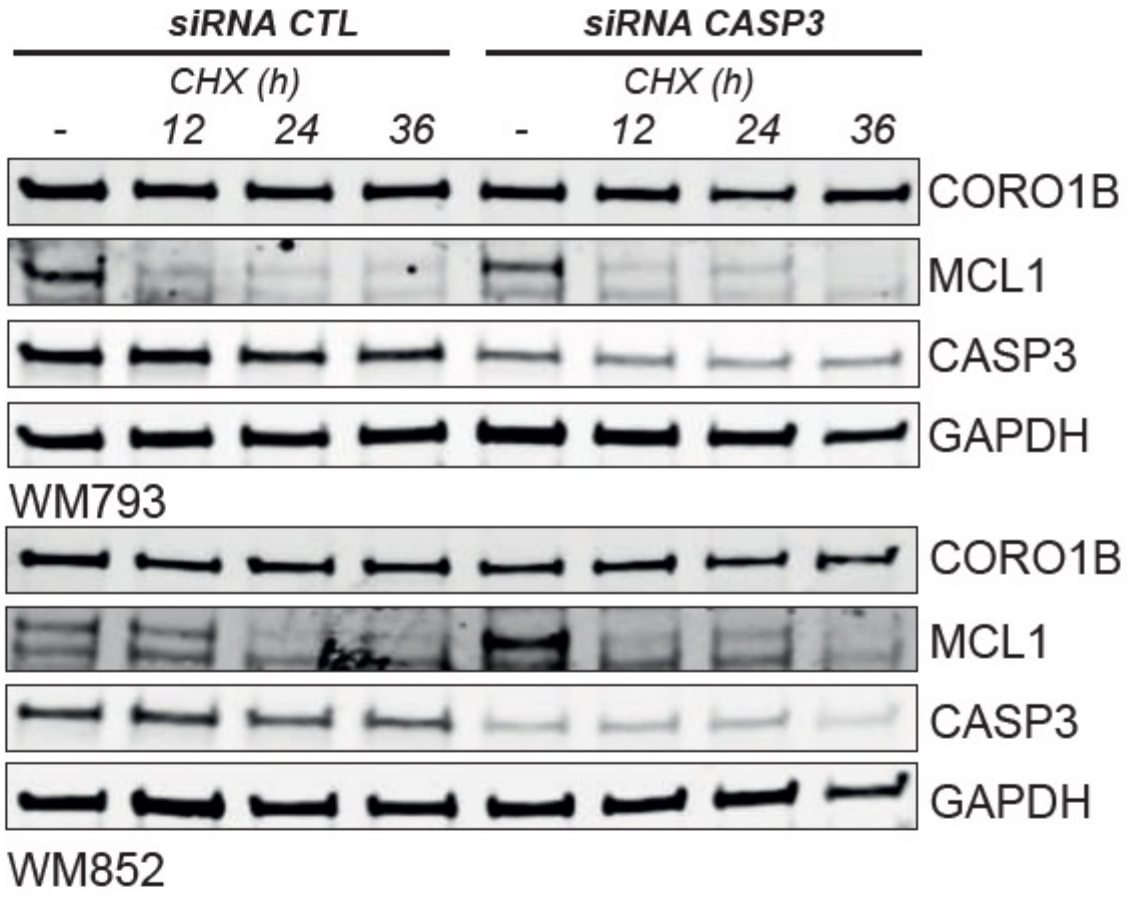

Figure S3O *Raw data*

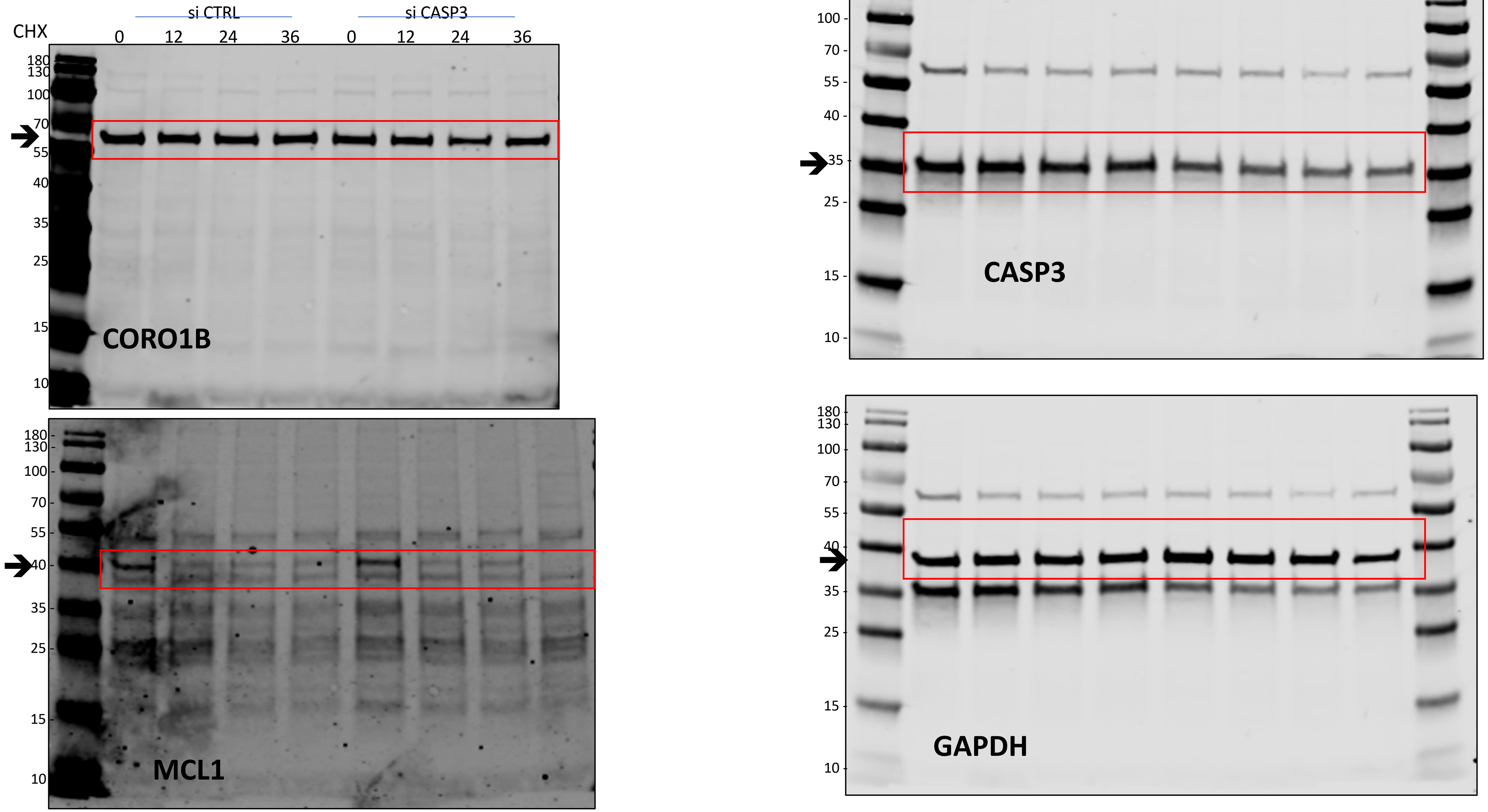

WM793

Figure S3O *Raw data*

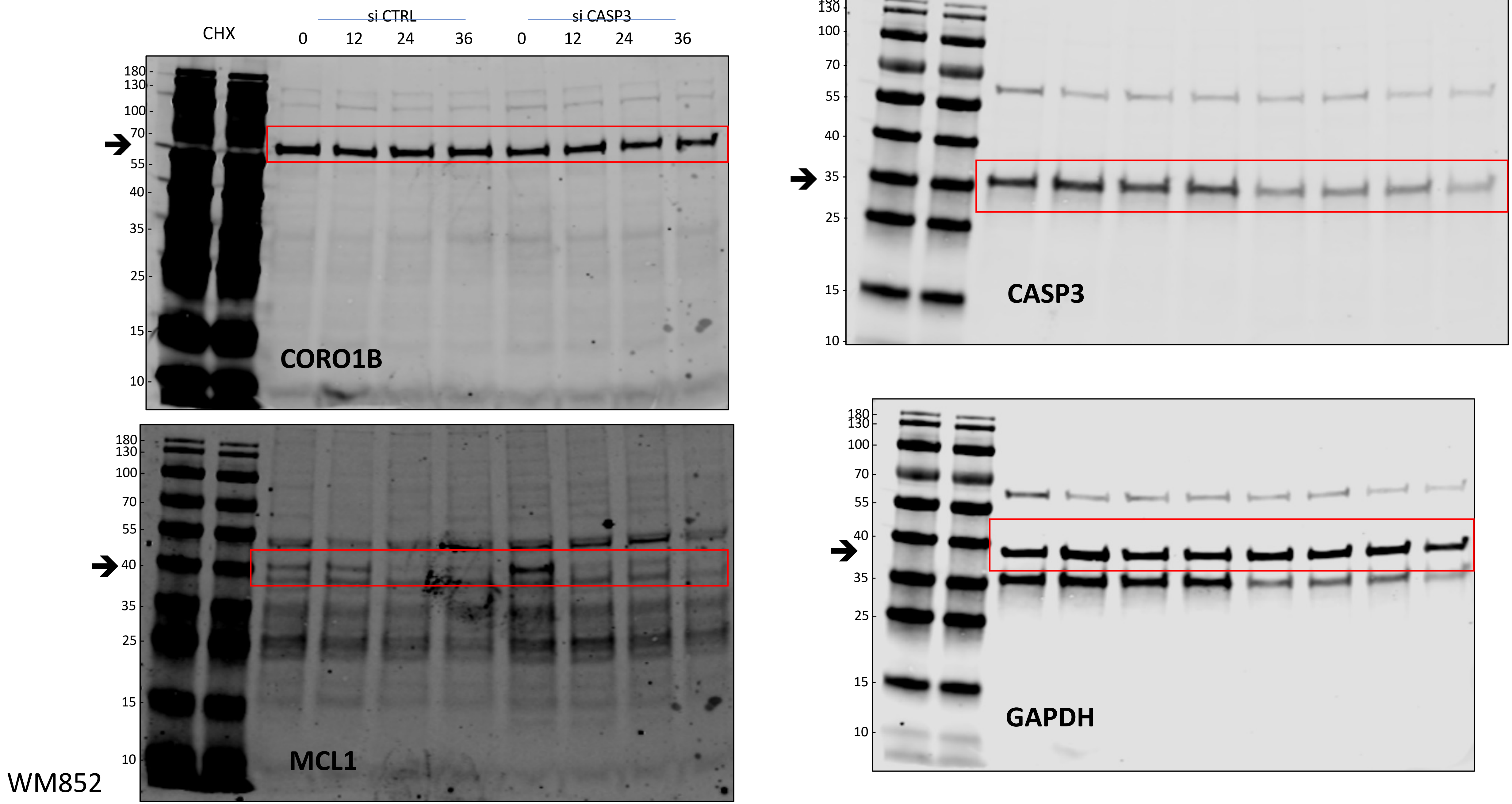

Figure S4B *Article version*

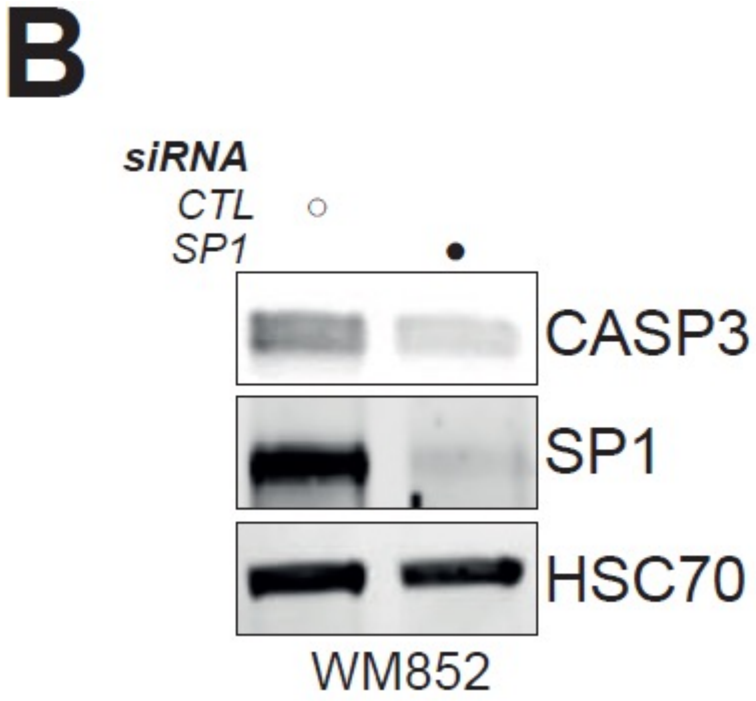

Figure S4B *Raw data*

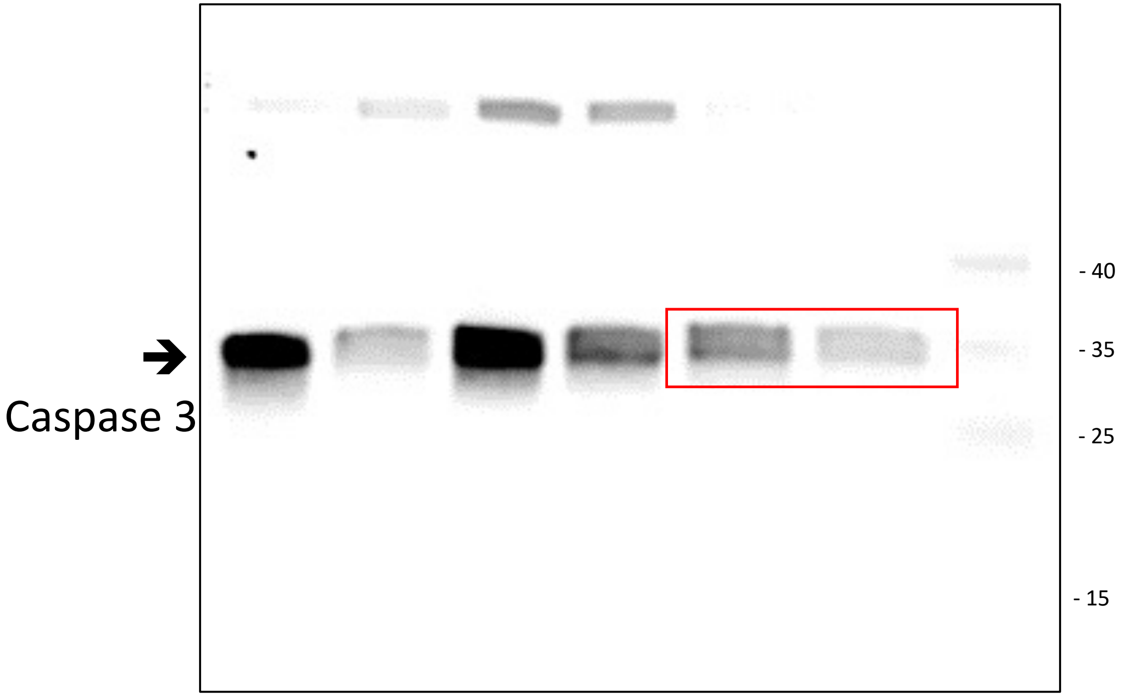

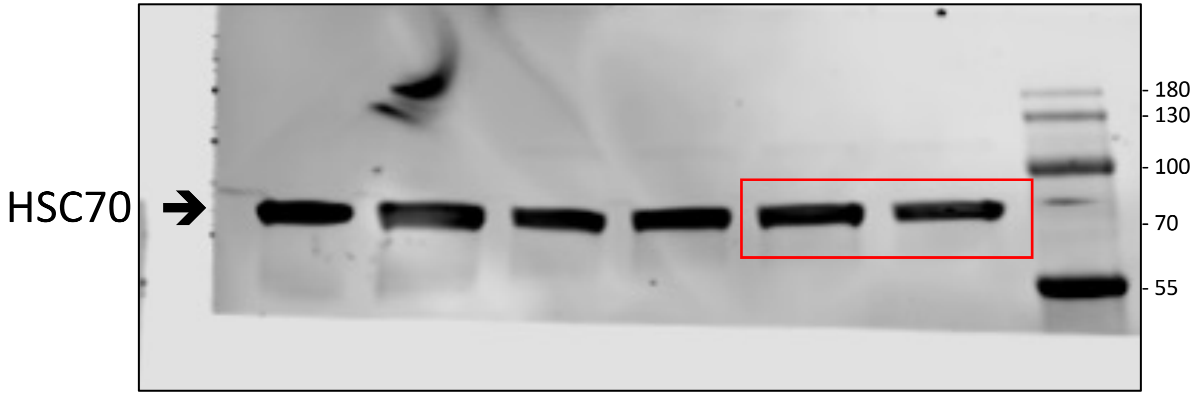

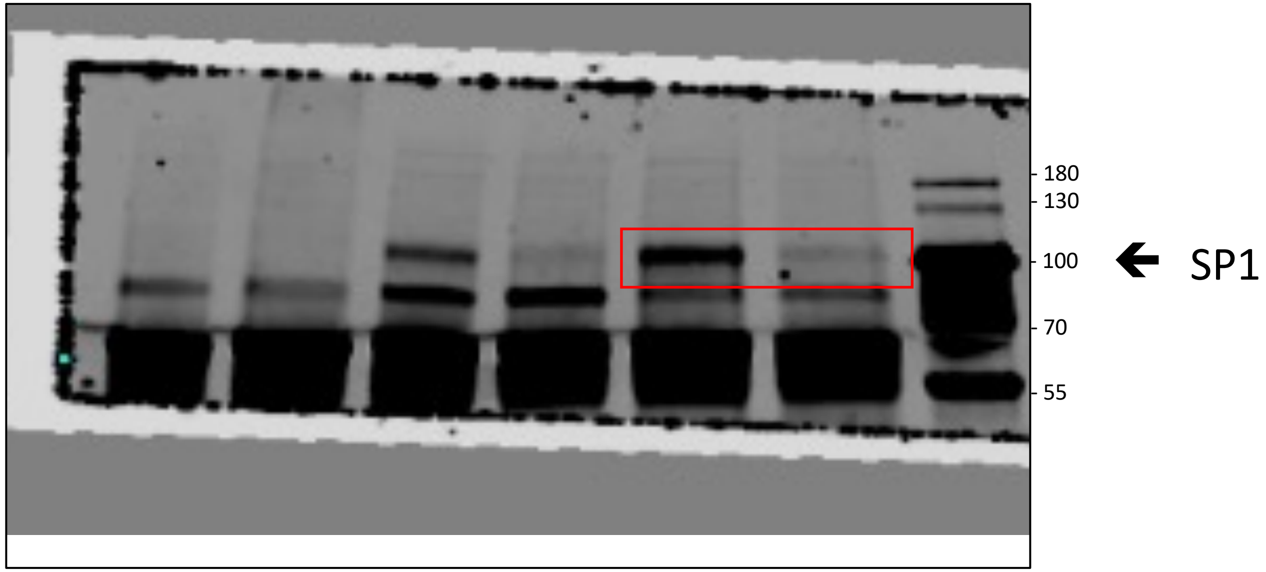

Supplement: Supplementary file 6 — uncropped western blots [file 41419_2025_7952_MOESM6_ESM.pdf]
